# Effects of rare kidney diseases on kidney failure: a longitudinal analysis of the UK National Registry of Rare Kidney Diseases (RaDaR) cohort

**DOI:** 10.1016/S0140-6736(23)02843-X

**Published:** 2024-03-30

**Authors:** Katie Wong, David Pitcher, Fiona Braddon, Lewis Downward, Retha Steenkamp, Nicholas Annear, Jonathan Barratt, Coralie Bingham, Constantina Chrysochou, Richard J Coward, David Game, Sian Griffin, Matt Hall, Sally Johnson, Durga Kanigicherla, Fiona Karet Frankl, David Kavanagh, Larissa Kerecuk, Eamonn R Maher, Shabbir Moochhala, Jenny Pinney, John A Sayer, Roslyn Simms, Smeeta Sinha, Shalabh Srivastava, Frederick W K Tam, Andrew Neil Turner, Stephen B Walsh, Aoife Waters, Patricia Wilson, Edwin Wong, Christopher Mark Taylor, Dorothea Nitsch, Moin Saleem, Detlef Bockenhauer, Kate Bramham, Daniel P Gale, RaDaR consortium, Sharirose Abat, Shazia Adalat, Joy Agbonmwandolor, Zubaidah Ahmad, Abdulfattah Alejmi, Rashid Almasarwah, Nicholas Annear, Ellie Asgari, Amanda Ayers, Jyoti Baharani, Gowrie Balasubramaniam, Felix Kpodo, Tarun Bansal, Alison Barratt, Jonathan Barratt, Megan Bates, Natalie Bayne, Janet Bendle, Sarah Benyon, Carsten Bergmann, Sunil Bhandari, Coralie Bingham, Preetham Boddana, Sally Bond, Fiona Braddon, Kate Bramham, Angela Branson, Stephen Brearey, Vicky Brocklebank, Sharanjit Budwal, Conor Byrne, Hugh Cairns, Brian Camilleri, Gary Campbell, Alys Capell, Margaret Carmody, Marion Carson, Tracy Cathcart, Christine Catley, Karine Cesar, Melanie Chan, Houda Chea, James Chess, Chee Kay Cheung, Katy-Jane Chick, Nihil Chitalia, Martin Christian, Tina Chrysochou, Katherine Clark, Christopher Clayton, Rhian Clissold, Helen Cockerill, Joshua Coelho, Elizabeth Colby, Viv Colclough, Eileen Conway, H Terence Cook, Wendy Cook, Theresa Cooper, Richard J Coward, Sarah Crosbie, Gabor Cserep, Anjali Date, Katherine Davidson, Amanda Davies, Neeraj Dhaun, Ajay Dhaygude, Lynn Diskin, Abhijit Dixit, Eunice Doctolero, Suzannah Dorey, Lewis Downard, Mark Drayson, Gavin Dreyer, Tina Dutt, Kufreabasi Etuk, Dawn Evans, Jenny Finch, Frances Flinter, James Fotheringham, Lucy Francis, Daniel P Gale, Hugh Gallagher, David Game, Eva Garcia, Madita Gavrila, Susie Gear, Colin Geddes, Mark Gilchrist, Matt Gittus, Paraskevi Goggolidou, Christopher Goldsmith, Patricia Gooden, Andrea Goodlife, Priyanka Goodwin, Tassos Grammatikopoulos, Barry Gray, Megan Griffith, Steph Gumus, Sanjana Gupta, Patrick Hamilton, Lorraine Harper, Tess Harris, Louise Haskell, Samantha Hayward, Shivaram Hegde, Bruce Hendry, Sue Hewins, Nicola Hewitson, Kate Hillman, Mrityunjay Hiremath, Alexandra Howson, Zay Htet, Sharon Huish, Richard Hull, Alister Humphries, David P J Hunt, Karl Hunter, Samantha Hunter, Marilyn Ijeomah-Orji, Nick Inston, David Jayne, Gbemisola Jenfa, Alison Jenkins, Sally Johnson, Caroline A Jones, Colin Jones, Amanda Jones, Rachel Jones, Lavanya Kamesh, Durga Kanigicherla, Fiona Karet Frankl, Mahzuz Karim, Amrit Kaur, David Kavanagh, Kelly Kearley, Larissa Kerecuk, Arif Khwaja, Garry King, Grant King, Ewa Kislowska, Edyta Klata, Maria Kokocinska, Mark Lambie, Laura Lawless, Thomas Ledson, Rachel Lennon, Adam P Levine, Ling Wai Maggie Lai, Graham Lipkin, Graham Lovitt, Paul Lyons, Holly Mabillard, Katherine Mackintosh, Khalid Mahdi, Eamonn Maher, Kevin J Marchbank, Patrick B Mark, Sherry Masoud, Bridgett Masunda, Zainab Mavani, Jake Mayfair, Stephen McAdoo, Joanna Mckinnell, Nabil Melhem, Simon Meyrick, Shabbir Moochhala, Putnam Morgan, Ann Morgan, Fawad Muhammad, Shona Murray, Kristina Novobritskaya, Albert CM Ong, Louise Oni, Kate Osmaston, Neal Padmanabhan, Sharon Parkes, Jean Patrick, James Pattison, Riny Paul, Rachel Percival, Stephen J Perkins, Alexandre Persu, William G Petchey, Matthew C Pickering, Jennifer Pinney, David Pitcher, Lucy Plumb, Zoe Plummer, Joyce Popoola, Frank Post, Albert Power, Guy Pratt, Charles Pusey, Ria Rabara, May Rabuya, Tina Raju, Chadd Javier, Ian S D Roberts, Candice Roufosse, Adam Rumjon, Alan Salama, Moin Saleem, Richard Sandford, Kanwaljit S Sandu, Nadia Sarween, John A Sayer, Neil Sebire, Haresh Selvaskandan, Asheesh Sharma, Edward J Sharples, Neil Sheerin, Harish Shetty, Rukshana Shroff, Roslyn Simms, Manish Sinha, Smeeta Sinha, Kerry Smith, Lara Smith, Shalabh Srivastava, Retha Steenkamp, Ian Stott, Katerina Stroud, Pauline Swift, Justyna Szklarzewicz, Fred Tam, Kay Tan, Robert Taylor, Marc Tischkowitz, Kay Thomas, Yincent Tse, Alison Turnbull, A Neil Turner, Kay Tyerman, Miranda Usher, Gopalakrishnan Venkat-Raman, Alycon Walker, Stephen B Walsh, Aoife Waters, Angela Watt, Phil Webster, Ashutosh Wechalekar, Gavin I Welsh, Nicol West, David Wheeler, Kate Wiles, Lisa Willcocks, Angharad Williams, Emma Williams, Karen Williams, Deborah H Wilson, Patricia D Wilson, Paul Winyard, Edwin Wong, Katie Wong, Grahame Wood, Emma Woodward, Len Woodward, Adrian Woolf, David Wright

**Affiliations:** a National Registry of Rare Kidney Diseases, Bristol, UK; b Department of Renal Medicine, University College London, London, UK; c UK Renal Registry, Bristol, UK; d Institute of Medical and Biomedical Education, St George's University of London, London, UK; e Department of Cardiovascular Sciences, University of Leicester, Leicester, UK; f University of Exeter Medical School, University of Exeter, Exeter, UK; g Translational Health Sciences, University of Bristol, Bristol, UK; h Division of Cardiovascular Sciences, University of Manchester, Manchester, UK; i Guy's and St Thomas’ NHS Foundation Trust, London, UK; j Department of Nephrology, University Hospital Wales, Cardiff, UK; k Nottingham Renal and Transplant Unit, Nottingham University Hospitals NHS Foundation Trust, Nottingham, UK; l Great North Children's Hospital, Newcastle upon Tyne, UK; m Cambridge Institute for Medical Research, University of Cambridge, Cambridge, UK; n Department of Medical Genetics, University of Cambridge, Cambridge, UK; o National Renal Complement Therapeutics Centre, Newcastle upon Tyne Hospitals NHS Foundation Trust, Newcastle upon Tyne, UK; p Complement Therapeutics Research Group, Newcastle University, Newcastle upon Tyne, UK; q Translational and Clinical Research Institute, Newcastle University, Newcastle upon Tyne, UK; r Birmingham Women's and Children's NHS Foundation Trust, Birmingham, UK; s Department of Renal Medicine, Royal Free London NHS Foundation Trust, London, UK; t Department of Renal Medicine, University Hospitals Birmingham NHS Foundation Trust, Birmingham, UK; u Academic Unit of Nephrology, Department of Infection, Immunity and Cardiovascular Disease, University of Sheffield, Sheffield, UK; v Department of Renal Medicine, Northern Care Alliance NHS Foundation Trust, Manchester, UK; w Department of Renal Medicine, South Tyneside and Sunderland NHS Foundation Trust, Sunderland, UK; x Centre for Inflammatory Disease, Department of Immunology and Inflammation, Imperial College London, London, UK; y Medical Research Council Centre for Inflammation, Edinburgh University, Edinburgh, UK; z Department of Paediatrics and Child Health, University College Cork, Cork, Ireland; aa London School of Hygiene and Tropical Medicine, London, UK; ab Great Ormond Street Hospital for Children NHS Foundation Trust, London, UK; ac King's Health Partners, King's College London, London, UK

## Abstract

**Background:**

Individuals with rare kidney diseases account for 5–10% of people with chronic kidney disease, but constitute more than 25% of patients receiving kidney replacement therapy. The National Registry of Rare Kidney Diseases (RaDaR) gathers longitudinal data from patients with these conditions, which we used to study disease progression and outcomes of death and kidney failure.

**Methods:**

People aged 0–96 years living with 28 types of rare kidney diseases were recruited from 108 UK renal care facilities. The primary outcomes were cumulative incidence of mortality and kidney failure in individuals with rare kidney diseases, which were calculated and compared with that of unselected patients with chronic kidney disease. Cumulative incidence and Kaplan–Meier survival estimates were calculated for the following outcomes: median age at kidney failure; median age at death; time from start of dialysis to death; and time from diagnosis to estimated glomerular filtration rate (eGFR) thresholds, allowing calculation of time from last eGFR of 75 mL/min per 1·73 m^2^ or more to first eGFR of less than 30 mL/min per 1·73 m^2^ (the therapeutic trial window).

**Findings:**

Between Jan 18, 2010, and July 25, 2022, 27 285 participants were recruited to RaDaR. Median follow-up time from diagnosis was 9·6 years (IQR 5·9–16·7). RaDaR participants had significantly higher 5-year cumulative incidence of kidney failure than 2·81 million UK patients with all-cause chronic kidney disease (28% *vs* 1%; p<0·0001), but better survival rates (standardised mortality ratio 0·42 [95% CI 0·32–0·52]; p<0·0001). Median age at kidney failure, median age at death, time from start of dialysis to death, time from diagnosis to eGFR thresholds, and therapeutic trial window all varied substantially between rare diseases.

**Interpretation:**

Patients with rare kidney diseases differ from the general population of individuals with chronic kidney disease: they have higher 5-year rates of kidney failure but higher survival than other patients with chronic kidney disease stages 3–5, and so are over-represented in the cohort of patients requiring kidney replacement therapy. Addressing unmet therapeutic need for patients with rare kidney diseases could have a large beneficial effect on long-term kidney replacement therapy demand.

**Funding:**

RaDaR is funded by the Medical Research Council, Kidney Research UK, Kidney Care UK, and the Polycystic Kidney Disease Charity.

## Introduction

Chronic kidney disease is an umbrella term for conditions resulting in impaired kidney function, and can be divided into five stages defined by estimated glomerular filtration rate (eGFR). Chronic kidney disease stages 3, 4, and 5 represent moderate to severe disease and affect an estimated 6·1% of the UK population over the age of 16 years and 32·7% of those older than 75 years.^1^ The most common causes of chronic kidney disease stage 3 in high-income and middle-income countries are diabetes and hypertension.^2^

Rare diseases are generally defined as affecting fewer than 200 000 individuals in the USA,^3^ or fewer than five per 10 000 individuals in Europe.^4^ Approximately 80% of rare diseases are inherited.^5^ Rare kidney diseases, as defined by the Kidney Disease: Improving Global Outcomes global organisation, include more than 150 conditions^6^ and have an estimated prevalence of 60–80 cases per 100 000 people in Europe and the USA.^5^

More than 50% of children and those younger than 20 years receiving kidney replacement therapy have a rare kidney disease.^7^ In contrast to earlier chronic kidney disease stages, glomerulonephritis (which comprises multiple individually rare disorders) accounts for more UK adults receiving kidney replacement therapy than do common causes of chronic kidney disease, such as diabetes.^8^ The reason for this discrepancy is unclear, but it could be explained by a slower average rate of kidney function loss^9^ and higher competing risk of death in unselected individuals with chronic kidney disease.^10^, ^11^ Moreover, although population-based data show differences between males and females in kidney function decline,^12^ little is known about sex differences in renal outcomes for rare kidney diseases, or how kidney failure risk prediction tools that were validated in unselected chronic kidney disease populations (such as the kidney failure risk equation)^13^ perform for patients with rare kidney diseases. A better understanding of disease progression and of renal and patient survival is therefore needed, and could enable more accurate prognostication, inform health-care resource planning, and help to identify optimal opportunities for novel therapeutic interventions to slow progression to kidney failure, potentially reducing the population burden of kidney failure. Novel therapies for rare kidney diseases, such as the recently approved targeted-released budesonide for IgA nephropathy,^14^ are emerging rapidly, and real-world data on historical and current outcomes for rare kidney diseases are needed to appraise safety and efficacy data from clinical trials and inform economic modelling to assess the cost-effectiveness of interventions.

**Research in context**

**Evidence before this study**

Chronic kidney disease is a major global health challenge that can progress to kidney failure. Despite representing less than 10% of the chronic kidney disease population, individuals with rare kidney diseases account for a higher proportion of adults and children receiving kidney replacement therapy in the UK and Europe than do patients with common causes of chronic kidney disease, such as diabetes and hypertension. The natural histories of most rare kidney diseases are poorly characterised, and the reasons for which individuals with rare kidney diseases are over-represented in the kidney failure population are not well understood. Prospective, longitudinal patient registries, especially multicohort registries, which allow the study of these conditions both individually and as a group, are vital tools to better understand rare kidney diseases. We searched PubMed for articles published from database inception to Aug 31, 2023 with search terms “rare renal/kidney disease registry”, “rare renal/kidney disease outcomes”, and “rare renal/kidney disease natural history” in various combinations with no language restrictions. We found a baseline report from one European multicohort rare kidney disease registry. We found no longitudinal studies presenting renal and patient outcomes for multiple rare kidney diseases collectively, and no studies comparing outcomes of people living with rare kidney diseases with the general population, or those with unselected chronic kidney disease.

**Added value of this study**

We used data from the largest multicohort national rare kidney disease registry in the world to provide estimates of renal function decline, kidney failure, and death risk in more than 27 000 patients with 28 rare kidney diseases. These data allow comparison of the presentation, progression, and survival of patients living with different rare kidney diseases, and the comparison between renal and patient outcomes in patients with rare kidney diseases and the general population and those with all-cause chronic kidney disease. For each rare disease, we calculated the time in therapeutic trial window, an estimate of time from diagnosis that patients would have sufficient renal function to participate in a standard therapeutic trial.

**Implications of all the available evidence**

This work illustrates the power of a coordinated rare disease registry. Data on individual rare kidney diseases will be beneficial for patients and clinicians to better inform shared decision making, for regulators and health-care providers to inform licencing decisions and care provision, and for researchers to inform clinical study design. We found that patients with rare kidney diseases have higher rates of kidney failure but longer survival than unselected patients with chronic kidney disease, and are therefore disproportionately represented in the population of patients with kidney failure. Our findings challenge the historical perception that common diseases primarily underpin the occurrence of kidney failure and suggest that successfully addressing unmet need for effective therapies in rare kidney diseases could have a large effect on reducing long-term kidney replacement therapy demand and subsequently result in economic benefits for health-care systems.

In 2010, the UK Kidney Association launched a strategy for rare kidney disorders that included establishing the National Registry of Rare Kidney Diseases (RaDaR). At the time of writing, RaDaR has recruited more than 30 000 patients across 29 disease categories, has links to hospital laboratories and to the UK Renal Registry for validated, clinically important endpoint data for kidney replacement therapy initiation and death, and is, as far as we are aware, the largest rare kidney disease registry in the world.^15^ We used RaDaR data to describe the clinical demographics, disease characteristics, and kidney and patient outcomes for individuals with rare kidney diseases in the UK.

## Methods

### Study design and participants

Data for this retrospective cohort study were extracted from RaDaR on July 25, 2022. Follow-up time was calculated as time from date of diagnosis until July 25, 2022, or death, whichever occurred first.

RaDaR is linked with the UK Renal Registry and receives an automated feed of clinical laboratory results from UK hospitals, including data provided by NHS Blood and Transplant, via the UK Renal Data Collaboration. The UK Renal Registry, UK Renal Data Collaboration, and RaDaR are operated and controlled by the UK Kidney Association. RaDaR data are enriched with UK Renal Registry kidney replacement therapy initiation data, which are known to be complete to Jan 1, 2022. Detailed cross-sectional analysis of epidemiology and potential recruitment biases in RaDaR are presented in a preprinted companion manuscript that does not identify evidence of systematic recruitment biases in ethnicity, socioeconomic status, and sex of RaDaR participants.^16^

This report adheres to STROBE guidelines.^17^ Ethical approval was provided by NHS South West—Central Bristol Research Ethics Committee (14/SW/1088) and by the RaDaR and UK Renal Registry operational committees. RaDaR is a national registry that records patients in 39 rare disease groups (which can be either a single disease or group of kidney diagnoses), from 108 UK kidney clinics. Recruitment began on Jan 18, 2010, and age at recruitment ranged from 0 to 96 years.

Eligibility criteria are available online. and in the appendix (pp 27–50). Participating recruitment centres are detailed in the appendix (p 26). Patients provided written, informed consent at recruitment.

Data for 28 rare disease groups with more than 85 patients recruited are presented: autosomal dominant polycystic kidney disease; autosomal dominant tubulointerstitial kidney disease; atypical haemolytic uraemic syndrome; male X-linked Alport syndrome; female X-linked Alport syndrome; thin basement membrane nephropathy (autosomal recessive Alport syndrome was excluded due to insufficient sample size); autosomal recessive polycystic kidney disease and nephronophthisis; *HNF1B* mutations; proteinuric IgA nephropathy; steroid sensitive nephrotic syndrome or minimal change disease; steroid resistant nephrotic syndrome, congenital nephrotic syndrome, or focal segmental glomerulosclerosis (SRNS–FSGS); membranous nephropathy; monoclonal gammopathy of renal significance; membranoproliferative glomerulonephritis and C3 glomerulopathy (MPGN–C3G) inherited renal cancers; retroperitoneal fibrosis; Shiga toxin or verotoxin-producing *Escherichia coli* associated haemolytic uraemic syndrome; tuberous sclerosis complex; anti-neutrophil cytoplasmic antibody-associated vasculitis; other vasculitis; anti-glomerular basement membrane antibody disease; cystinosis; cystinuria; hyperoxaluria; Gitelman syndrome; Bartter syndrome; Dent disease and Lowe syndrome; and other tubulopathies. The ten other rare disease groups excluded owing either to small sample size or the fact they are not primary rare disease diagnoses are shown in the appendix (p 2).

### Outcomes

The exposure of interest was a diagnosis of rare kidney disease. The primary outcomes were death (ascertained from the UK Renal Registry and Office of National Statistics data) and kidney failure (defined as need for chronic kidney replacement therapy [dialysis or transplantation], or an eGFR of less than 15 mL/min per 1·73 m^2^ for 4 weeks or more). The following were secondary outcomes: median age at kidney failure; median age at death; time from start of dialysis to death; and time from diagnosis to eGFR thresholds, allowing calculation of time from last eGFR of 75 mL/min per 1·73 m^2^ or more to first eGFR of less than 30 mL/min per 1·73 m^2^ (the therapeutic trial window).

eGFR was calculated from patient creatinine results with the Chronic Kidney Disease Epidemiology Collaboration creatinine equation without adjustment for race,^18^ or with the Schwartz equation for those aged 16 years or younger. eGFR at diagnosis was calculated as the mean of values between 6 months before and 3 months after diagnosis. Laboratory results were uploaded from participants’ clinical records; frequency of tests varied between participants, diseases, and centres. For each rare disease group, median number of creatinine measurements per person is shown in the appendix (p 3). Sex is reported as per UK Renal Data Collaboration record.

Estimates of UK chronic kidney disease prevalence were derived from the National CKD Audit^19^ and Quality Improvement in CKD study,^20^, ^21^ and kidney replacement therapy incidence and survival data from the UK Renal Registry annual report.^22^ Survival on kidney replacement therapy for RaDaR patients was compared with UK Renal Registry patients with a primary renal diagnosis of diabetes, hypertension, or renovascular disease.

### Statistical analysis

Exclusion criteria for analyses performed are included in the appendix (p 2). Categorical data were reported as frequencies (%) and continuous data were reported as medians (IQR). *t*-tests for differences between groups were used when the distribution or number of observations allowed, and Mann–Whitney tests otherwise. Fit with the kidney failure risk equation was tested with the Hosmer–Lemeshow test. A significance threshold of 0·05 was used for p values.

Cumulative incidence analyses of time to kidney failure were performed with patients censored at death, and sensitivity analyses with death as a competing risk.^23^ Median age at kidney failure (censored for death) and median age at death were estimated by Kaplan–Meier analysis using age as the time scale, and survival on kidney replacement therapy was estimated using Kaplan-Meier analysis on years from kidney replacement therapy start to death. Because transplantation rates differ between groups and survival rates differ between those receiving dialysis or kidney transplantation,^24^ we performed sensitivity analyses censoring for transplantation. To examine differences between male and female patients, analyses were performed by rare disease group and sex. The proportional hazards assumption was assessed by visual inspection.

For kidney function decline, each patient's individual eGFR trajectory was modelled using a smoothed spline to estimate a continuous curve from their eGFR values. Multiple Kaplan-Meier analyses were used to estimate median time from diagnosis to each multiple of five from an eGFR of 90 to 15 mL/min per 1·73 m^2^ for these continuous curves and plotted to give a visualisation of estimated median eGFR decline by rare disease group. For each multiple of five, if a patient's estimated eGFR curve was below that threshold at diagnosis, then their time value in that time-to-event analysis was zero.

We estimated time in therapeutic trial window (defined as the time between last eGFR ≥75 mL/min per 1·73 m^2^ and first eGFR <30 mL/min per 1·73 m^2^ with no subsequent higher eGFR values) for each patient using the same methods as described previously for eGFR decline, but using the estimated date of eGFR at 75 mL/min per 1·73 m^2^ as the starting point for the time-to-event analysis, and estimated date of eGFR at 30 mL/min per 1·73 m^2^ as the end date. If a patient's smoothed spline was never >75 mL/min per 1·73 m^2^, then their diagnosis date was used as the start point.

2-year risk of kidney failure at date of RaDaR recruitment was calculated using the four-variable kidney failure risk equation recalibrated to a UK primary care population^13^ and compared with observed kidney failures to determine accuracy of the kidney failure risk equation in a rare kidney disease population.

Mortality data from the Office of National Statistics^25^, ^26^, ^27^ were used to indirectly age-sex standardise the RaDaR population from England and Wales to a general English and Welsh population. Adjusted hazard ratios for all-cause death from a large population-based study^11^ were then applied to this standardised population to adjust for eGFR values at recruitment and estimate a standardised mortality ratio with all-cause chronic kidney disease as the reference population.^21^ Analyses were performed using STATA Release 17 and SAS version 9.4.

### Role of the funding source

The study funders had no role in study design, data collection, analysis, interpretation, or writing of the report.

## Results

**Figure 1:**
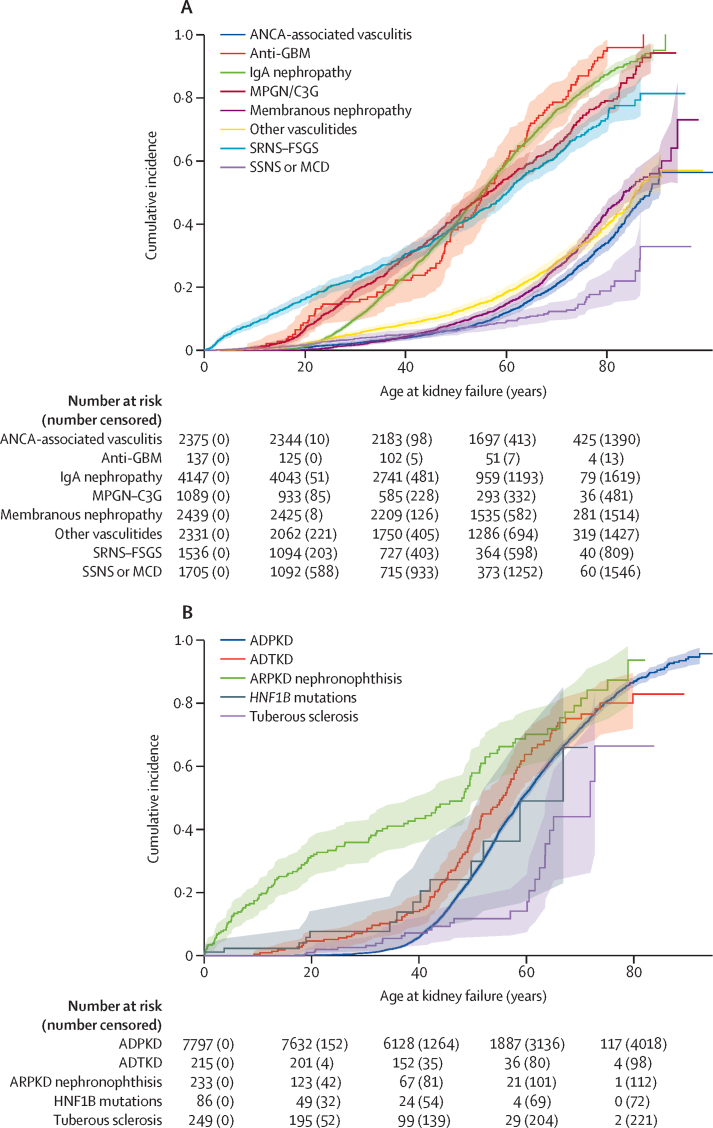
Kaplan-Meier estimates of cumulative incidence of kidney failure for glomerular (A) and cystic kidney diseases (B) Data for all diseases are shown in the appendix (p 4). Data are censored for death. ADPKD=autosomal dominant polycystic kidney disease. ADTKD=autosomal dominant tubulointerstitial kidney disease. ANCA=antineutrophil cytoplasmic antibody. eGFR=estimated glomerular filtration rate. MPGN–C3G=membranoproliferative glomerulonephritis and C3 glomerulopathy. SRNS–FSGS=steroid resistant nephrotic syndrome, congenital nephrotic syndrome, or focal segmental glomerulosclerosis. SSNS–MCD=steroid sensitive nephrotic syndrome or minimal change disease.

27 285 patients in 28 rare disease groups, recruited between Jan 18, 2010, and July 25, 2022, were included. Median age at diagnosis for the whole RaDaR population was 40·6 years (IQR 23·7–57·1) and varied by rare disease group (table 1). Median follow-up was 9·6 years (IQR 5·9–16·7; table 2). There was substantial heterogeneity in age at kidney failure between rare disease groups (figure 1; table 2; appendix p 4). For example, individuals with cystinosis (table 2) frequently reached kidney failure in childhood (median age at kidney failure 15·4 years [IQR 11·6–19·9]), whereas individuals with vasculitis, *HNF1B* mutations, thin basement membrane nephropathy, monoclonal gammopathy of renal significance, and membranous nephropathy typically reached kidney failure aged 65 years and older. Sensitivity analyses for age at kidney failure and time from diagnosis with death as a competing risk resulted in similar findings (appendix pp 5–6).

**Table 1:** Age, sex, and ethnicity of participants and deaths, kidney failure events, and date of first recruitment for each rare disease group

	**Age at recruitment, years**			**Sex, n (%)**		**Ethnicity, n (%)**			**Kidney failure events, n (%)**	**Deaths, n (%)**	**First recruitment date**
	Median (IQR)	Min	Max	Male	Female	White	Non-White	Missing			
Autosomal dominant polycystic kidney disease	52 (41–62)	0	93	3738 (48%)	4059 (52%)	5801 (74%)	497 (6%)	1499 (19%)	3699 (47%)	685 (9%)	Nov 2015
Autosomal dominant tubulointerstitial kidney disease	52 (39–62)	9	88	90 (42%)	125 (58%)	154 (72%)	11 (5%)	50 (23%)	113 (53%)	24 (11%)	May 2013
X-linked Alport syndrome-female	38 (20–53)	1	79	0	309 (100%)	207 (67%)	22 (7%)	80 (26%)	93 (30%)	13 (4%)	Apr 2013
X-linked Alport syndrome-male	36 (21–51)	1	86	412 (100%)	0	265 (64%)	30 (7%)	117 (28%)	270 (66%)	27 (7%)	May 2013
Thin basement membrane nephropathy	39 (22–52)	2	74	54 (33%)	11 (67%)	105 (64%)	17 (10%)	43 (26%)	27 (16%)	3 (2%)	Sep 2013
Autosomal recessive polycystic kidney disease nephronophthisis	23 (9–46)	0	83	114 (49%)	119 (51%)	158 (68%)	29 (12%)	46 (20%)	120 (52%)	18 (8%)	Oct 2012
Cystinosis	18 (9–27)	1	70	75 (49%)	78 (51%)	75 (49%)	31 (20%)	47 (31%)	98 (64%)	9 (6%)	Jan 2013
Cystinuria	42 (27–56)	0	82	251 (53%)	222 (47%)	224 (47%)	15 (3%)	234 (49%)	12 (3%)	13 (3%)	Feb 2014
Hyperoxaluria	25 (12–45)	0	82	78 (63%)	46 (37%)	57 (46%)	33 (27%)	34 (27%)	40 (32%)	9 (7%)	Dec 2012
*HNF1B* mutations	19 (8–38)	0	67	47 (55%)	39 (45%)	57 (66%)	8 (9%)	21 (24%)	14 (16%)	1 (1%)	May 2013
Renal cancer inherited	50 (33–59)	1	79	46 (40%)	68 (60%)	42 (37%)	<7 (NA)	70 (61%)	2 (2%)	1 (1%)	Nov 2020
Other tubulopathies	23 (14–44)	0	73	58 (50%)	59 (50%)	55 (47%)	28 (24%)	34 (29%)	11 (9%)	0	Mar 2011
Bartter syndrome	18 (5–37)	0	81	37 (69%)	17 (31%)	24 (44%)	13 (24%)	17 (31%)	3 (6%)	2 (4%)	Oct 2013
Gitelman syndrome	38 (28–53)	6	84	62 (33%)	124 (67%)	137 (74%)	12 (6%)	37 (20%)	1 (1%)	7 (4%)	Oct 2012
Dent disease and Lowe syndrome	15 (8–33)	0	72	61 (98%)	1 (2%)	35 (56%)	<7 (NA)	21 (34%)	16 (26%)	3 (5%)	Feb 2014
Tuberous sclerosis complex	31 (19–47)	2	81	102 (41%)	147 (59%)	170 (68%)	23 (9%)	56 (22%)	26 (10%)	7 (3%)	Mar 2016
Atypical hemolytic uremic syndrome	29 (11–47)	0	79	133 (45%)	160 (55%)	213 (73%)	16 (5%)	64 (22%)	141 (48%)	17 (6%)	Jun 2013
Steroid sensitive nephrotic syndrome or minimal change disease	30 (10–53)	1	93	1004 (59%)	701 (41%)	1060 (62%)	347 (20%)	298 (17%)	104 (6%)	53 (3%)	Apr 2010
SRNS–FSGS	39 (14–59)	0	90	872 (57%)	664 (43%)	1049 (68%)	328 (21%)	159 (10%)	696 (45%)	126 (8%)	Jan 2010
IgA nephropathy	50 (38–61)	4	89	2924 (71%)	1223 (29%)	3219 (78%)	515 (12%)	413 (10%)	2474 (60%)	351 (8%)	Oct 2014
Membranous nephropathy	63 (53–71)	5	95	1634 (67%)	805 (33%)	1806 (74%)	315 (13%)	318 (13%)	681 (28%)	384 (16%)	Oct 2013
MGRS	66 (57–75)	24	91	96 (53%)	85 (47%)	133 (73%)	24 (13%)	24 (13%)	102 (56%)	37 (20%)	Jul 2018
MPGN–C3G	51 (29–64)	1	96	581 (53%)	508 (47%)	800 (73%)	109 (10%)	180 (17%)	588 (54%)	157 (14%)	Jan 2010
Retroperitoneal fibrosis	64 (56–72)	29	88	94 (66%)	48 (34%)	97 (68%)	21 (15%)	24 (17%)	25 (18%)	31 (22%)	May 2016
STEC-associated HUS	9 (5–15)	1	77	83 (49%)	87 (51%)	95 (56%)	<7 (NA)	69 (41%)	32 (19%)	3 (2%)	Jan 2013
ANCA-associated vasculitis	67 (56–74)	1	96	1278 (54%)	1097 (46%)	1856 (78%)	142 (6%)	377 (16%)	628 (26%)	451 (19%)	Sep 2013
Anti-GBM disease	61 (49–69)	10	90	68 (50%)	69 (50%)	114 (83%)	7 (5%)	16 (12%)	121 (88%)	21 (15%)	Oct 2015
Other vasculitides	62 (42–72)	0	96	1187 (51%)	1144 (49%)	1842 (79%)	127 (5%)	362 (16%)	628 (27%)	374 (16%)	Sep 2011

Rare disease group names are those used in RaDaR. ANCA=antineutrophil cytoplasmic antibodies. GBM=glomerular basement membrane. HUS=haemolytic uraemic syndrome. MGRS=monoclonal gammopathy of renal significance. MPGN–C3G=membranoproliferative glomerulonephritis and C3 glomerulopathy. NA=not applicable. RaDaR=the National Registry of Rare Kidney Diseases. SRNS–FSGS=steroid resistant nephrotic syndrome, congenital nephrotic syndrome, or focal segmental glomerulosclerosis. STEC=Shiga toxin or verotoxin producing *Escherichia coli*.

**Table 2:** Age at diagnosis, follow-up, time from diagnosis to kidney failure and death, 10-year renal and patient survival, median eGFR at diagnosis, and time from last eGFR ≥75 mL/min per 1·73 m^2^ to first eGFR ≤30 mL/min per 1·73 m^2^ (time in therapeutic trial window), stratified by rare disease group

		**n (%)**	**Median age at diagnosis (IQR), years**	**Median follow up (IQR), years**	**Median time from diagnosis to kidney failure (95% CI), years**	**Median time from diagnosis to death (95% CI), years**	10-year renal survival (95% CI)*	**10-year patient survival (95% CI)**†	**Median eGFR at diagnosis, mL/min per 1·73 m^2^ (IQR)**	**Median time in therapeutic trial window (95% CI), years**	**Median age at kidney failure (IQR), years**
All RaDaR		27 285 (100%)	40·6 (23·7–57·1)	9·6 (5·9–16·7)	..	..	..	..	..	..	68 (50–88)
Autosomal dominant polycystic kidney disease		7797 (28·6%)	38·3 (26·7–49·8)	13·7 (7·5–22·6)	22·6 (21·9–23·8)	72·9 (63·4–NE)	0·76 (0·74–0·77)	0·97 (0·97–0·98)	66·0 (34·4–93·9)	11·0 (10·7–11·7)	59 (50–72)
Autosomal dominant tubulointerstitial kidney disease		215 (0·8%)	42·7 (27·8–54·5)	9·1 (5·7–15·6)	16·6 (10·4–27·6)	51·0 (34·8–NE)	0·62 (0·52–0·70)	0·95 (0·90–0·98)	44·3 (25·6–66·2)	4·8 (1·8–8·3)	55 (45–67)
X–linked Alport syndrome											
	Female	309 (1·1%)	26·4 (7·6–40·2)	9·6 (5·7–18·6)	40·3 (23·8–52·7)	NE (44·4–NE)	0·81 (0·74–0·86)	0·99 (0·97–1·00)	84·9 (54·6–116·9)	8·6 (4·9–9·9)	64 (42–75)
	Male	412 (1·5%)	19·5 (8·2–34·5)	11·4 (6·7–24·9)	15·1 (12·9–17·2)	NE (50·1–NE)	0·65 (0·58–0·71)	0·97 (0·94–0·99)	67·7 (23·9–114·9)	7·0 (4·9–10·4)	29 (21–46)
Thin basement membrane nephropathy		165 (0·6%)	31·7 (18·1–45·5)	8·0 (5·6–15·0)	33·3 (25·9–NE)	..	0·91 (0·82–0·95)	1·00 (1·00–1·00)	97·4 (69·1–111·2)	12·3 (6·2–17·9)	71 (63–77)
Autosomal recessive polycystic kidney disease nephronophthisis		233 (0·9%)	8·7 (0·1–29·9)	10·5 (6·6–16·6)	19·3 (11·8–26·7)	50·5 (NE–NE)	0·66 (0·58–0·74)	0·95 (0·90–0·98)	54·7 (31·8–81·2)	14·9 (7·8–17·1)	48 (14–66)
Cystinosis		153 (0·6%)	1·9 (0·7–9·9)	13·6 (7·2–23·0)	14·7 (13·0–16·1)	40·8 (37·8–NE)	0·71 (0·61–0·79)	0·99 (0·94–1·00)	63·3 (33·1–91·4)	8·2 (5·5–12·5)	15 (12–20)
Cystinuria		473 (1·7%)	31·6 (18·6–48·5)	8·1 (6·7–14·8)	..	NE (53·8–NE)	1·00 (0·98–1·00)	0·97 (0·95–0·99)	79·7 (67·3–99·8)	LQ 22·9 (17·4–NE)	..
Hyperoxaluria		124 (0·5%)	18·4 (4·5–36·3)	9·4 (7·1–17·2)	NE (42·0–NE)	NE (30·3–NE)	0·84 (0·73–0·90)	0·97 (0·88–0·99)	93·8 (46·6–110·0)	NE	53 (31–NE)
*HNF1B* mutations		86 (0·3%)	13·1 (2·1–34·5)	8·3 (6·0–12·3)	22·3 (16·4–NE)	..	0·95 (0·85–0·98)	0·98 (0·89–1·00)	45·3 (26·2–69·9)	16·2 (1·3–NE)	67 (50–NE)
Renal cancer inherited		114 (0·4%)	42·5 (26·0–53·8)	6·0 (3·9–10·4)	..	NE (17·2–NE)	0·98 (0·93–1·00)	1·00 (1·00–1·00)	..	NE	..
Other tubulopathies		117 (0·4%)	15·5 (6·9–41·9)	9·7 (4·4–15·1)	NE (12·8–NE)	..	0·85 (0·64–0·94)	1·00 (1·00–1·00)	..	NE	..
Bartter syndrome		54 (0·2%)	4·7 (0·3–24·4)	12·1 (5·7–18·6)	..	..	0·97 (0·83–1·00)	0·95 (0·82–0·99)	..	NE	..
Gitelman syndrome		186 (0·7%)	32·8 (21·2–44·5)	9·9 (6·7–14·2)	..	..	1·00 (1·00–1·00)	0·98 (0·94–0·99)	106·3 (93·1–120·5)	NE	..
Dent disease and Lowe syndrome		62 (0·2%)	9·4 (1·0–29·0)	10·0 (6·9–14·6)	..	..	0·79 (0·62–0·88)	0·96 (0·83–0·99)	..	16·9 (0·0–NE)	55 (36–66)
Tuberous sclerosis complex		249 (0·9%)	15·4 (2·4–31·6)	12·7 (6·3–22·6)	61·0 (NE–NE)	58·1 (58·1–NE)	0·97 (0·93–0·99)	1·00 (1·00–1·00)	95·4 (63·2–108·8)	11·2 (5·8–NE)	72 (62–NE)
Atypical HUS		293 (1·1%)	19·9 (3·4–40·0)	7·9 (4·7–12·1)	21·7 (17·5–26·6)	NE (36·2–NE)	0·71 (0·63–0·78)	0·94 (0·89–0·97)	28·5 (12·9–70·5)	1·8 (0–NE)	41 (24–62)
Steroid sensitive nephrotic syndrome or minimal change disease		1705 (6·2%)	16·8 (4·1–46·5)	8·5 (5·3–13·6)	54·3 (52·9–NE)	NE (54·1–NE)	0·95 (0·94–0·96)	0·97 (0·96–0·98)	91·5 (64·9–118·1)	LQ 8·5 (6·8–13·4)	(86–NE)
SRNS–FSGS		1536 (5·6%)	27·7 (7·0–50·6)	10·6 (6·4–16·5)	16·5 (14·1–19·0)	..	0·62 (0·59–0·64)	0·94 (0·93–0·95)	77·0 (40·9–111·6)	4·6 (3·8–5·6)	60 (33–80)
IgA nephropathy		4147 (15·2%)	40·4 (29·5–51·9)	9·8 (6·2–16·7)	10·7 (10·1–11·7)	..	0·52 (0·50–0·54)	0·95 (0·94–0·96)	40·0 (23·1–67·8)	4·0 (3·7–4·4)	55 (41–69)
Membranous nephropathy		2439 (8·9%)	56·8 (45·4–67·2)	8·1 (5·1–13·5)	28·6 (22·4–32·3)	42·1 (31·3–48·0)	0·77 (0·74–0·79)	0·87 (0·85–0·89)	67·4 (43·6–90·3)	11·2 (9·5–12·5)	83 (69–NE)
MGRS		181 (0·7%)	62·7 (51·8–72·1)	5·5 (3·6–8·7)	8·2 (4·9–11·9)	16·0 (13·7–19·6)	0·43 (0·31–0·54)	0·78 (0·66–0·86)	34·1 (14·5–58·8)	1·3 (0·2–3·6)	72 (60–85)
MPGN–C3G		1089 (4·0%)	34·1 (15·2–55·6)	9·9 (6·5–17·8)	17·6 (15·5–22·1)	49·2 (42·5–NE)	0·67 (0·63–0·70)	0·91 (0·88–0·92)	63·0 (31·8–95·3)	6·3 (4·8–8·0)	56 (36–76)
Retroperitoneal fibrosis		142 (0·5%)	56·9 (51·5–66·8)	8·6 (5·2–12·7)	NE (20·5–NE)	22·0 (16·3–NE)	0·82 (0·71–0·89)	0·84 (0·75–0·91)	42·9 (31·4–66·4)	20·0 (13·4–NE)	NE (74– NE)
STEC-associated HUS		170 (0·6%)	3·4 (1·7–7·0)	8·8 (6·3–13·0)	22·9 (17·6–NE)	..	0·91 (0·84–0·95)	0·99 (0·94–1·00)	..	13·0 (0·0–NE)	36 (23–64)
ANCA-associated vasculitis		2375 (8·7%)	61·9 (50·4–70·5)	7·7 (4·7–12·6)	34·9 (27·9–60·4)	29·8 (25·1–36·0)	0·79 (0·77–0·81)	0·82 (0·80–0·84)	30·5 (18·6–51·0)	10·5 (8·7–14·0)	89 (73– NE)
Anti-GBM disease		137 (0·5%)	54·9 (36·0–64·2)	7·6 (4·9–14·8)	0·1 (0·1–0·3)	NE (28·0–NE)	0·17 (0·09–0·27)	0·83 (0·74–0·90)	11·2 (6·7–17·5)	NE	56 (44–67)
Other vasculitides		2331 (8·5%)	52·2 (24·0–66·6)	7·7 (4·8–12·6)	28·0 (25·4–35·1)	41·8 (34·9–NE)	0·79 (0·77–0·82)	0·85 (0·83–0·88)	37·5 (18·1–69·6)	12·7 (8·9–15·3)	86 (67– NE)

Rare disease group names are those used in RaDaR. ANCA=antineutrophil cytoplasmic antibodies. GBM=glomerular basement membrane. HUS=haemolytic uraemic syndrome. LQ=lower quartile. MGRS=monoclonal gammopathy of renal significance. MPGN–C3G=membranoproliferative glomerulonephritis and C3 glomerulopathy. NE=not evaluable. RaDaR=the National Registry of Rare Kidney Diseases. SRNS–FSGS=steroid resistant nephrotic syndrome, congenital nephrotic syndrome, or focal segmental glomerulosclerosis. STEC=Shiga toxin and verotoxin producing *Escherichia coli*.

* Years from diagnosis to kidney failure.

† Years from diagnosis to death.

Median age at death was either not evaluable due to too few events or above 75 years for all rare disease groups except cystinosis, for which median age at death was 56·4 years (95% CI 40·9–not evaluable, appendix p 7).

Renal impairment severity at diagnosis differed between rare disease groups (table 2). Predictably, patients with thin basement membrane nephropathy and tuberous sclerosis complex presented with normal kidney function at diagnosis, and patients with atypical haemolytic uraemic syndrome and anti-glomerular basement membrane disease frequently presented with decreased kidney function (median eGFR 29 mL/min per 1·73 m^2^ for atypical haemolytic uraemic syndrome and 11 mL/min per 1·73 m^2^ for anti-glomerular basement membrane) due to acute kidney injury at presentation.

**Figure 2:**
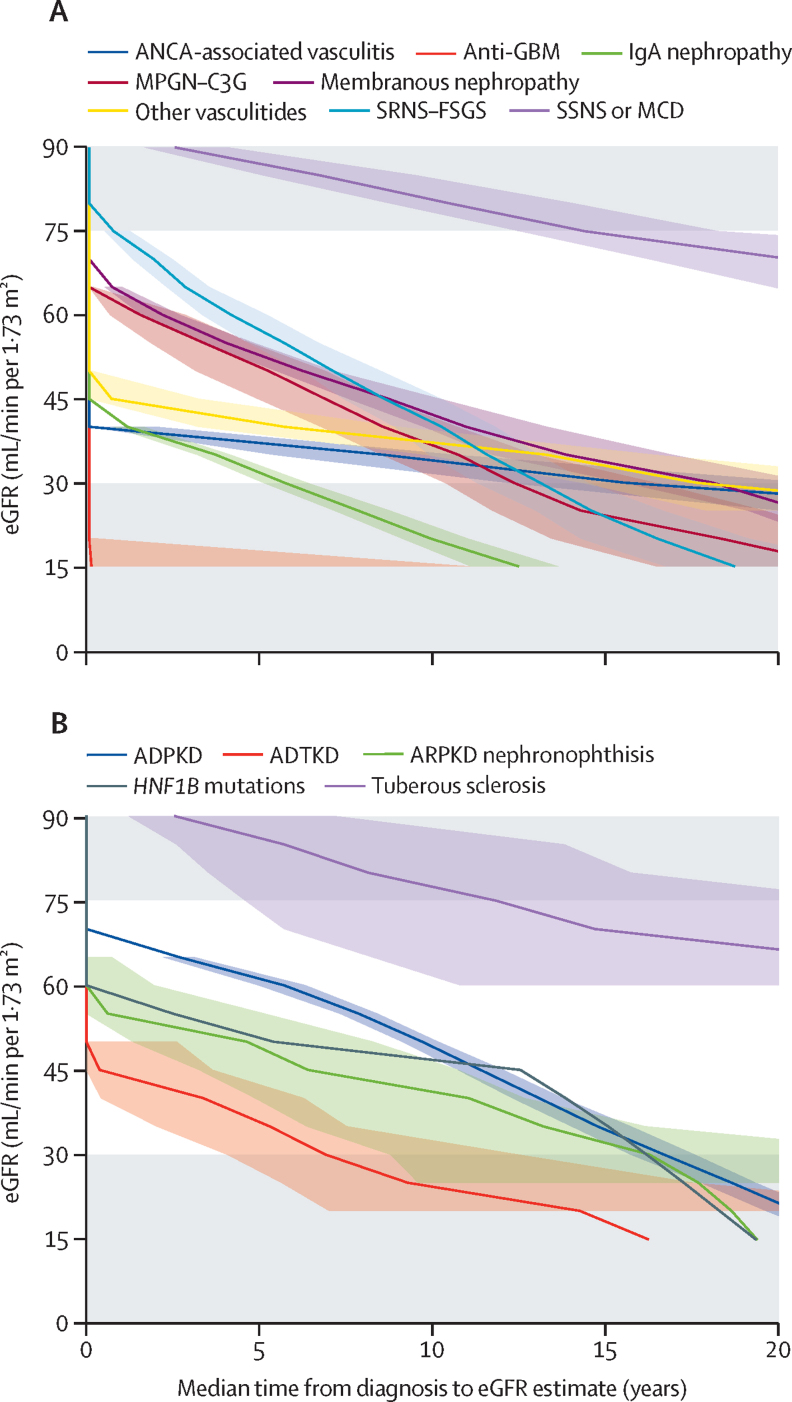
Kaplan-Meier estimates of median time from diagnosis to eGFR value for glomerular (A) and cystic kidney diseases (B) Data for all diseases are shown in the appendix (p 8). ADPKD=autosomal dominant polycystic kidney disease. ADTKD=autosomal dominant tubulointerstitial kidney disease. ANCA=antineutrophil cytoplasmic antibody. eGFR=estimated glomerular filtration rate. GBM=glomerular basement membrane. MPGN–C3G=membranoproliferative glomerulonephritis and C3 glomerulopathy. SRNS–FSGS=steroid resistant nephrotic syndrome, congenital nephrotic syndrome, or focal segmental glomerulosclerosis. SSNS–MCD=steroid sensitive nephrotic syndrome or minimal change disease.

Time in therapeutic trial window ranged from 20·0 years in retroperitoneal fibrosis to 1·3 years in monoclonal gammopathy of renal significance (table 2; appendix p 8). Shorter times could represent a steeper eGFR slope or a lower eGFR at diagnosis. For example, patients with antineutrophil cytoplasmic antibody-associated vasculitis (median eGFR at diagnosis 30·5 mL/min per 1·73 m^2^) and IgA nephropathy (40·0 mL/min per 1·73 m^2^) were diagnosed with similar eGFR; however, time to reach an eGFR of 30 mL/min per 1·73 m^2^ was estimated at 10·5 years for antineutrophil cytoplasmic antibody-associated vasculitis, but only 4·0 years for IgA nephropathy (figure 2).

People with rare kidney diseases recruited to RaDaR had more than twice as many observed deaths within 1 year of recruitment than expected when indirectly standardised to the English and Welsh population (standardised mortality ratio 2·29 [95% CI 2·09–2·49]). This figure increased for patients with kidney failure at or within 1 year of recruitment date (3·99 [3·55–4·42]) and decreased for those without kidney failure (1·32 [1·13–1·51]). However, there were significantly fewer observed deaths than expected when the RaDaR cohort with chronic kidney disease stages 3–5 was indirectly standardised to a population with all-cause chronic kidney disease (0·42 [0·32–0·52]; table 3). RaDaR patients aged 18 years and older receiving dialysis treatments had longer survival than patients in the UK Renal Registry with primary renal diagnoses of diabetes, hypertension, or renovascular disease, stratified by age (logrank 0–17 years p=0·54; p<0·0001 for age groups 18–44, 45–59, 60–69, and ≥70 years; appendix p 9). Sensitivity analyses censoring for kidney transplantation showed similar differences (data not shown).

**Table 3:** Observed and expected deaths with calculated standardised mortality ratios for patients in RaDaR overall, and stratified by severity of kidney disease at recruitment (with or without kidney failure) compared to the general population in England and Wales, and for all-cause chronic kidney disease population

	**Expected deaths**	**Observed deaths**	**Standardised mortality ratio (95% CI)**	**p value**
RaDaR (standardised to general population)*				
Male	145	341	2·35 (2·10–2·60)	<0·0001
Female	76	166	2·17 (1·84–2·50)	<0·0001
Total	221	507	2·29 (2·09–2·49)	<0·0001
**RaDaR patients with kidney failure (standardised to general population)**†				
Male	55	213	3·88 (3·36–4·41)	<0·0001
Female	26	109	4·21 (3·42–5·00)	<0·0001
Total	81	322	3·99 (3·55–4·42)	<0·0001
**RaDaR excluding those with kidney failure (standardised to general population)**				
Male	90	128	1·42 (1·18–1·67)	0·0004
Female	51	57	1·13 (0·83–1·42)	0·20
Total	141	185	1·32 (1·13–1·51)	0·0005
**RaDaR patients with CKD stages 3–5 (standardised to all-cause UK chronic kidney disease population)**‡				
Male	109	45	0·41 (0·29–0·53)	<0·0001
Female	57	25	0·44 (0·27–0·61)	<0·0001
Total	166	70	0·42 (0·32–0·52)	<0·0001

RaDaR=the National Registry of Rare Kidney Diseases.

* Office of National Statistics data.

† Patients receiving kidney replacement therapy, or with an eGFR ≤15 mL per min per 1·73 m^2^ before 1 year after recruitment date.

‡ Excluding patients without eGFR values at diagnosis.

**Figure 3:**
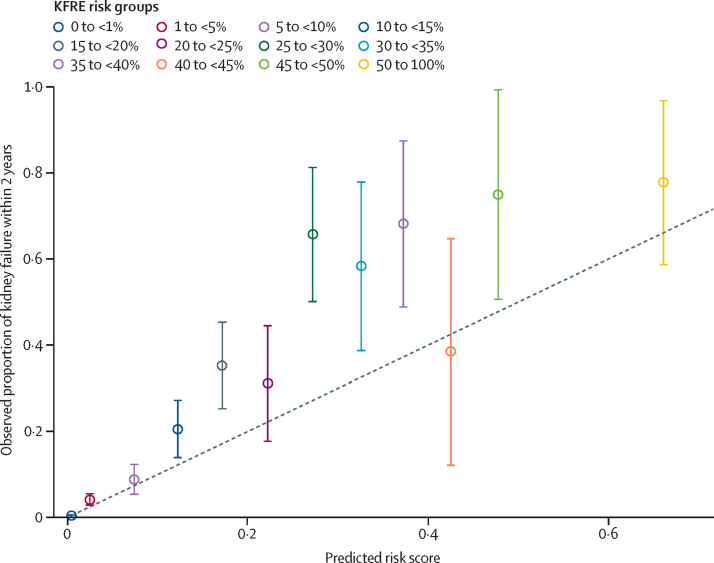
Observed *vs* predicted kidney failure within 2 years in RaDaR patients Patients with eGFR >15 and <60 mL/min per 1·73 m^2^ at recruitment who remained alive with more than 2 years of follow-up data are shown. Observed kidney failure exceeded that predicted by the KFRE (Hosmer-Lemeshow test; p<0·0001). Data stratified by age group are shown in the appendix (p 12). Blue dashed line indicates observed proportion of kidney failure within 2 years equalling the predicted risk score. KFRE=kidney failure risk equation. RaDaR=the National Registry of Rare Kidney Diseases.

RaDaR patients with chronic kidney disease stages 3–5 not receiving kidney replacement therapy at recruitment had markedly higher 1-year, 3-year, and 5-year cumulative incidence of kidney failure compared with UK patients with all-cause chronic kidney disease (5-year cumulative incidence of kidney failure 28% *vs* 1%; appendix p 10). RaDaR patients aged 28 years and older had a higher cumulative incidence of age-stratified kidney replacement therapy than patients with all other causes of kidney failure in the general chronic kidney disease population (appendix p 11). For RaDaR participants younger than 28 years, the incidence of kidney failure was greater than for all disease categories except glomerulonephritis and other (which includes a high proportion of individuals with congenital urinary tract malformations and other individually rare diseases^28^), consistent with established evidence that rare kidney diseases account for the majority of childhood kidney failure.^7^, ^8^ The observed rates of kidney failure in the RaDaR population exceeded the rates predicted by the four-variable kidney failure risk equation^13^ overall (p<0·0001) and for most levels of predicted kidney failure risk (figure 3), although the difference between observed and kidney failure risk equation-predicted rates of kidney failure decreased for patients older than 65 years (appendix p 12), in which group it was not significant (p=0·25).

For X-linked Alport syndrome, compared with female patients, male patients were younger at diagnosis (19 *vs* 26 years; p=0·015), reached kidney failure at a younger age (29 *vs* 64 years; p<0·0001), and had a shorter time from diagnosis to kidney failure (15·1 *vs* 40·3 years; p<0·0001). Median age at diagnosis was higher in male patients than female patients in the following rare disease groups: autosomal dominant polycystic kidney disease (40 *vs* 36 years; p<0·0001), IgA nephropathy (42 *vs* 38 years; p<0·0001), MPGN–C3G (38 *vs* 31 years; p=0·039), and SRNS–FSGS (31 *vs* 23 years; p=0·019). In these groups, eGFR at diagnosis was lower in male patients than female patients (appendix pp 13–14). Although time to kidney failure was lower in male patients than in female patients in these groups (appendix pp 15–19), age at diagnosis was inversely correlated with time to kidney failure in both sexes (appendix p 20) and, in a Cox regression analysis accounting for eGFR at diagnosis, sex differences in time to kidney failure were not significant (data not shown). Furthermore, no significant differences in age at kidney failure by sex were observed (appendix pp 21–24), which is consistent with diagnosis taking place later in disease course in male patients than in female patients in these rare disease groups.

Survival was similar among male and female patients recruited to RaDaR without kidney failure when standardised to men and women in the general population (standardised mortality ratio 1·42 [95% CI 1·18–1·67] for men and 1·13 [0·83–1·42] for women). Both male and female RaDaR participants receiving kidney replacement therapy had better survival compared with individuals with a diagnosis of diabetes, hypertension, or renovascular disease in the UK Renal Registry (appendix p 25). There was no difference in excess deaths between sexes when comparing RaDaR patients with kidney failure with the general population (table 3).

## Discussion

Our analyses describe the natural history of 27 285 patients with rare kidney diseases in the UK and provide reliable estimates for kidney function decline, kidney failure, and death risk for 28 categories of rare kidney diseases. We show that, overall, patients with rare kidney diseases differ substantially from the general population with chronic kidney disease: they have higher 5-year rates of kidney failure, but higher survival than other patients with chronic kidney disease stages 3–5 and longer survival on dialysis, and so are disproportionately represented in the population of patients requiring kidney replacement therapy. Importantly, we also found that the disease trajectory for patients with rare kidney diseases is faster than in unselected individuals with chronic kidney disease and, in those younger than 65 years, is not accurately predicted by the kidney failure risk equation. The heterogeneity of outcomes among the included diseases highlights the clinical utility of making a precise diagnosis: not only does it inform treatment decisions, it also provides key prognostic information and identifies diseases with particular unmet need for better treatments. Our findings are consistent with previously reported data showing that most paediatric patients receiving kidney replacement therapy have a rare kidney disease diagnosis,^7^, ^8^, ^28^ and we also show that adults with rare kidney diseases similarly have a much higher incidence of kidney failure than those with chronic kidney disease attributed to common diseases.

Strengths of this study include the large patient population, validated endpoints, long follow-up, and comparison with other causes of chronic kidney disease. Limitations include the fact that RaDaR reflects the clinical practice, ethnic, and genetic composition of the UK and hence findings might not be generalisable to other settings; recruitment criteria for certain rare disease groups might favour ascertainment of patients with more severe disease (eg, IgA nephropathy included biopsy-confirmed diagnosis, proteinuria >0·5 g/day, or eGFR <60mL/min per 1·73 m^2^ during disease course); and data for age at death are limited by length of follow-up and survivor bias. Additionally, most data are stratified by rare disease group, combining subcategories of patients, diagnoses, and treatments given. Because different rare diseases occur at different ages, comparisons of intervals between diagnosis and kidney failure among different diseases might not always be meaningful: in diseases that predominantly occur in young people, the clinical implications of kidney failure within 10 years of diagnosis are quite different from the implications in diseases that typically occur later in life. Therefore, we present renal survival data in each disease with reference to the age at which the disease occurs and not standardised to the general population. We note that many rare kidney diseases have extrarenal manifestations that can cause substantial morbidity—this is not presented in this analysis, which focuses on kidney and mortality outcomes. We present summary data encompassing renal survival and mortality for the entire RaDaR dataset, and although the size of each RaDaR cohort is closely tied to the relative prevalence of that disorder in the kidney failure population,^16^ the cohorts will have a proportionate impact on these figures due to their varying sizes. Therefore, we recommend that disease-level statistics (table 2) are used where appropriate. Because this is a cohort study with no healthy or unselected chronic kidney disease control groups, indirect comparisons were made with other national-level data sources (eg, Office of National Statistics for the general population, which will of course include some individuals with kidney disease), raising the possibility of biases affecting some of the estimates. The appendix (p 9) shows that, among individuals receiving dialysis, there was minimal evidence of survivor bias for patients younger than 70 years; however, in patients aged 70 years and older, survival among RaDaR participants tended to be better than in those eligible but not recruited.

These data provide real-world insight into the presentation and natural history of individual rare kidney diseases, and can be immediately beneficial to patients, clinicians, health-care planners, regulators, and researchers. Our findings will allow patients to be better informed regarding prognostication for their rare kidney condition, enabling better informed decision making about both therapeutic and life choices. Our findings can also inform clinical study design in identifying diseases and subgroups with greatest unmet need for treatments, informing power calculations to detect effect of a therapeutic intervention, and assessing feasibility (eg, estimating the number of UK patients with defined genetic or clinical characteristics).

These data also provide insight into effectiveness of current treatments and optimal timing within the disease course. For example, patients with cystinosis are diagnosed early (median age 1·9 years [IQR 0·7–9·9]) with preserved kidney function, and median age at kidney failure is now 15·4 years, compared with approximately 9 years^29^ before cysteamine treatment; however, our findings highlight the need for research to identify additional therapies that preserve kidney function more effectively in this disease. Conversely, patients with antineutrophil cytoplasmic antibody-associated vasculitis present with lower eGFR, but kidney function decline in this cohort was slow and might reflect effectiveness of current treatments. 10-year kidney survival in RaDaR (79% survival from 2375 patients) was similar to that of 302 patients with antineutrophil cytoplasmic antibody-associated vasculitis who participated in six European Vasculitis Study Group trials^30^ (and in which kidney survival at 7·1 years was 86%). We also report the novel finding of the short therapeutic trial window for patients with newly diagnosed IgA nephropathy. Studies that are not restricted to newly diagnosed patients and include those with well preserved kidney function diagnosed many years previously might inadvertently select for individuals with unusually mild disease, leading to reduced sensitivity to detect evidence of treatment efficacy. Novel interventions might therefore be best targeted at making the diagnosis earlier, salvaging or protecting kidney function as the disease progresses, or reducing adverse effects of chronic treatments, depending on the course observed in each disease. However, our current crude estimates of progression rates are affected by health inequalities that might also be independently correlated with disease outcomes. Therefore, more detailed analyses of care pathways for each condition will be needed to better define suitable intervention opportunities.

In autosomal dominant polycystic kidney disease, IgA nephropathy, MPGN–C3G, and SRNS–FSGS, we observed younger median age and better eGFR at diagnosis in female patients than in male patients. Time to kidney failure was longer in female patients in these groups, but median age at kidney failure was similar in both sexes. This finding could represent an ascertainment (rather than biological) difference, perhaps resulting from increased interactions with health care in early adulthood among female individuals, suggesting that if early detection strategies for these rare kidney diseases are introduced in the future, they might need to target male individuals.

In conclusion, we used large-scale data to provide reliable estimates of rates of kidney function decline, kidney failure, and death in 28 categories of rare kidney diseases. In unselected cohorts with chronic kidney disease, death before kidney failure is a common adverse outcome,^11^ whereas among patients with rare kidney diseases, survival is higher and kidney failure much more common. This finding means that, although strategies to address cardiovascular risk and other causes of death are very important in the large number of individuals with chronic kidney disease in the population, a substantial proportion of kidney failure is attributable to individually rare kidney diseases in which cardiovascular risk reduction will not prevent kidney failure. Patients with rare kidney diseases should therefore be distinguished from those with more common causes of chronic kidney disease, emphasising the importance of early specialist referral and diagnosis, and the need for rare kidney disease-specific treatments aimed at delaying progression to kidney failure. Successfully addressing the unmet need for treatments to protect the kidneys in rare diseases could have a disproportionately beneficial effect on kidney replacement therapy demand and, consequently, economic benefits for health-care systems.

## Data sharing

The RaDaR database is hosted by the UK Renal Registry and its metadata are available via https://rarerenal.org. Individual-level data are not available for export. Proposals to perform analyses using the data for academic, audit, or commercial purposes can be made to the RaDaR Operations Group via https://rarerenal.org.

## Declaration of interests

ERM reports support for the current manuscript from VHL UK/Ireland and consulting fees from MSD. SM is chair of OxalEurope. MS reports support for the current manuscript from a Medical Research Council UK Precision Medicine programme grant (MR/R013942/1) and consulting fees from Travere Therapeutics. RJC reports support for the current manuscript from Kidney Research UK. JAS reports support for the current manuscript from Kidney Research UK, Northern Counties Kidney Research Fund, and the Medical Research Council UK (all payments to institution). JAS is Academic Vice President of the UK Kidney Association. FWKT reports support from the National Institute for Health and Care Research Imperial Biomedical Centre. DN is the UK Kidney Association Director of Informatics Research. DPG reports support for the current manuscript from St Peter's Trust for Kidney Bladder and Prostate Research, Medical Research Council, Kidney Research UK, Kidney Care UK, and Polycystic Kidney Disease Charity (all payments to institution). DPG chairs the Rare Diseases Committee of the UK Kidney Association and reports fees for consulting and presenting from Novartis, Alexion, Calliditas, Sanofi, Britannia, and Travere. All other authors declare no competing interests.
