# Supplementary material for: Effects of rare kidney diseases on kidney failure: a longitudinal analysis of the UK National Registry of Rare Kidney Diseases (RaDaR) cohort
Source: Lancet. 2024 Mar 30;403(10433):1279–89. doi: 10.1016/S0140-6736(23)02843-X (PMC11750427; doi:10.1016/S0140-6736(23)02843-X)
Supplement: Supplementary appendix [file mmc1.pdf]

THE LANCET

Supplementary appendix

This appendix formed part of the original submission and has been peer reviewed. We post it as supplied by the authors.

Supplement to: Wong K, Pitcher D, Braddon F, et al. Effects of rare kidney diseases on kidney failure: a longitudinal analysis of the UK National Registry of Rare Kidney Diseases (RaDaR) cohort. *Lancet* 2024; published online March 13. https://doi.org/10.1016/S0140-6736(23)02843-X.

Effects of rare kidney diseases on kidney failure: a longitudinal analysis of the UK National Registry of Rare Kidney Diseases (RaDaR) Cohort

Supplementary Materials

Table of Contents

Supplementary Figure 1: Flow diagram of included patients for each analysis 2

Supplementary Table 1: Number of creatinine measurements (taken prior to kidney failure) per person in each rare disease group 3

Supplementary Figure 2: Cumulative incidence plot displaying Kaplan-Meier estimates of Kidney Failure censored for death 4

Supplementary Figure 3: Cumulative incidence of years from diagnosis to KF 5

Supplementary Figure 4: Kaplan Meier Survival analyses of Age at Death 7

Supplementary Figure 5: Estimated eGFR trajectories plotting Kaplan-Meier estimates of median time from diagnosis to eGFR value 8

Supplementary Figure 6: Age stratified Kaplan Meier Survival analyses of years from dialysis start to death (not censored for transplant) 9

Supplementary Table 2: 1-, 3- and 5- year cumulative incidence of Kidney Failure in the RaDaR and UK CKD populations 10

Supplementary Figure 7: Kidney Replacement Therapy incidence 2017-2021 per 100 patients 11

Supplementary Figure 8: Calibration plot for 2-year 4 variable Kidney Failure Risk Equation (KFRE) for RaDaR patients stratified by age group 12

Supplementary Table 3: Median age at diagnosis, by Sex 13

Supplementary Table 4: Median eGFR at diagnosis, by Sex 14

Supplementary Table 5: Time from diagnosis to Kidney Failure and 10-year renal survival, stratified by Rare Disease Group and Sex 15

Supplementary Figure 9: Time from diagnosis to Kidney Failure for Males and Females, by Rare Disease Group* 16

Supplementary Figure 10: Time from diagnosis to Kidney Failure for Males and Females with ADPKD, IgA nephropathy and MPGN/C3G, stratified by age quartile at diagnosis 20

Supplementary Figure 11: Age at Kidney Failure for Males and Females, by RDG* 21

Supplementary Figure 12: Sex stratified Kaplan Meier Survival analyses of years from dialysis start to death (not censored for transplant) 25

Supplementary Table 6: Recruiting centres 26

Supplementary Appendix 1: RaDaR Eligibility Criteria 27

Supplementary Appendix 2: National Registry of Rare Kidney Diseases (RaDaR) Consortium 51

Supplementary Figure 1: Flow diagram of included patients for each analysis

Data extraction 25th July 2022
n=29,166 participants
n= 29,329 diagnoses

Excluded
Unclear Diagnosis Recorded n= 39
Idiopathic Nephrotic Syndrome, Unspecified n= 975
Pregnancy n=692
Rare Disease Group size <85 patients
CKD Africa Genes n=65
BK nephropathy n= 62
Calciphylaxis n= 59
Fibromuscular dysplasia n= 42
Fabry Disease n=47
APRT Deficiency n=9
Mitochondrial renal disease = 4
Pure red cell aplasia n=7
Autosomal Recessive Alport Syndrome n= 34

n= 27,285 participants
n= 27,294 diagnoses*

Age at ESKD and death

Inclusion criteria:
All participants

Excluded:
n=0

n = 27,285 participants
n= 27,294 diagnoses

Time from diagnosis to Kidney Failure

Inclusion criteria:
Participants with diagnosis date available

Participants with date of Kidney Failure after date of diagnosis

Excluded:

Rare Disease Group	n
All	6689
ADPKD	2667
ADTKD	74
AHUS	113
ANCA vasculitis	175
ARPKD NPHP	82
Anti-GBM	49
Bartter	7
Cystinosis	37
Cystinuria	55
Dent Disease and Lowe Syndrome	12
Gitelman	1
HNF1b Mutations	22
Hyperoxaluria	37
IgA Nephropathy	850
MGRS	49
MPGN/C3G	360
Membranous Nephropathy	547
Other Tubulopathies	86
Other Vasculitides	953
Renal Cancer Inherited	1
Retroperitoneal Fibrosis	30
SRNS/FSGS	122
SSNS/MCD	51
STEC-associated HUS	23
TBMN	12
Tuberous Sclerosis	46
X-linked AS-Females	72
X-linked AS-Males	156

n = 20,605 diagnoses

Time from diagnosis to death

Inclusion criteria:
Participants with diagnosis date available

Excluded:

Rare Disease Group	n
All	5097
ADPKD	2381
ADTKD	42
AHUS	57
ANCA vasculitis	5
ARPKD NPHP	54
Anti-GBM	0
Bartter	7
Cystinosis	15
Cystinuria	52
Dent Disease and Lowe Syndrome	10
Gitelman	0
HNF1b Mutations	17
Hyperoxaluria	21
IgA Nephropathy	469
MGRS	25
MPGN/C3G	233
Membranous Nephropathy	454
Other Tubulopathies	85
Other Vasculitides	860
Renal Cancer Inherited	1
Retroperitoneal Fibrosis	27
SRNS/FSGS	41
SSNS/MCD	32
STEC-associated HUS	16
TBMN	9
Tuberous Sclerosis	43
X-linked AS-Females	56
X-linked AS-Males	85

n = 22,197 diagnoses

Time in therapeutic window analyses

Inclusion criteria:
Participants with diagnosis date available

Participants with >3 eGFR values available

Excluded:

Rare Disease Group	n
All	14304
ADPKD	4510
ADTKD	136
AHUS	221
ANCA vasculitis	825
ARPKD NPHP	174
Anti-GBM	98
Bartter	44
Cystinosis	101
Cystinuria	248
Dent Disease and Lowe Syndrome	50
Gitelman	101
HNF1b Mutations	63
Hyperoxaluria	85
IgA Nephropathy	1785
MGRS	83
MPGN/C3G	669
Membranous Nephropathy	1014
Other Tubulopathies	109
Other Vasculitides	1602
Renal Cancer Inherited	108
Retroperitoneal Fibrosis	75
SRNS/FSGS	551
SSNS/MCD	724
STEC-associated HUS	158
TBMN	63
Tuberous Sclerosis	176
X-linked AS-Females	208
X-linked AS-Males	323

n = 12,990 diagnoses

Mortality analyses

Inclusion criteria:
Participants from England and Wales

For standardisation to CKD population only: eGFR at diagnosis

Excluded:

Standardised to English/Welsh population

Rare Disease Group	n
All	1337
ADPKD	382
ADTKD	3
AHUS	16
ANCA vasculitis	109
ARPKD NPHP	11
Anti-GBM	15
Bartter	3
Cystinosis	13
Cystinuria	12
Dent Disease and Lowe Syndrome	0
Gitelman	7
HNF1b Mutations	6
Hyperoxaluria	4
IgA Nephropathy	182
MGRS	3
MPGN/C3G	72
Membranous Nephropathy	109
Other Tubulopathies	6
Other Vasculitides	117
Renal Cancer Inherited	0
Retroperitoneal Fibrosis	1
SRNS/FSGS	93
SSNS/MCD	81
STEC-associated HUS	12
TBMN	9
Tuberous Sclerosis	1
X-linked AS-Females	39
X-linked AS-Males	31

n = 25,948 participants

Standardised to CKD population

Rare Disease Group	n
All	15568
ADPKD	4414
ADTKD	140
AHUS	231
ANCA vasculitis	1209
ARPKD NPHP	159
Anti-GBM	126
Bartter	38
Cystinosis	125
Cystinuria	187
Dent Disease and Lowe Syndrome	50
Gitelman	91
HNF1b Mutations	56
Hyperoxaluria	87
IgA Nephropathy	2695
MGRS	122
MPGN/C3G	725
Membranous Nephropathy	1101
Other Tubulopathies	76
Other Vasculitides	1423
Renal Cancer Inherited	107
Retroperitoneal Fibrosis	60
SRNS/FSGS	794
SSNS/MCD	660
STEC-associated HUS	161
TBMN	75
Tuberous Sclerosis	125
X-linked AS-Females	196
X-linked AS-Males	335

n = 11,717 participants

KRFE analyses

Inclusion criteria:
Participants with eGFR >15 and <60 ml/min/1.73m², available urine Albumin: Creatinine ratio result, and without kidney failure at recruitment

Participants with >2 years follow up since diagnosis date

Excluded:

Rare Disease Group	n
All	25048
ADPKD	7302
ADTKD	196
AHUS	280
ANCA vasculitis	2086
ARPKD NPHP	221
Anti-GBM	132
Bartter	51
Cystinosis	140
Cystinuria	458
Dent Disease and Lowe Syndrome	58
Gitelman	186
HNF1b Mutations	79
Hyperoxaluria	122
IgA Nephropathy	3678
MGRS	171
MPGN/C3G	1002
Membranous Nephropathy	2153
Other Tubulopathies	115
Other Vasculitides	2074
Renal Cancer Inherited	114
Retroperitoneal Fibrosis	129
SRNS/FSGS	1410
SSNS/MCD	1641
STEC-associated HUS	167
TBMN	152
Tuberous Sclerosis	241
X-linked AS-Females	292
X-linked AS-Males	398

n = 2,237 participants

*n=9 included patients had 2 recorded diagnoses. For Mortality and KFRE analyses these participants were analysed once, otherwise patients contributed data for each diagnosis

Supplementary Table 1: Number of creatinine measurements (taken prior to kidney failure) per person in each rare disease group

	Number of participants	Number with >0 creatinine measurements	Median number of measurements per individuals with >0 creatinine measurements (IQR)	Median number of measurements per year among individuals with >0 creatinine measurements (IQR)
All RaDaR	27,294	19,046	25 (12, 47)	4.5 (2.1, 9)
ADPKD	7797	5552	22 (11, 39)	3.5 (1.7, 6.5)
ADTKD	215	126	17.5 (10, 36)	3.5 (1.5, 6.9)
X-linked Alport Syndrome-Females	309	196	14.5 (6, 30.5)	1.9 (0.9, 4)
X-linked Alport Syndrome-Males	412	190	14 (7, 24)	2.9 (1.3, 7)
TBMN	165	134	13 (6, 26)	1.7 (0.9, 4)
ARPKD NPHP	233	148	21 (9, 42.5)	3.3 (1.6, 7.5)
Cystinosis	153	89	40 (18, 70)	5.7 (3.5, 9.8)
Cystinuria	473	347	12 (5, 22)	1.5 (0.8, 2.6)
Hyperoxaluria	124	77	12 (6, 25)	1.9 (0.9, 4.6)
HNF1B Mutations	86	55	13 (5, 39)	2.2 (0.9, 4.1)
Renal Cancer Inherited	114	9	4 (4, 9)	1 (0.6, 5.4)
Other Tubulopathies	117	69	20 (10, 29)	2.6 (1.7, 5.1)
Bartter	54	32	29.5 (15.5, 51.5)	5 (1.8, 8.2)
Gitelman	186	118	19.5 (8, 45)	2.5 (1.2, 4.8)
Dent Disease and Lowe Syndrome	62	33	13 (6, 22)	1.6 (1, 4.7)
Tuberous Sclerosis	249	170	12 (5, 20)	2 (1.1, 3.9)
AHUS	293	161	27 (9, 71)	9.8 (3.5, 23.4)
SSNS/MCD	1705	1381	22 (9, 44)	3.7 (1.6, 7.7)
SRNS/FSGS	1536	1246	25 (10, 50)	4.6 (2, 9.8)
IgA Nephropathy	4147	2943	23 (12, 41)	5.2 (2.7, 9.9)
Membranous Nephropathy	2439	1838	34 (17, 58)	5.6 (2.9, 10)
MGRS	181	122	42 (18, 76)	12.5 (6.2, 23.1)
MPGN/C3G	1089	685	25 (11, 52)	5.2 (2, 10.9)
Retroperitoneal Fibrosis	142	100	32 (12, 70)	5.2 (2.4, 9.3)
STEC-associated HUS	170	83	11 (3, 23)	2 (0.5, 4.5)
ANCA vasculitis	2375	1639	47 (24, 79)	8.4 (4.8, 14)
Anti-GBM	137	51	23 (5, 54)	31.6 (7.9, 264.8)
Other Vasculitides	2331	1452	34 (14, 67)	6.2 (2.8, 11.5)

Supplementary Figure 2: Cumulative incidence plot displaying Kaplan-Meier estimates of Kidney Failure censored for death

a) Glomerular b) Cystic c) Metabolic d) Tubular e) Other kidney conditions f) Alport Syndrome. Numerical values represent "number at risk (number censored)".

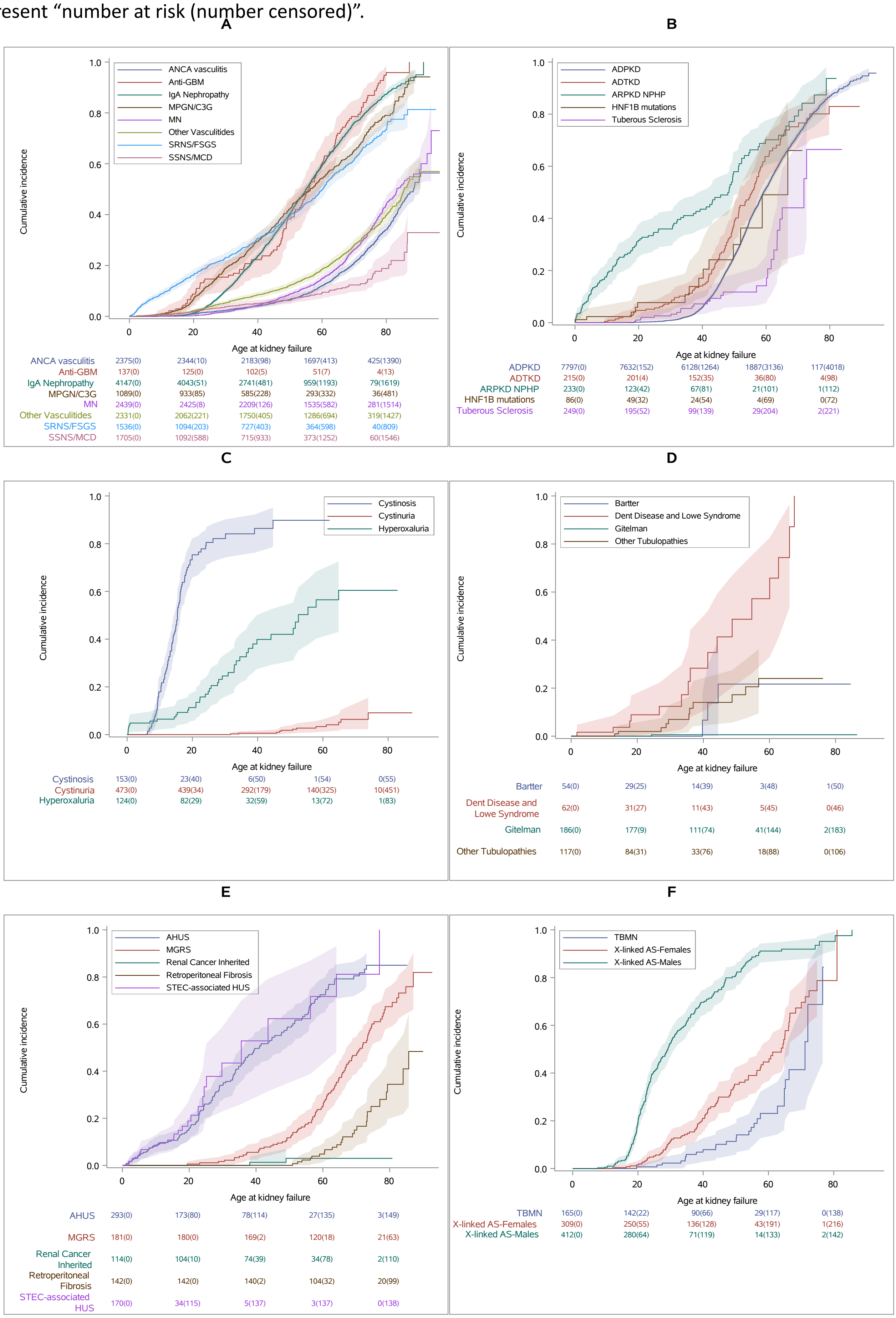

MN = Membranous Nephropathy; ANCA = Anti Neutrophil Cytoplasmic Antibodies; GBM = Glomerular Basement Membrane; AS = Alport syndrome

Supplementary Figure 3: Cumulative incidence of years from diagnosis to KF

a) Glomerular b) Cystic c) Metabolic d) Tubular e) Other Kidney Conditions f) Alport Syndrome, censored for death, and with death as a competing risk

a)

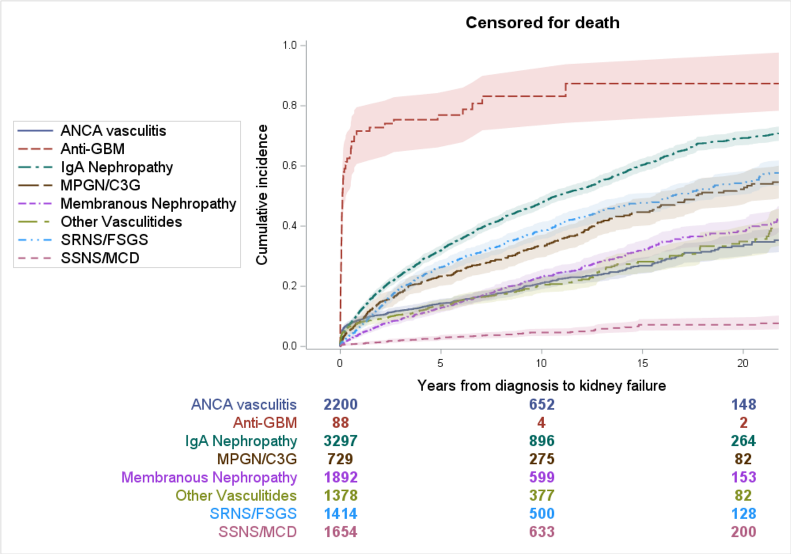

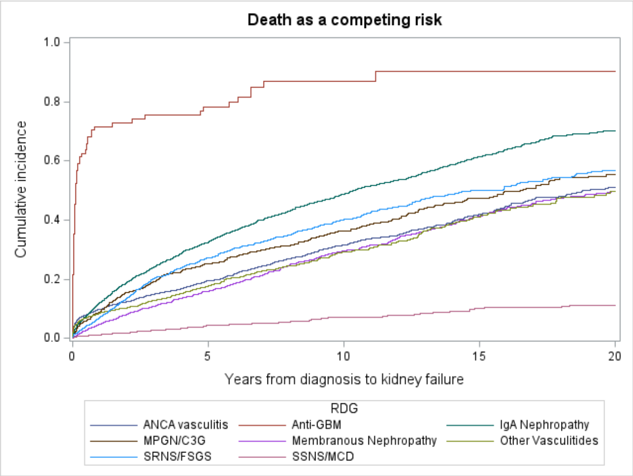

b)

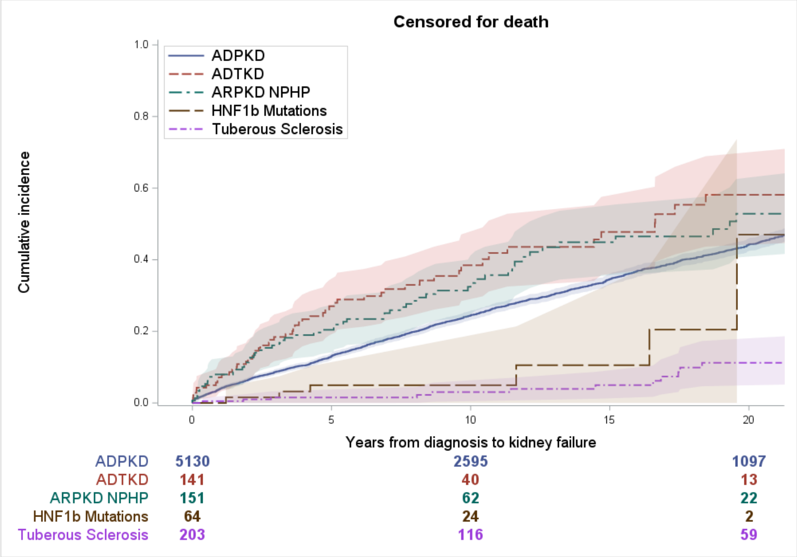

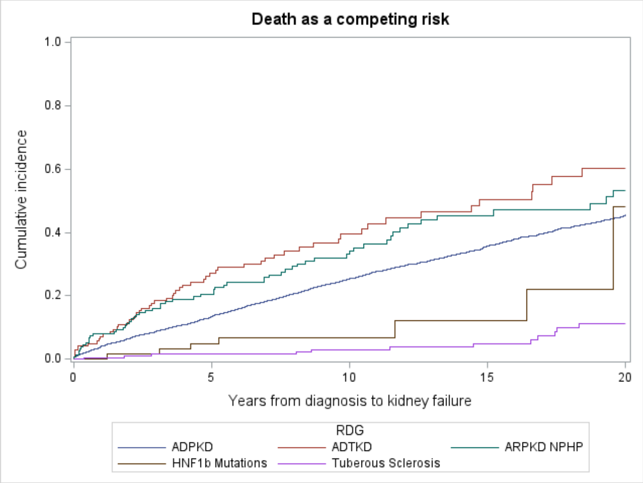

c)

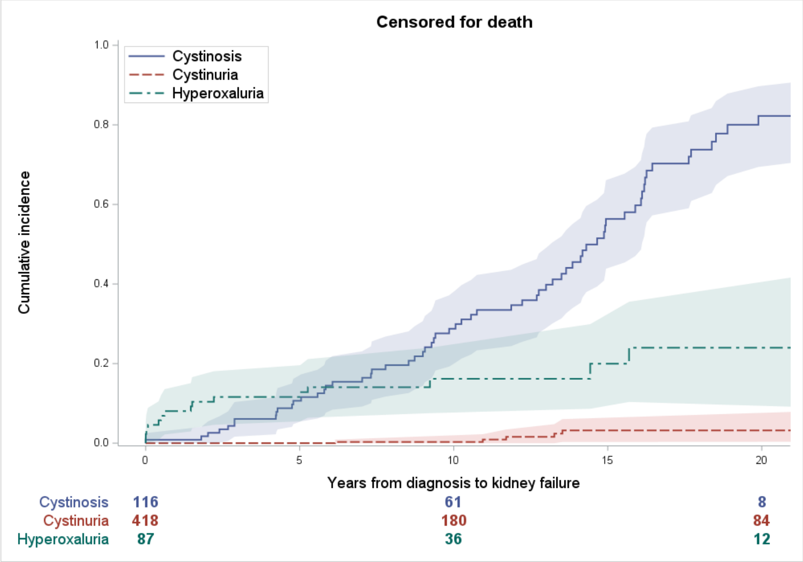

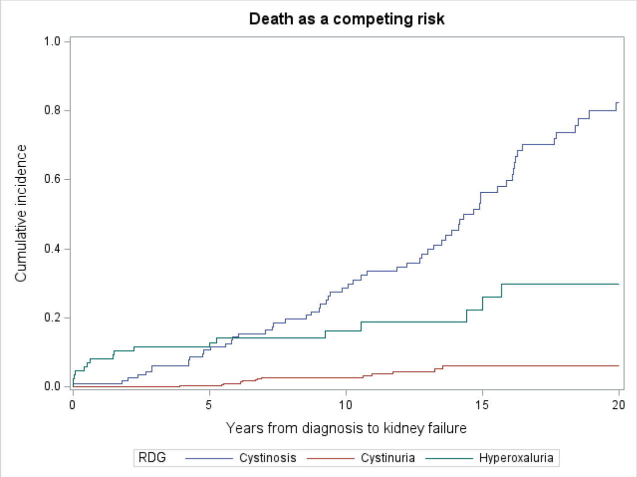

d)

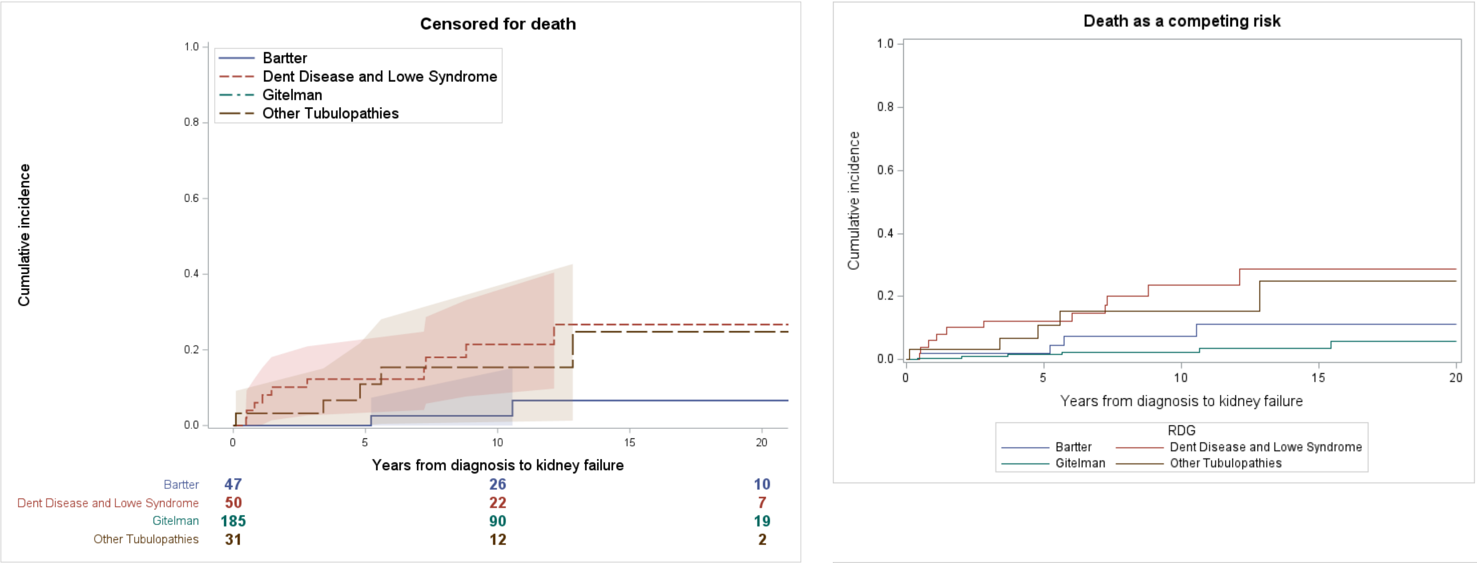

Censored for death
Bartter
Dent Disease and Lowe Syndrome
Gitelman
Other Tubulopathies
Cumulative incidence
Years from diagnosis to kidney failure
Bartter 47 26 10
Dent Disease and Lowe Syndrome 50 22 7
Gitelman 185 90 19
Other Tubulopathies 31 12 2
Death as a competing risk
Cumulative incidence
Years from diagnosis to kidney failure
RDG
Bartter
Dent Disease and Lowe Syndrome
Gitelman
Other Tubulopathies

e)

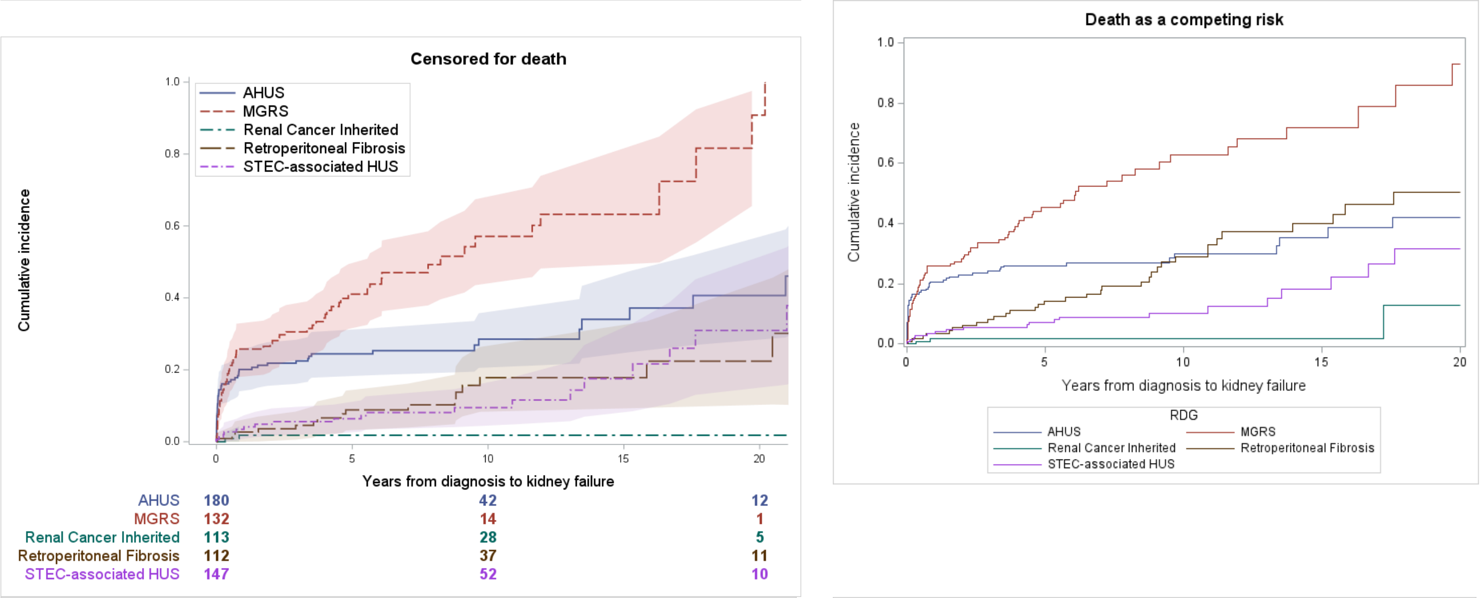

Censored for death
AHUS
MGRS
Renal Cancer Inherited
Retroperitoneal Fibrosis
STEC-associated HUS
Cumulative incidence
Years from diagnosis to kidney failure
AHUS 180 42 12
MGRS 132 14 1
Renal Cancer Inherited 113 28 5
Retroperitoneal Fibrosis 112 37 11
STEC-associated HUS 147 52 10
Death as a competing risk
Cumulative incidence
Years from diagnosis to kidney failure
RDG
AHUS
MGRS
Renal Cancer Inherited
Retroperitoneal Fibrosis
STEC-associated HUS

f)

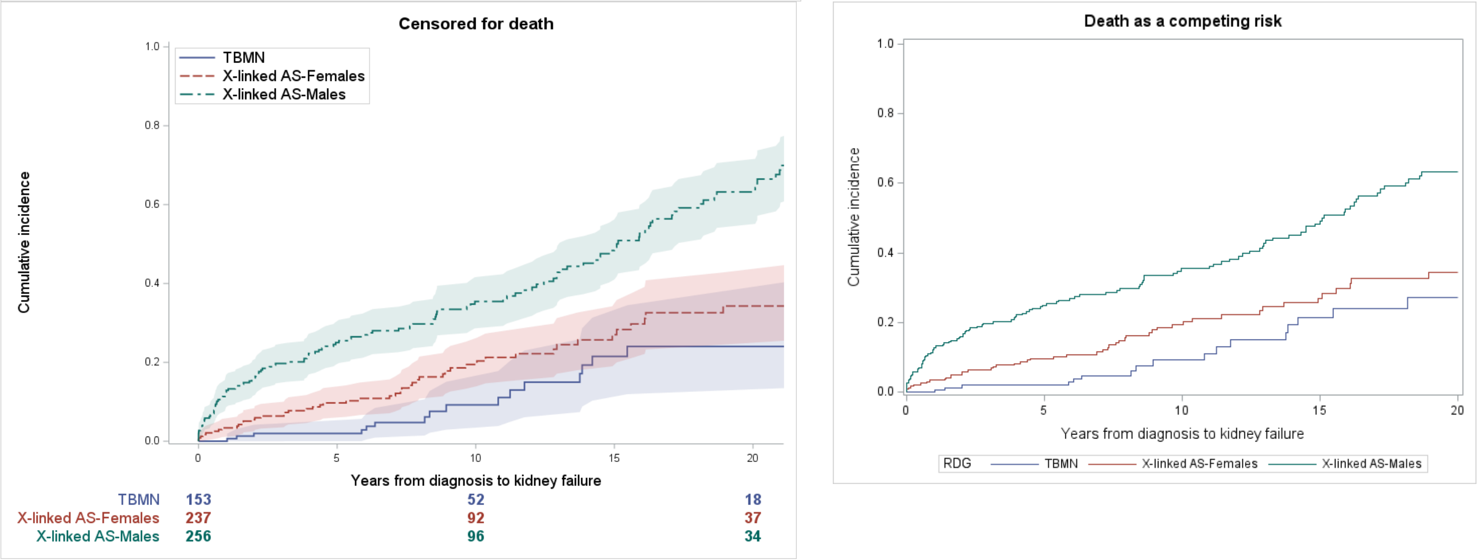

Censored for death
TBMN
X-linked AS-Females
X-linked AS-Males
Cumulative incidence
Years from diagnosis to kidney failure
TBMN 153 52 18
X-linked AS-Females 237 92 37
X-linked AS-Males 256 96 34
Death as a competing risk
Cumulative incidence
Years from diagnosis to kidney failure
RDG
TBMN
X-linked AS-Females
X-linked AS-Males

Supplementary Figure 4: Kaplan Meier Survival analyses of Age at Death

a) Glomerular b) Cystic c) Metabolic d) Tubular e) Other kidney conditions f) Alport syndrome

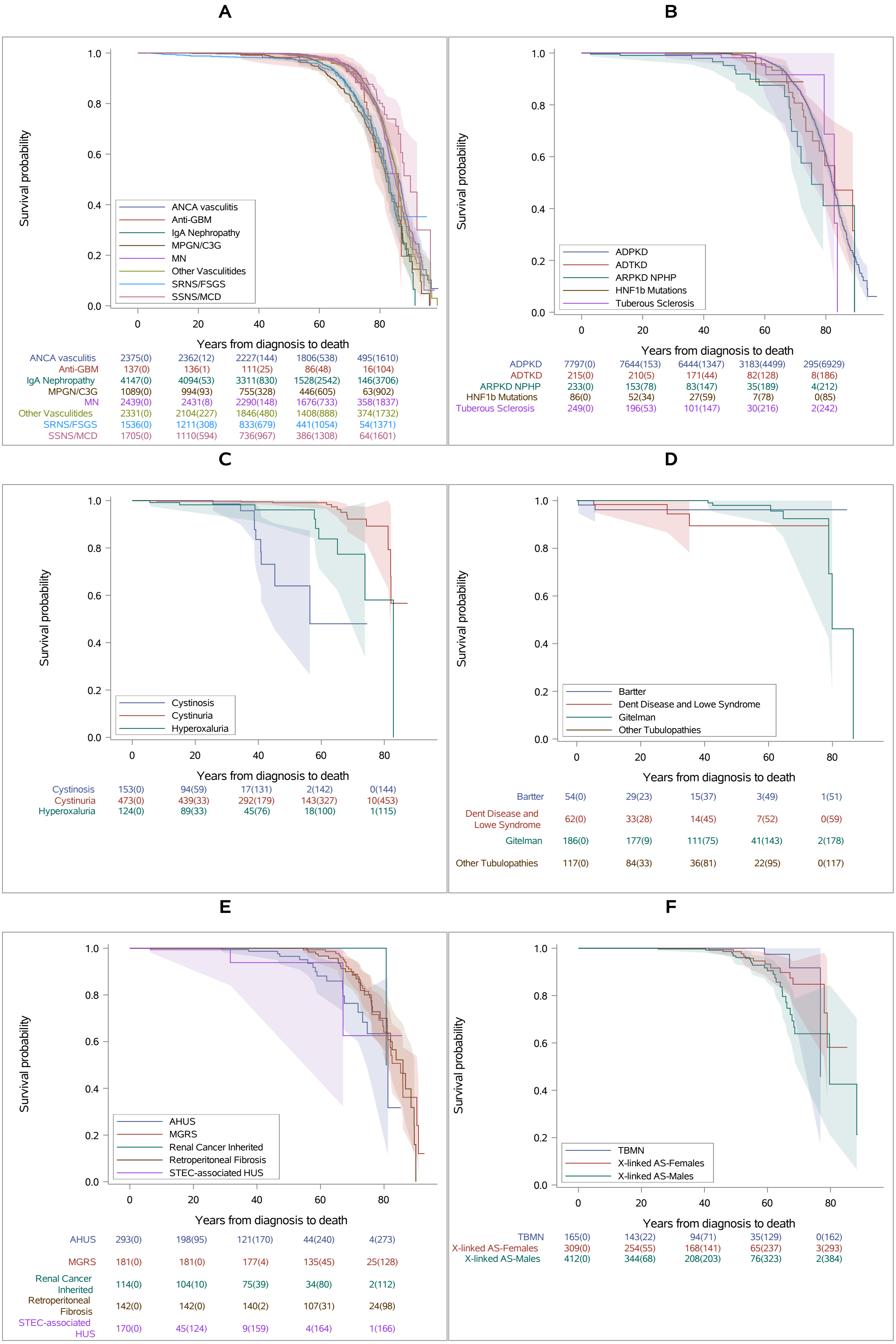

MN = Membranous Nephropathy; ANCA = Anti Neutrophil Cytoplasmic Antibodies; GBM = Glomerular Basement Membrane; AS = Alport syndrome

Supplementary Figure 5: Estimated eGFR trajectories plotting Kaplan-Meier estimates of median time from diagnosis to eGFR value

a) Glomerular b) Cystic kidney diseases. Data for other diseases are shown in Supplement page c) Metabolic d) Other kidney conditions e) Alport Syndrome

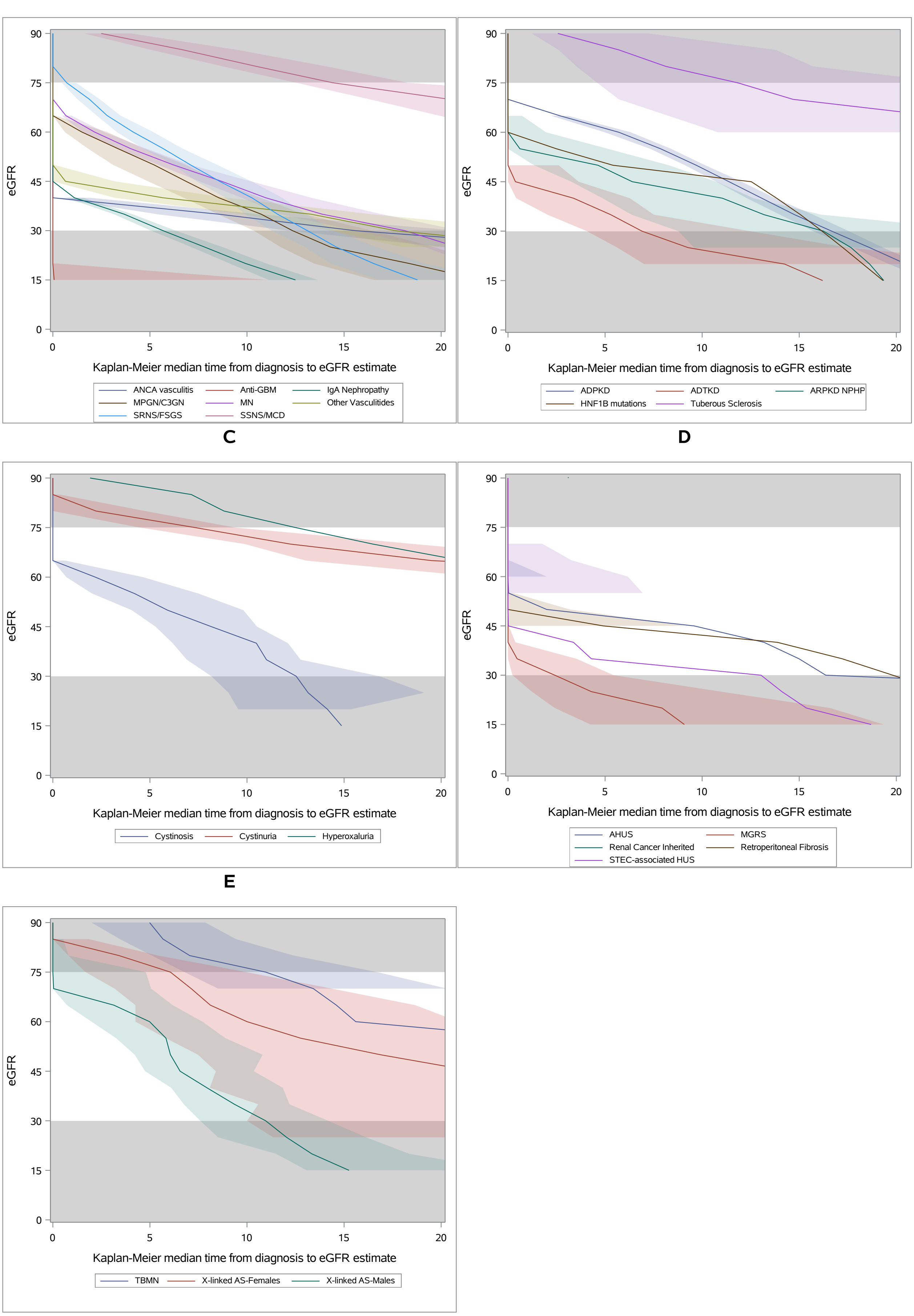

Tubulopathies not presented due to very small numbers of patients reaching each eGFR estimate. MN = Membranous Nephropathy; ANCA = Anti Neutrophil Cytoplasmic Antibodies; GBM = Glomerular Basement Membrane; AS = Alport syndrome

Supplementary Figure 6: Age stratified Kaplan Meier Survival analyses of years from dialysis start to death (not censored for transplant)

a) RaDaR patients (*Recruited*) b) UKRR Patients eligible for RaDaR but not recruited (*Eligible*) c) Patients with a Primary Renal Disease of Diabetes, Renovascular Disease or Hypertension (*Other PRD*). Numerical values represent "number at risk (number censored)".

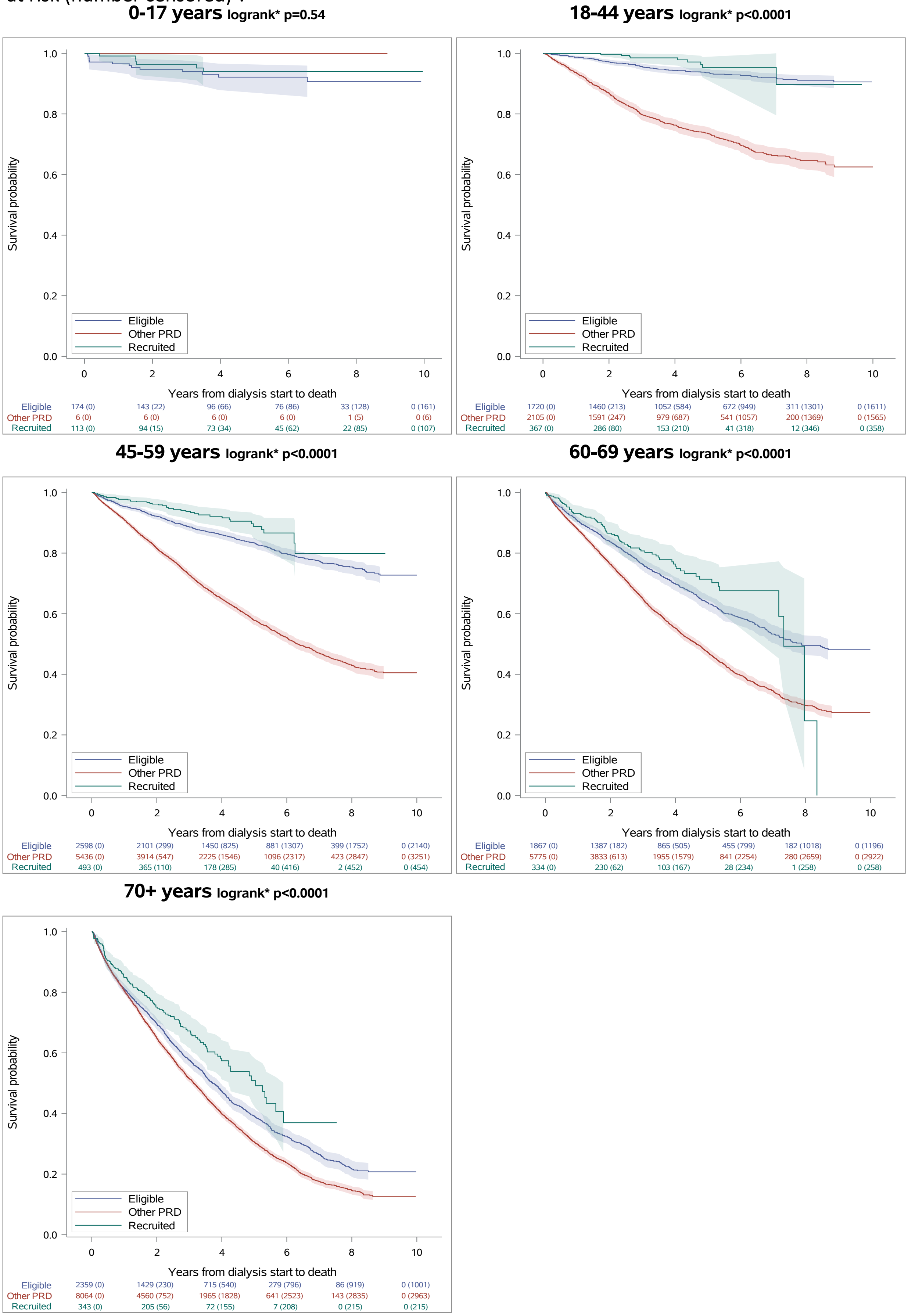

***Pairwise logrank statistic between RaDaR patients and patients with PRD of Diabetes, Hypertension or Renovascular Disease. PRD = Primary Renal Disease.**

Supplementary Table 2: 1-, 3- and 5- year cumulative incidence of Kidney Failure in the RaDaR and UK CKD populations

	RaDaR population (%) (95% CI)	UK CKD population* (%)
**1 year**	8% (7%, 9%)	0.2%
**3 year**	19% (18%, 20%)	0.6%
**5 year**	28% (26%, 29%)	1%

*UK population prevalence estimates of CKD stages 3-5 using QICKD study and National CKD audit data, RRT incidence data from UKRR.

Supplementary Figure 7: Kidney Replacement Therapy incidence 2017-2021 per 100 patients

(a) i) RaDaR ii) UK population* iii) CKD population** (b) for i) RaDaR ii) CKD population**, stratified by primary renal disease

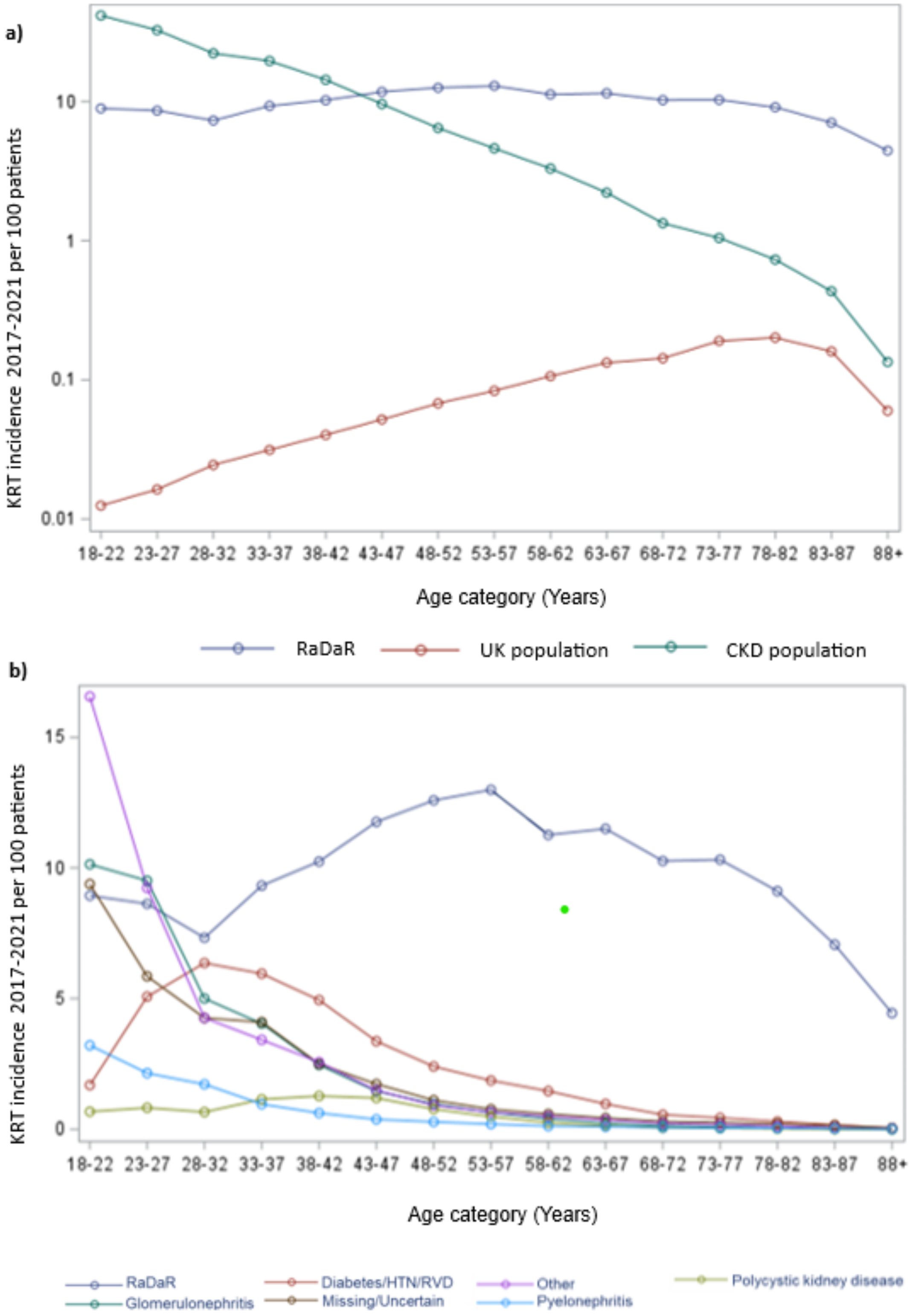

*Data from Office of National Statistics ** Data from Quality Improvement in CKD study and the National CKD Audit

Supplementary Figure 8: Calibration plot for 2-year 4 variable Kidney Failure Risk Equation (KFRE) for RaDaR patients stratified by age group

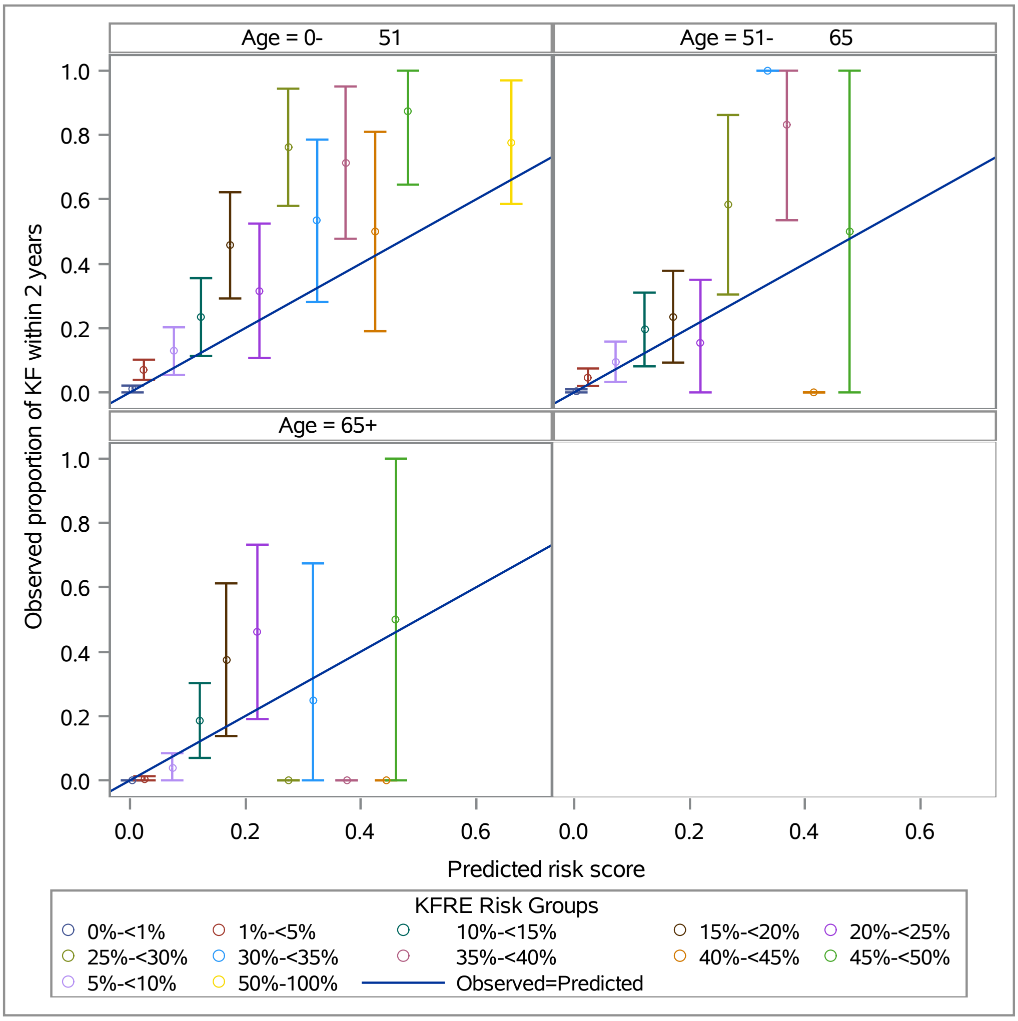

Patients with eGFR > 15 and <60ml/min at recruitment who remained alive with >2 years of follow-up data.

Supplementary Table 3: Median age at diagnosis, by Sex

	Median age at diagnosis (Years) [IQR]				P-value**
	Males		Females		
**All RaDaR***	43	[26.0- 58.3]	39	[22.7- 56.2]	
**ADPKD**	40	[29.1- 51.1]	36	[24.8- 48.3]	<0.0001
**ADTKD**	43	[27.5- 53.5]	42	[27.8- 54.9]	0.82
**X-linked AS**	19	[8.2- 34.5]	26	[7.6- 40.2]	0.015
**TBMN**	33	[16.1- 47.0]	31	[19.2- 45.2]	0.96
**ARPKD/NPHP**	4	[0.0- 25.4]	12	[0.6- 34.4]	0.10
**Cystinosis**	2	[0.7- 9.9]	2	[0.5- 10.0]	0.70
**Cystinuria**	33	[18.2- 52.1]	30	[18.8- 44.2]	0.092
**Hyperoxaluria**	17	[1.2- 37.0]	20	[8.2- 33.6]	0.69
**HNF1b Mutations**	13	[1.3- 34.5]	15	[5.3- 34.5]	0.45
**Renal Cancer Inherited**	46	[30.8- 57.1]	39	[22.8- 52.8]	0.29
**Other Tubulopathies**	13	[5.3- 42.5]	20	[7.8- 41.9]	0.69
**Bartter Syndrome**	7	[0.4- 22.4]	4	[0.3- 36.1]	0.75
**Gitelman Syndrome**	34	[17.2- 45.5]	31	[21.8- 43.9]	0.91
**Dent Disease and Lowe Syndrome**	9	[1.0- 29.2]	11	[10.9- 10.9]	***
**Tuberous Sclerosis Complex**	15	[3.1- 31.4]	16	[2.4- 34.7]	0.77
**aHUS**	22	[3.3- 41.4]	20	[3.4- 38.9]	0.84
**SSNS & minimal change disease**	11	[3.7- 44.7]	24	[5.3- 47.5]	0.0017
**SRNS & FSGS**	31	[7.8- 51.0]	23	[6.3- 49.9]	0.019
**IgA nephropathy**	42	[30.7- 53.7]	38	[27.7- 47.5]	<0.0001
**Membranous Nephropathy**	57	[45.6- 66.1]	58	[44.7- 68.6]	0.78
**MGRS**	61	[47.3- 71.9]	63	[53.9- 72.9]	0.18
**MPGN/C3G**	38	[15.5- 58.3]	31	[14.6- 53.6]	0.039
**Retroperitoneal Fibrosis**	57	[51.7- 68.9]	57	[51.3- 64.1]	0.46
**STEC HUS**	3	[1.6- 6.4]	4	[2.0- 9.5]	0.059
**ANCA associated vasculitis**	63	[51.2- 70.3]	62	[49.3- 70.7]	0.050
**Anti-GBM disease**	40	[29.1- 51.1]	58	[44.8- 67.6]	0.011
**Other Vasculitides**	43	[27.5- 53.5]	52	[24.4- 66.9]	0.77

*Excluding X-linked AS **T-test for groups with >30 patients, Mann-Whitney U test for those with ≤30 patients in each group
***too few female patients to perform test

Supplementary Table 4: Median eGFR at diagnosis, by Sex

	Median eGFR at diagnosis [IQR]				P-value**
	Males		Females		
**All RaDaR***	50.8	[27.2- 84.8]	60.5	[28.7- 94.5]	
**ADPKD**	63.4	[32.8- 90.3]	69.2	[37.9- 98.6]	0.0011
**ADTKD**	43.2	[21.7- 57.6]	46.6	[28.7- 93.2]	0.26
**X-linked AS**	67.7	[23.9- 114.9]	84.9	[54.6- 116.9]	0.10
**TBMN**	83.7	[41.1 100.5]	99.8	[85.9- 112.6]	0.02
**ARPKD/NPHP**	48.2	[31.1- 85.4]	56.9	[40.3- 76.9]	0.71
**Cystinosis**	Insufficient data		Insufficient data		0.43
**Cystinuria**	84.1	[67.3- 99.4]	79.3	[69.7- 100.0]	0.94
**Hyperoxaluria**	Insufficient data		Insufficient data		-
**HNF1b Mutations**	Insufficient data		Insufficient data		-
**Renal Cancer Inherited**	Insufficient data		Insufficient data		-
**Other Tubulopathies**	Insufficient data		Insufficient data		-
**Bartter Syndrome**	Insufficient data		Insufficient data		-
**Gitelman Syndrome**	100.4	[79.1- 110.7]	108.8	[98.4- 121.5]	0.13
**Dent Disease and Lowe Syndrome**	Insufficient data		Insufficient data		-
**Tuberous Sclerosis Complex**	97.3	[53.2- 113.9]	89.5	[65.0- 103.6]	0.65
**aHUS**	39.9	[14.5- 80.1]	26.8	[10.6- 66.9]	0.26
**SSNS & minimal change disease**	90.5	[66.4- 119.8]	92.6	[62.7- 115.6]	0.97
**SRNS & FSGS**	69.7	[40.1- 107.6]	86.5	[41.7- 118.8]	0.06
**IgA nephropathy**	37.8	[22.9- 61.4]	50.0	[23.9- 78.5]	<0.0001
**Membranous Nephropathy**	67.0	[43.6- 89.7]	68.4	[44.0- 90.9]	0.36
**MGRS**	23.8	[15.4- 55.7]	35.6	[13.5- 60.0]	0.85
**MPGN/C3G**	56.7	[29.0- 95.2]	66.4	[36.7- 99.9]	0.25
**Retroperitoneal Fibrosis**	Insufficient data		Insufficient data		-
**STEC HUS**	Insufficient data		Insufficient data		-
**ANCA associated vasculitis**	31.4	[19.0- 50.5]	29.5	[17.9- 51.4]	0.94
**Anti-GBM disease**	10.9	[6.8- 15.0]	11.6	[6.4- 21.4]	0.80
**Other Vasculitides**	39.9	[25.3- 72.9]	30.6	[14.2- 68.4]	0.11

*Excluding X-linked AS **T-test for groups with >30 patients, Mann-Whitney U test for those with ≤30 patients in each group
Insufficient data= <10 patients in each group with eGFR data at diagnosis available.

Supplementary Table 5: Time from diagnosis to Kidney Failure and 10-year renal survival, stratified by Rare Disease Group and Sex

*Excluding X,linked AS, except for total (n). Data for patients with more than one diagnosis presented for each diagnosis. LQ, Lower Quartile Estimate.

. = Insufficient data to calculate an estimate. † Logrank statistic

	n (%)				10-year renal survival (95% CI)		Median years from diagnosis to KF (95% CI)						
	Males		Females		Males	Females	Males			Females			P-value†
All RaDaR*	15175	(55.6)	12110	(44.4)	0.70 (0.69, 0.71)	0.77 (0.76,0.78)	20.6	(19.7,	21.4)	27.4	(26.1,	28.3)	
ADPKD	3738	(47.9)	4059	(52.1)	0.72 (0.7, 0.74)	0.79 (0.77, 0.81)	19.6	(18.5,	20.8)	26.3	(24.8,	28.0)	<0.0001
ADTKD	90	(41.9)	125	(58.1)	0.61 (0.45, 0.74)	0.62 (0.49, 0.73)	18.4	(9.59,	.	16.6	(8.68,	25.9)	0.59
X-linked AS	412	(57.1)	309	(42.9)	0.65 (0.58, 0.71)	0.81 (0.74, 0.86)	15.1	(12.9,	17.2)	40.3	23.8	52.7	<0.0001
TBMN	54	(32.7)	111	(67.3)	0.92 (0.76, 0.98)	0.9 (0.79, 0.96)	25.9	(14.2,	.	33.3	(33.3,	.	0.19
ARPKD/NPHP	114	(48.9)	119	(51.1)	0.69 (0.56, 0.78)	0.63 (0.5, 0.74)	26.1	(11.5,	.	19.3	(8.40,	26.7)	0.69
Cystinosis	75	(49.0)	78	(51.0)	0.71 (0.56, 0.81)	0.72 (0.57, 0.83)	14.2	(10.8,	16.1)	14.9	(12.2,	17.7)	0.92
Cystinuria	251	(53.1)	222	(46.9)	1 (1, 1)	0.99 (0.96, 1)	.	.	.	.	.	.	0.82
Hyperoxaluria	78	(62.9)	46	(37.1)	0.81 (0.66, 0.9)	0.88 (0.72, 0.95)	.	.	.	42.0	(42.0,	.	0.56
HNF1b Mutations	47	(54.7)	39	(45.3)	0.94 (0.77, 0.98)	0.97 (0.79, 1)	.	.	.	19.6	(11.6,	.	0.79
Renal Cancer Inherited	46	(40.4)	68	(59.6)	1 (1, 1)	0.97 (0.89, 0.99)	.	.	.	.	.	.	0.25
Other Tubulopathies	58	(49.6)	59	(50.4)	0.78 (0.46, 0.93)	0.91 (0.51, 0.99)	.	(4.79,	.	.	.	.	0.17
Bartter Syndrome	37	(68.5)	17	(31.5)	1 (1, 1)	0.9 (0.47, 0.99)	.	.	.	.	(5.21,	.	0.37
Gitelman Syndrome	62	(33.3)	124	(66.7)	1 (1, 1)	1 (1, 1)	.	.	.	.	.	.	.
Dent Disease and Lowe Syndrome	61	(98.4)	1	(1.6)	0.81 (0.65, 0.9)	0 (., .)	.	.	.	7.29	.	.	0.066
Tuberous Sclerosis Complex	102	(41.0)	147	(59.0)	0.96 (0.87, 0.99)	0.98 (0.91, 0.99)	61.0	(27.7,	.	.	.	.	0.13
aHUS	133	(45.4)	160	(54.6)	0.76 (0.64, 0.84)	0.69 (0.58, 0.78)	21.0	(15.2,	.	21.7	(13.5,	.	0.86
SSNS & minimal change disease	1004	(58.9)	701	(41.1)	0.96 (0.94, 0.97)	0.95 (0.92, 0.96)	54.3	(50.3,	.	.	(48.1,	.	0.41
SRNS & FSGS	872	(56.8)	664	(43.2)	0.59 (0.55, 0.63)	0.64 (0.6, 0.68)	15.9	(12.5,	18.4)	18.5	(14.1,	21.0)	0.13
IgA nephropathy	2924	(70.5)	1223	(29.5)	0.49 (0.47, 0.51)	0.6 (0.56, 0.63)	9.7	(8.8,	10.4)	14.3	(13.0,	16.6)	<0.0001
Membranous nephropathy	1634	(67.0)	805	(33.0)	0.76 (0.74, 0.79)	0.78 (0.73, 0.81)	26.8	(21.9,	36.6)	29.1	(24.5,	36.8)	0.71
MGRS	96	(53.0)	85	(47.0)	0.37 (0.22, 0.53)	0.51 (0.34, 0.66)	8.25	(4.0,	11.6)	11.9	(4.23,	.	0.76
MPGN/C3G	581	(53.4)	508	(46.6)	0.62 (0.57, 0.67)	0.71 (0.66, 0.76)	13.3	(12.1,	16.7)	25.5	(20.0,	29.3)	0.0031
Retroperitoneal Fibrosis	94	(66.2)	48	(33.8)	0.81 (0.67, 0.9)	0.84 (0.61, 0.94)	26.5	(20.5,	.	.	.	.	0.61
STEC HUS	83	(48.8)	87	(51.2)	0.87 (0.74, 0.93)	0.94 (0.85, 0.98)	42.1	(21.0,	.	17.6	(13.5,	.	0.86
ANCA associated vasculitis	1278	(53.8)	1097	(46.2)	0.78 (0.75, 0.81)	0.8 (0.77, 0.83)	27.9	(23.4,	35.6	LQ 14.8	(12.2,	17.1)	0.25
Anti,GBM disease	68	(49.6)	69	(50.4)	0.17 (0.07, 0.32)	0.18 (0.08, 0.31)	0.12	(0.06,	0.69	0.10	(0.05,	0.27)	0.41
Other Vasculitides	1187	(50.9)	1144	(49.1)	0.79 (0.75, 0.83)	0.8 (0.76, 0.83)	33.4	(25.4,	48.8	26.9	(21.3,	.	0.95

Supplementary Figure 9: Time from diagnosis to Kidney Failure for Males and Females, by Rare Disease Group*

ADPKD p-value <0.0001

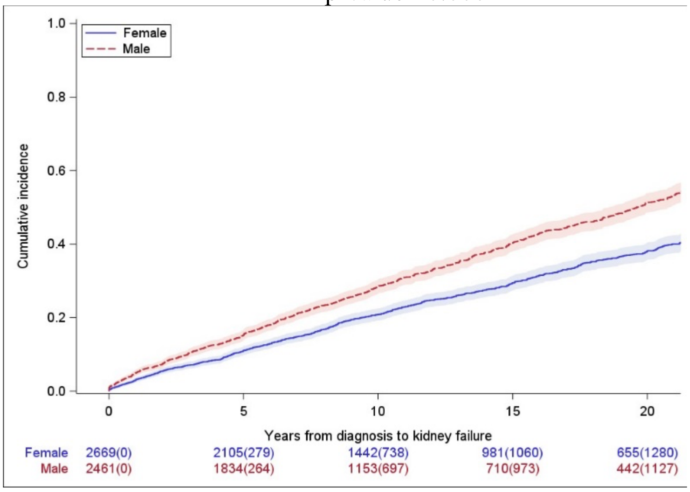

ADTKD p-value= 0.59

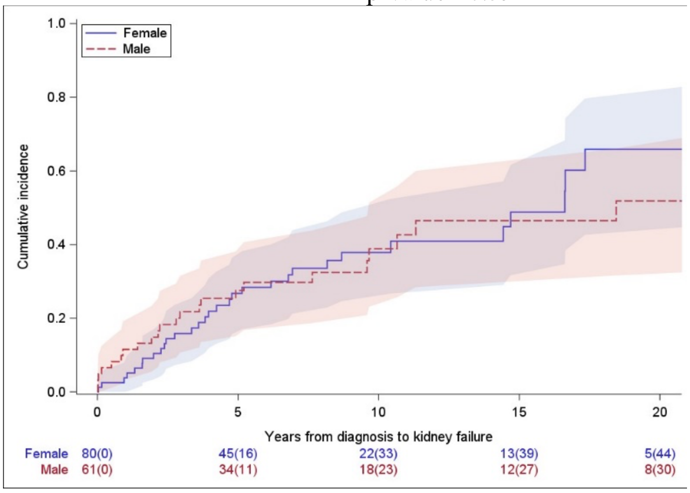

aHUS p-value= 0.86

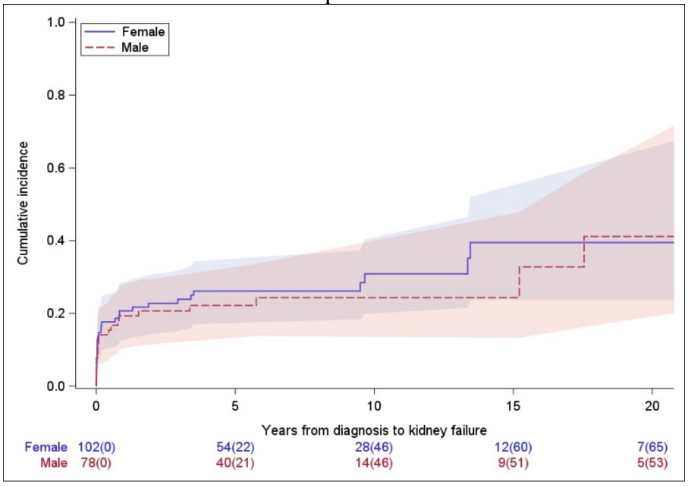

ANCA Vasculitis p-value= 0.25

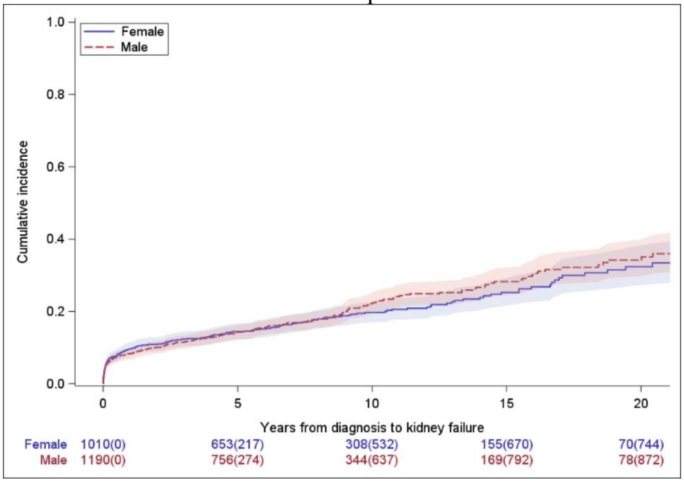

Anti-GBM disease p-value= 0.41

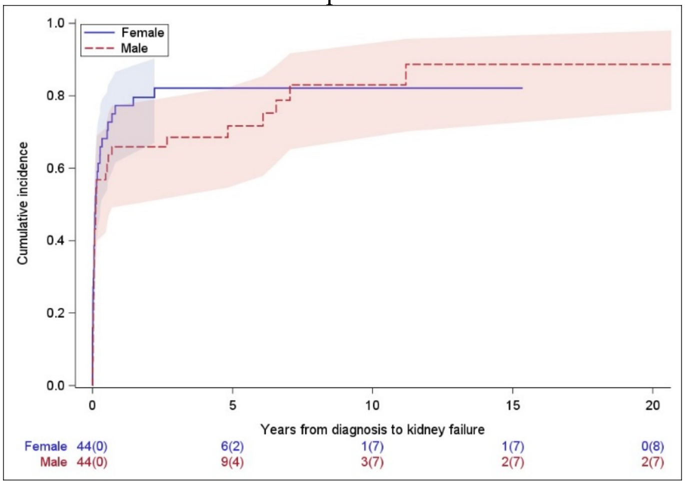

ARPKD/NPHP p-value= 0.69

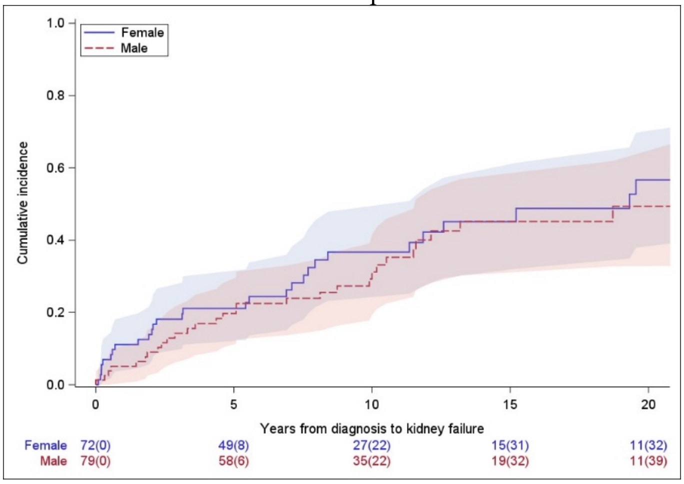

Cystinosis p-value= 0.92

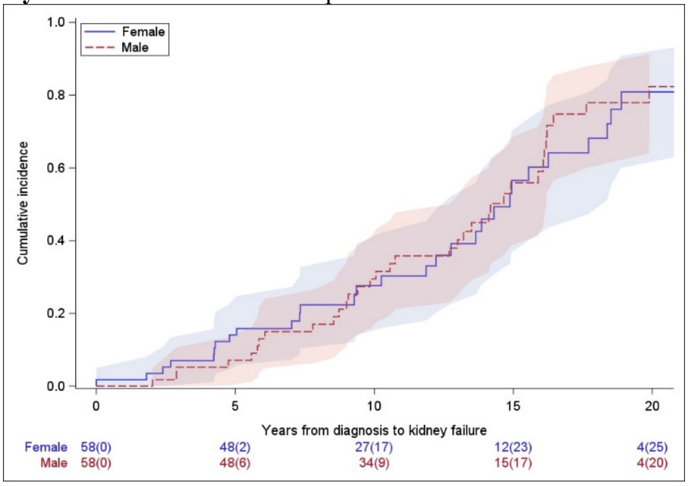

Cystinuria p-value= 0.82

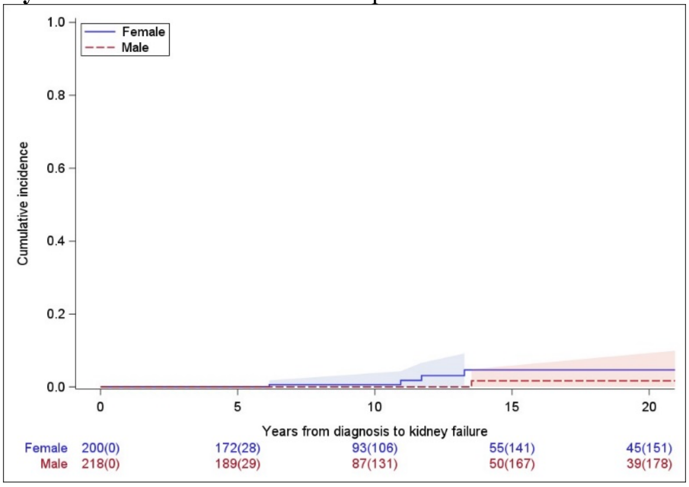

HNF1B mutations p-value= 0.79

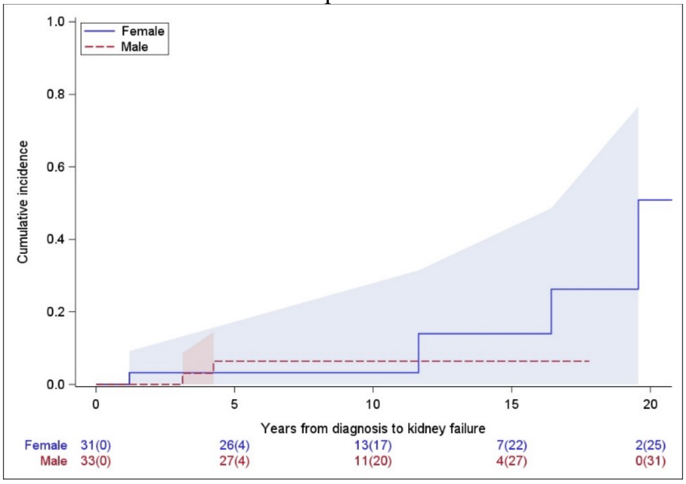

Hyperoxaluria p-value= 0.56

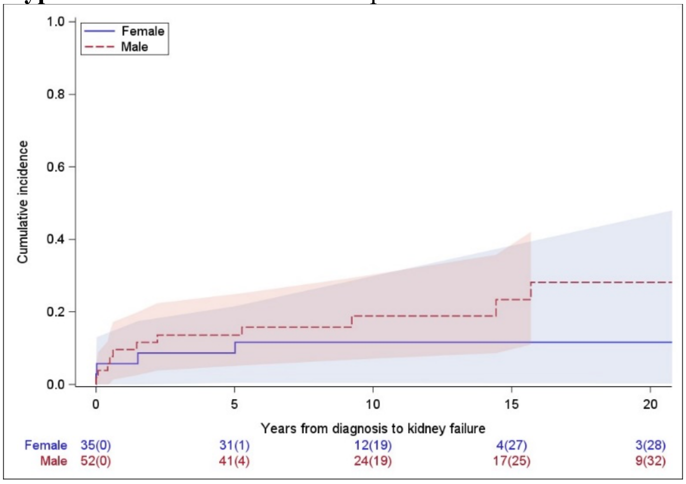

IgA nephropathy p-value <0.0001

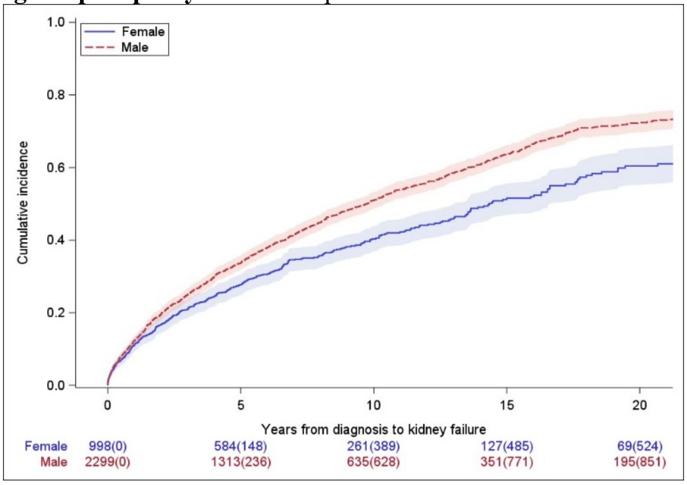

Membranous nephropathy p-value= 0.71

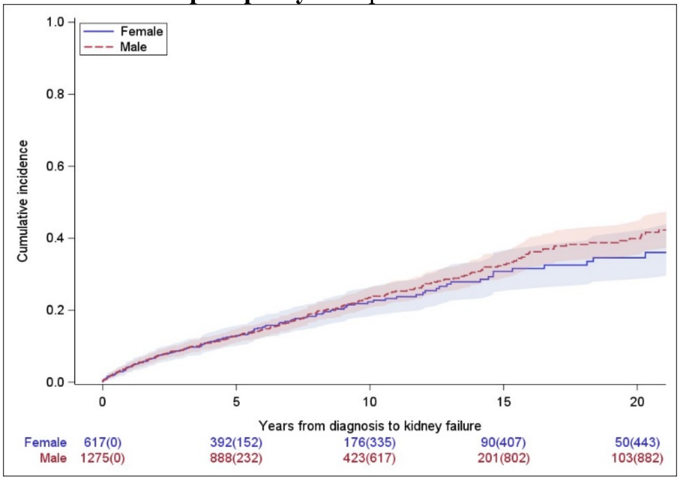

MGRS p-value= 0.76

MPGN/C3G p-value= 0.003

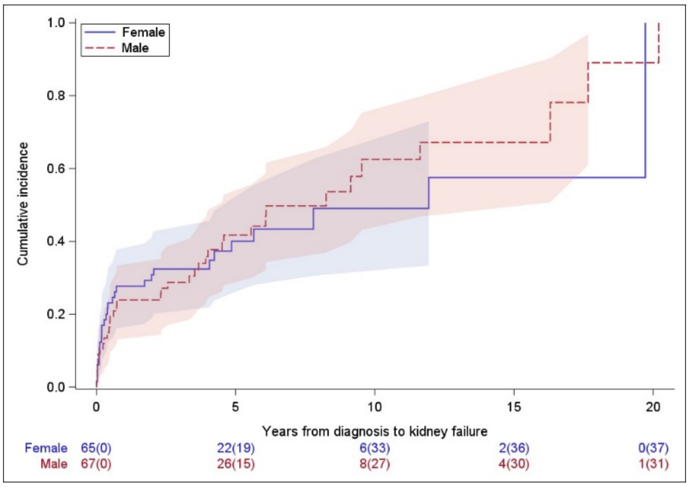

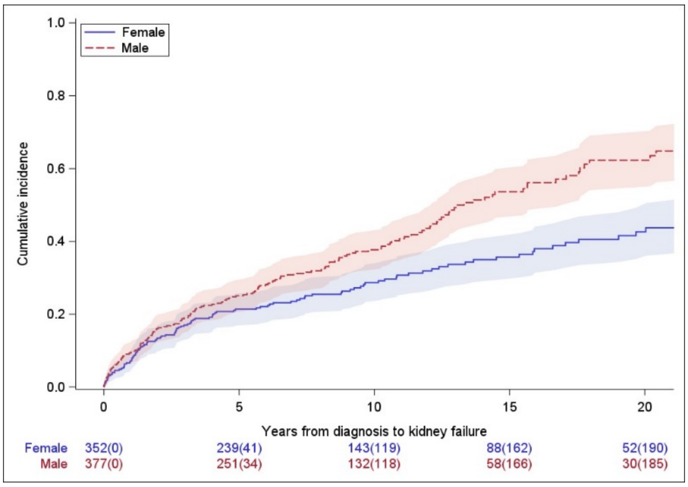

Other Tubulopathies p-value= 0.17

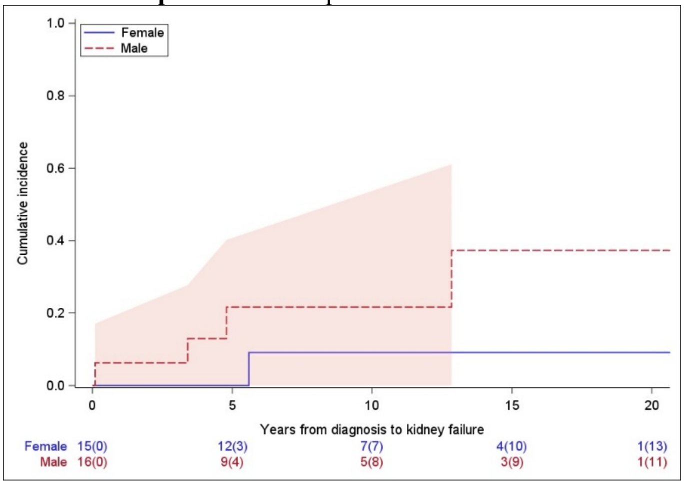

Other Vasculitides p-value= 0.95

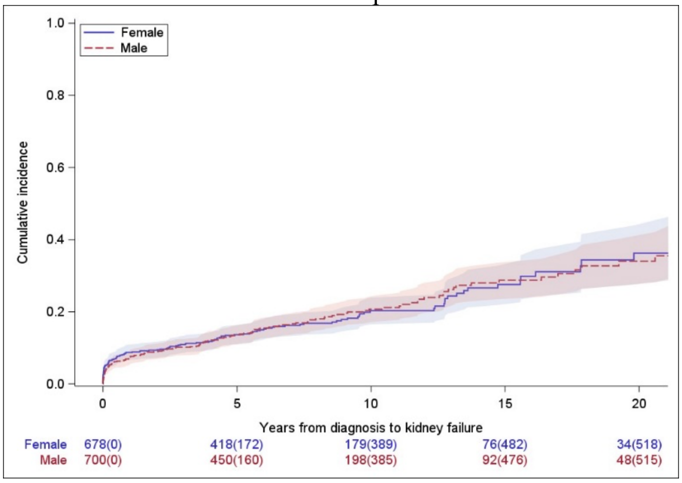

Retroperitoneal Fibrosis p-value= 0.61

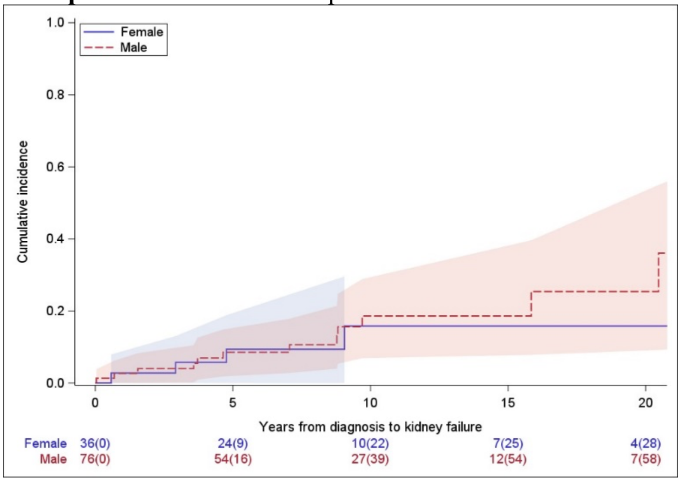

SRNS/FSGS p-value= 0.13

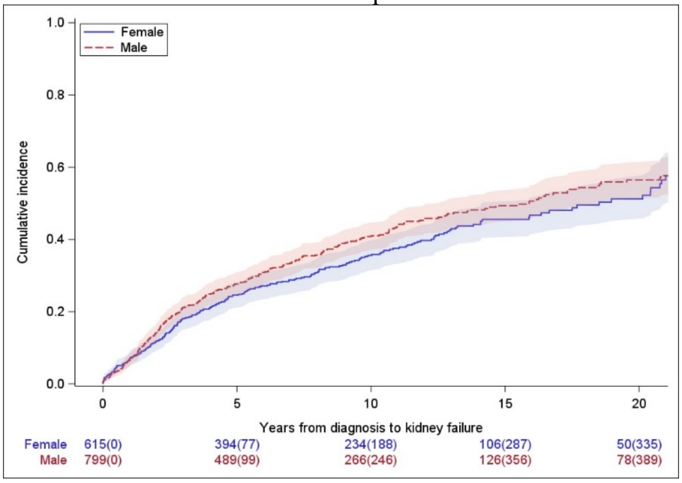

SSNS/MCD p-value= 0.41

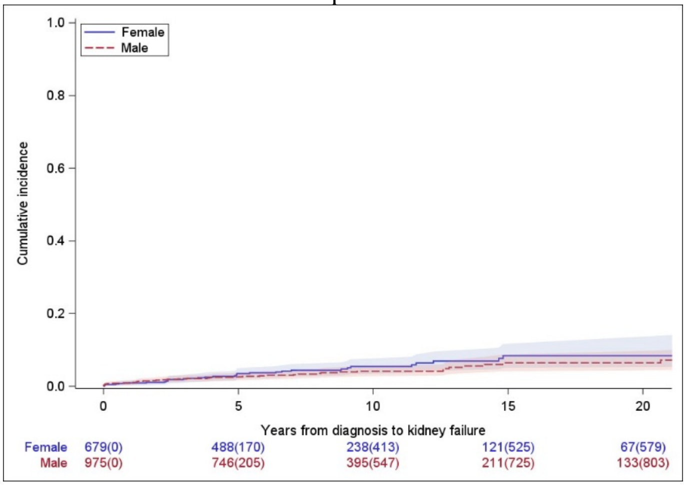

STEC-HUS p-value= 0.86

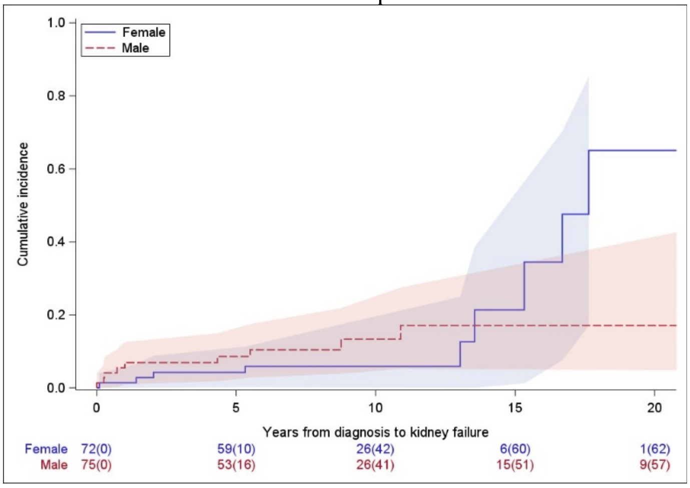

TBMN p-value= 0.19

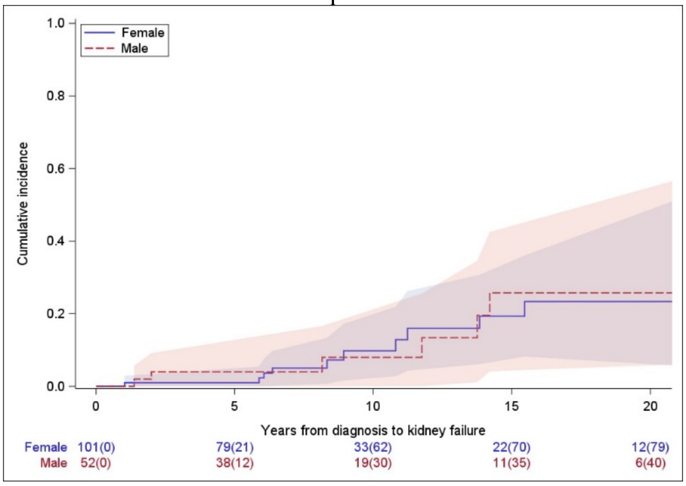

Tuberous Sclerosis Complex p-value= 0.13

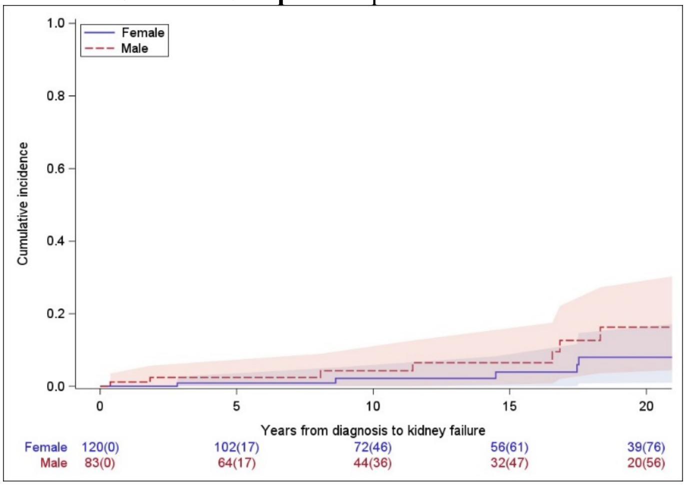

*X-linked Alport Syndrome presented stratified by Sex in Supplementary Figure 2. Bartter Syndrome, Dent Disease and Lowe, Gitelman Syndrome and Inherited Renal Cancers are not presented due to <5 events across both Males and Females

Supplementary Figure 10: Time from diagnosis to Kidney Failure for Males and Females with ADPKD, IgA nephropathy and MPGN/C3G, stratified by age quartile at diagnosis
Numerical values represent "number at risk (number censored)".

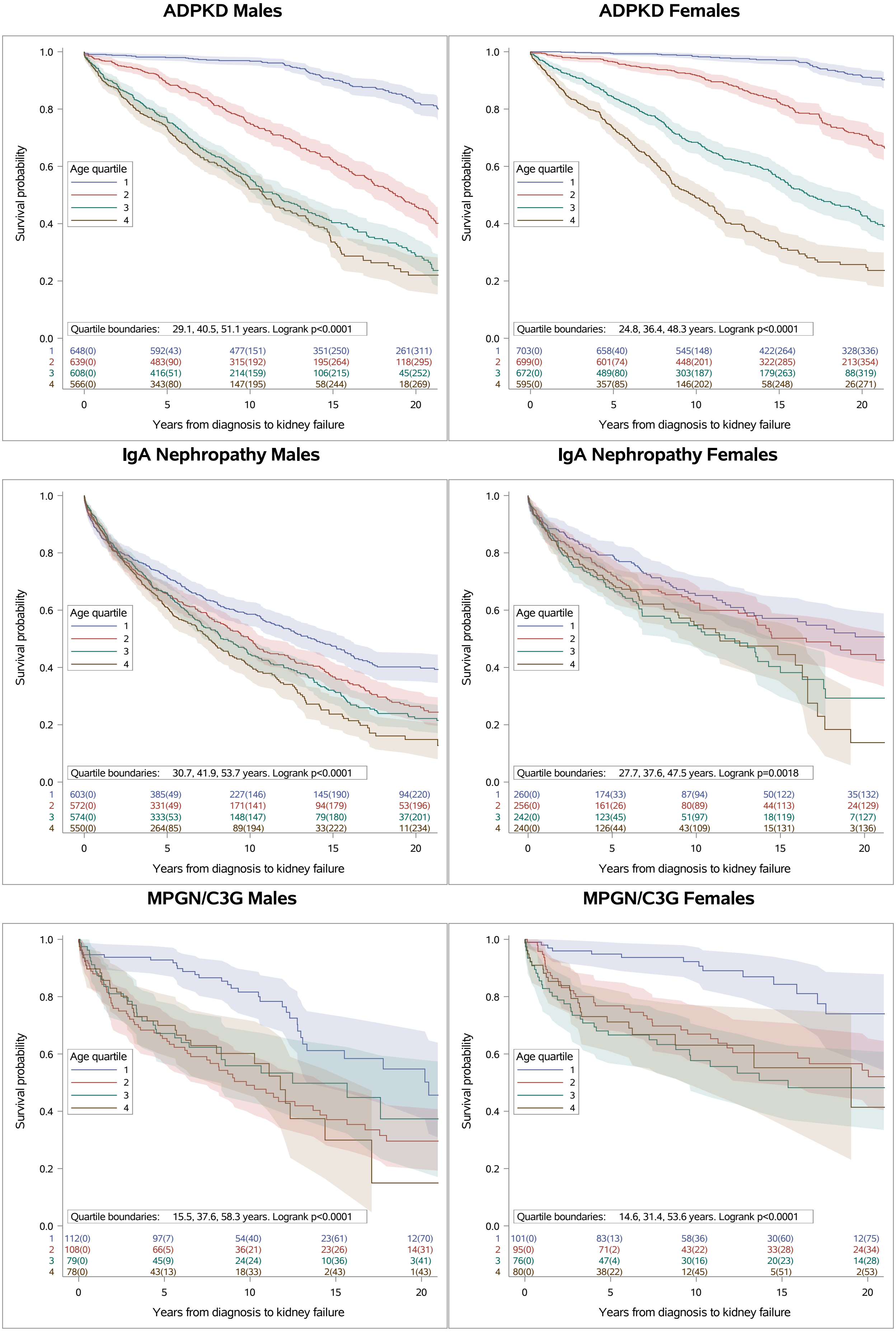

1= Youngest quartile, 4 = Oldest quartile

Supplementary Figure 11: Age at Kidney Failure for Males and Females, by RDG*

Numerical values represent "number at risk (number censored)".

ADPKD p-value= 0.061

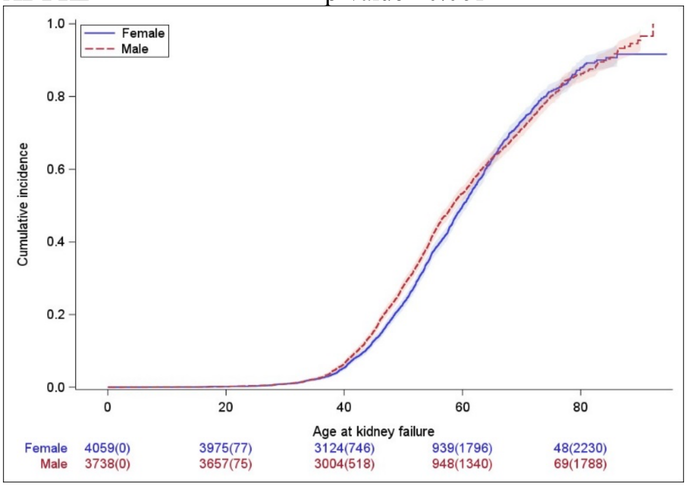

ADTKD p-value= 0.96

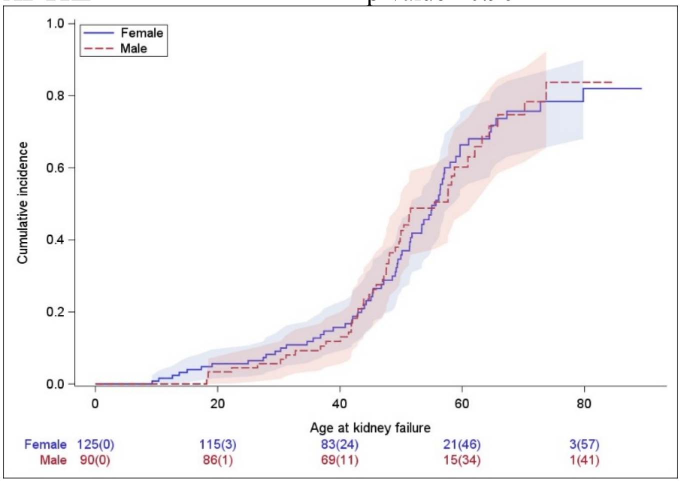

aHUS p-value= 0.94

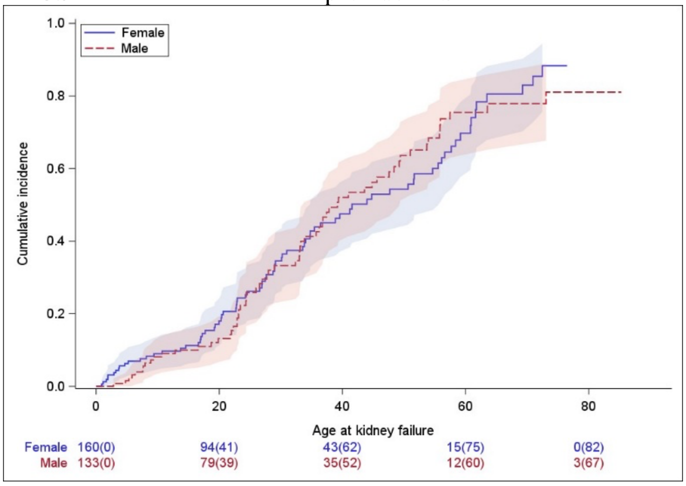

ANCA Vasculitis p-value= 0.44

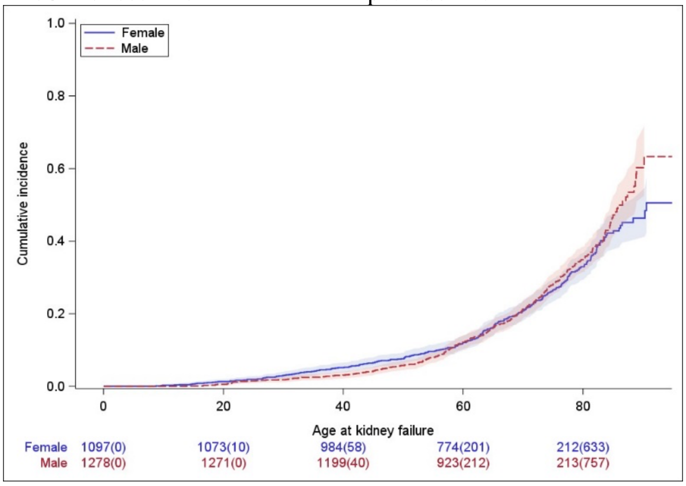

Anti-GBM disease p-value= 0.015

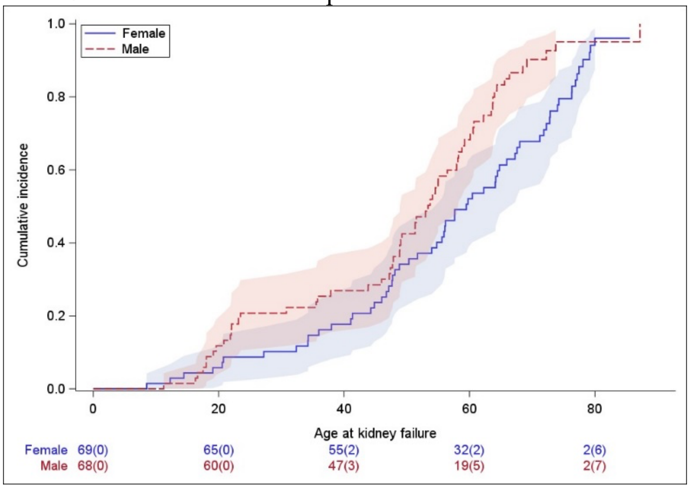

ARPKD/NPHP p-value= 0.91

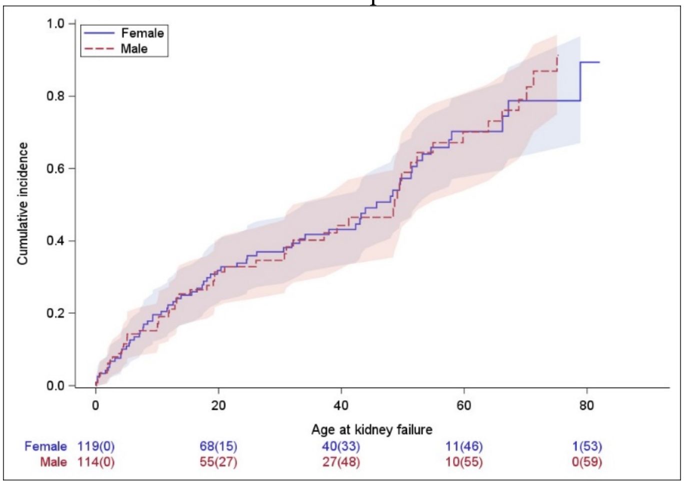

Cystinosis p-value= 0.66

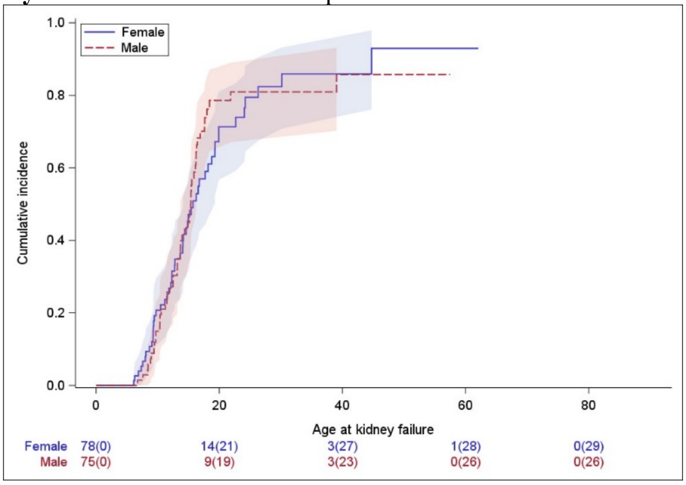

Cystinuria p-value= 0.88

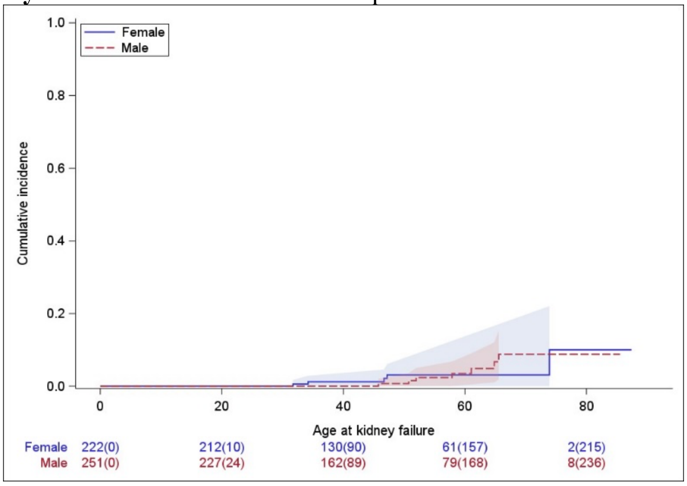

HNF1B mutations p-value= 1.00

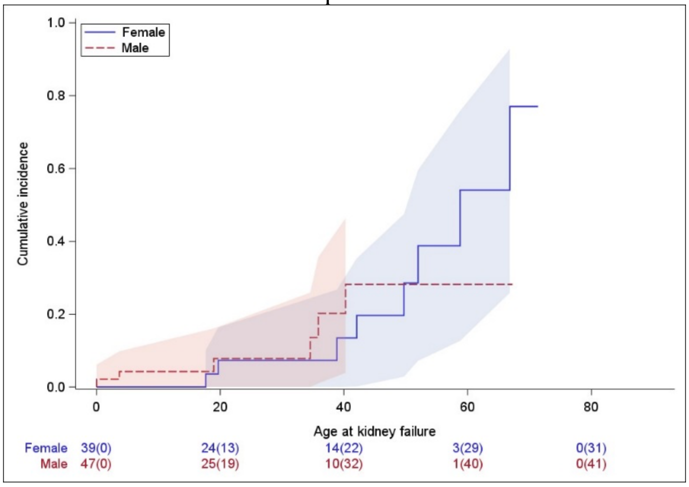

Hyperoxaluria p-value= 0.24

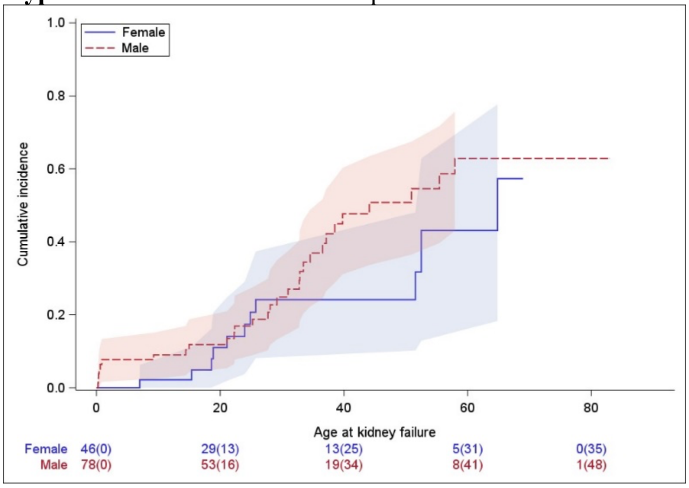

IgA nephropathy p-value= 0.34

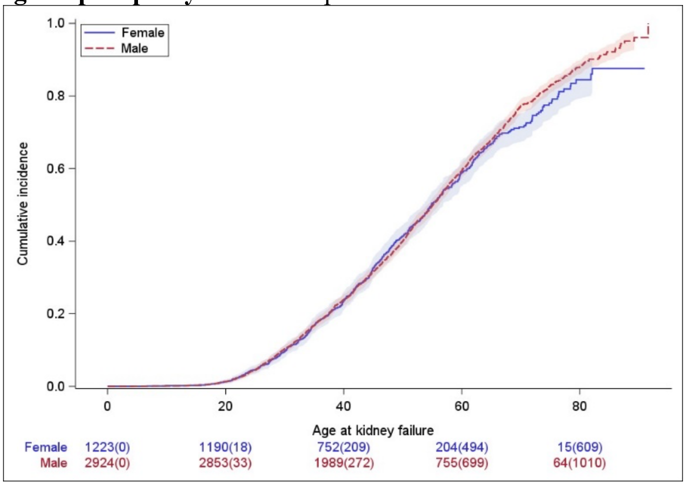

Membranous nephropathy p-value= 0.27

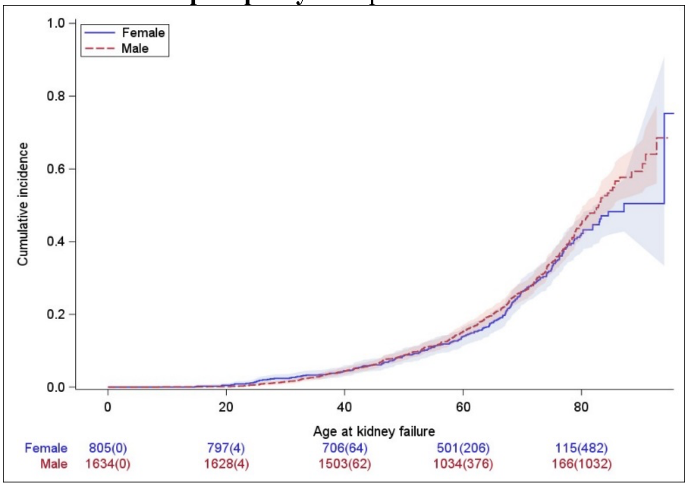

MGRS p-value= 0.31

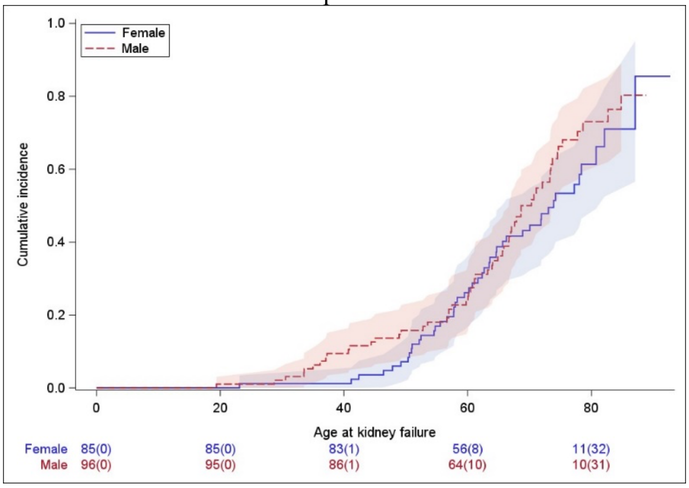

MPGN/C3G p-value= 0.14

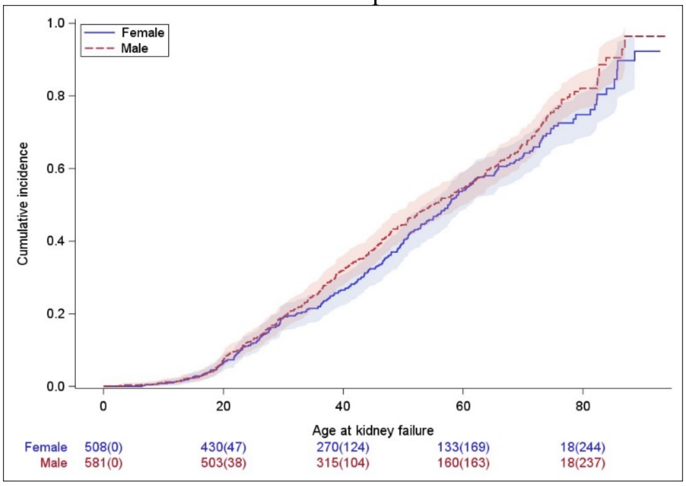

Other Tubulopathies p-value= 0.91

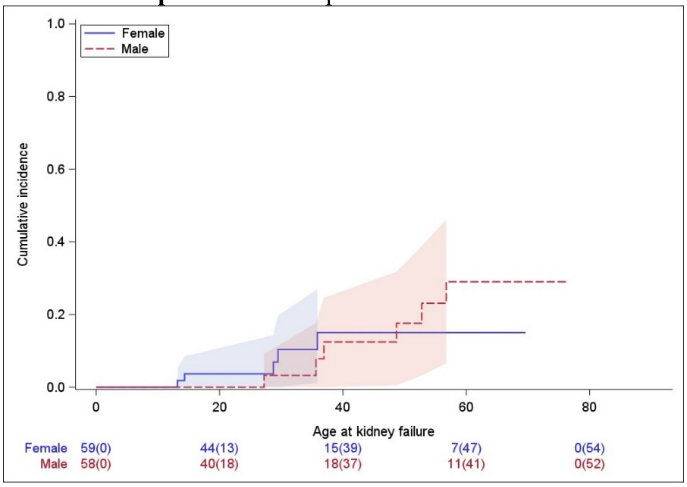

Other Vasculitides p-value= 0.76

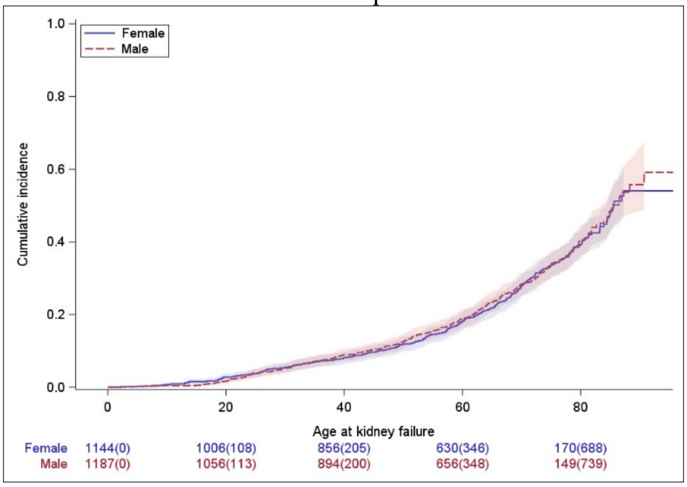

Retroperitoneal Fibrosis p-value= 0.28

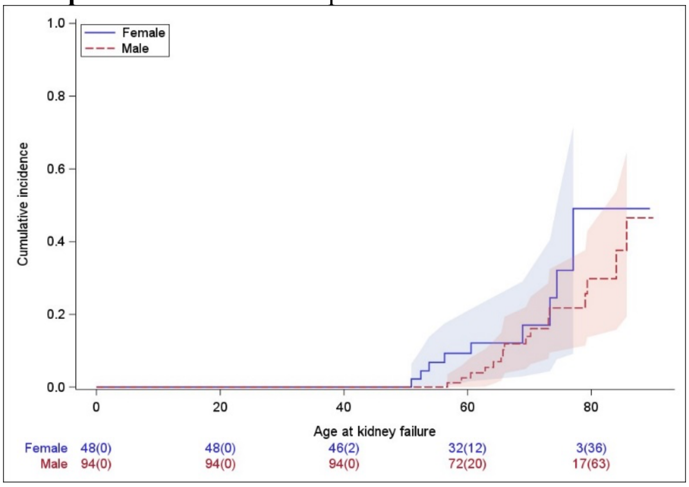

SRNS/FSGS p-value= 0.69

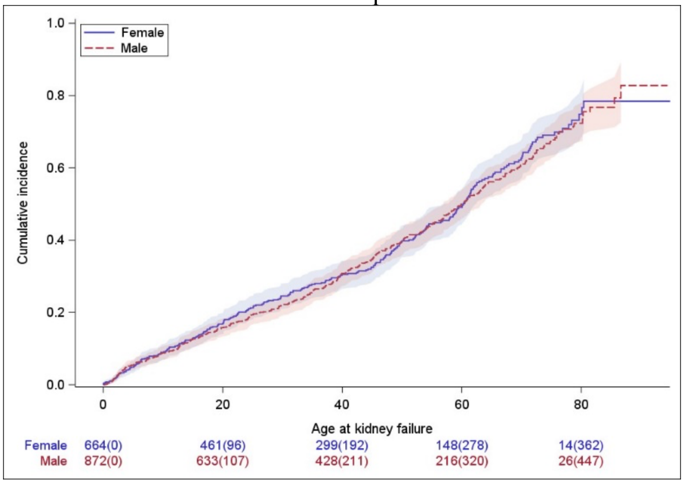

SSNS/MCD p-value= 0.72

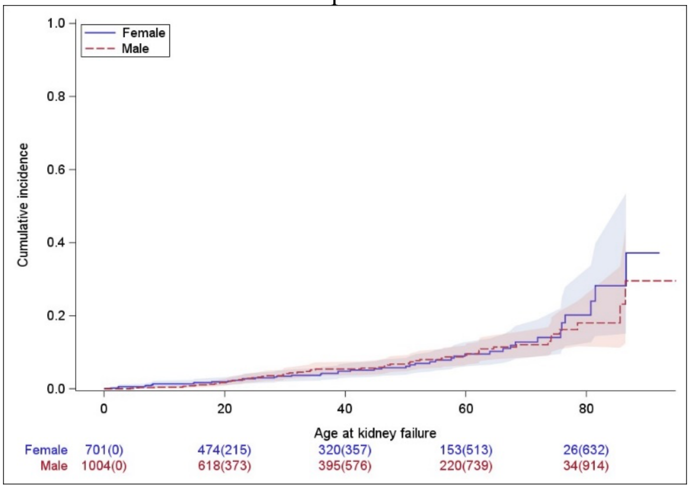

STEC-HUS p-value= 0.69

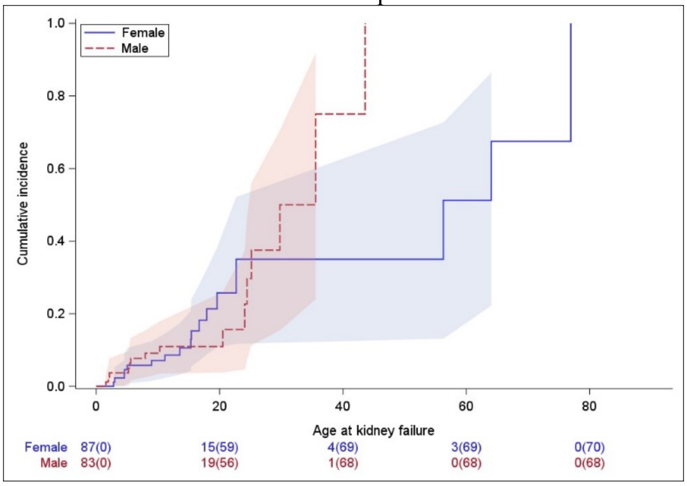

TBMN p-value= 0.29

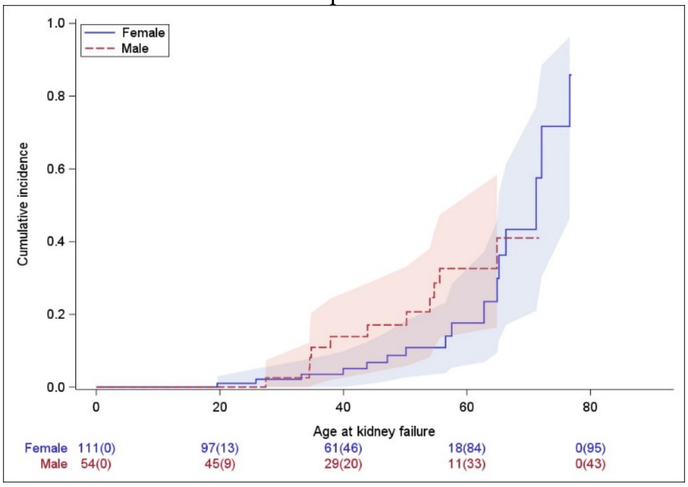

Tuberous Sclerosis Complex p-value= 0.26

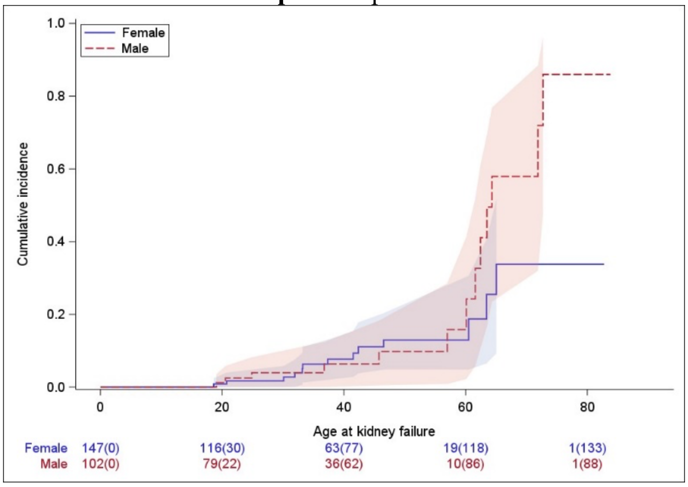

*X-linked Alport Syndrome presented stratified by Sex in Figure 1. Bartter Syndrome, Dent Disease and Lowe, Gitelman Syndrome and Inherited Renal Cancers are not presented due to <5 events across both Males and Females

Supplementary Figure 12: Sex stratified Kaplan Meier Survival analyses of years from dialysis start to death (not censored for transplant)

a) RaDaR patients (*Recruited*) b) Patients eligible for RaDaR in the UKRR but not yet recruited (*Eligible*) c) Patients with a Primary Renal Disease of Diabetes, Renovascular Disease or Hypertension (*Other PRD*). Numerical values represent “number at risk (number censored)”.

Males logrank* p<0.0001

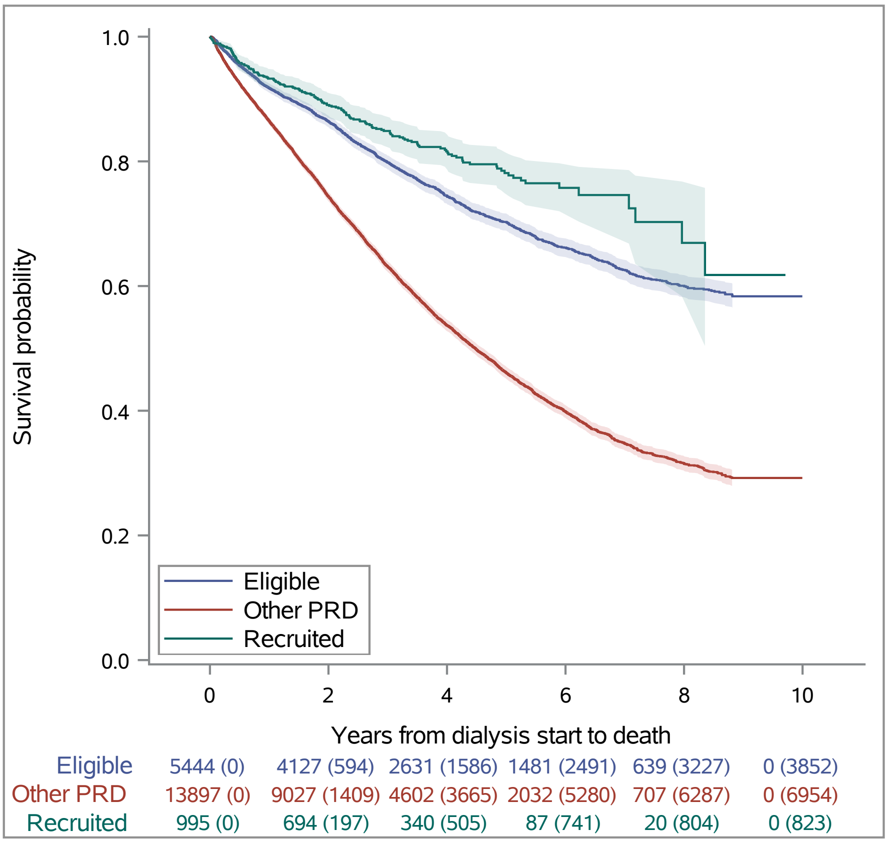

Females logrank* p<0.0001

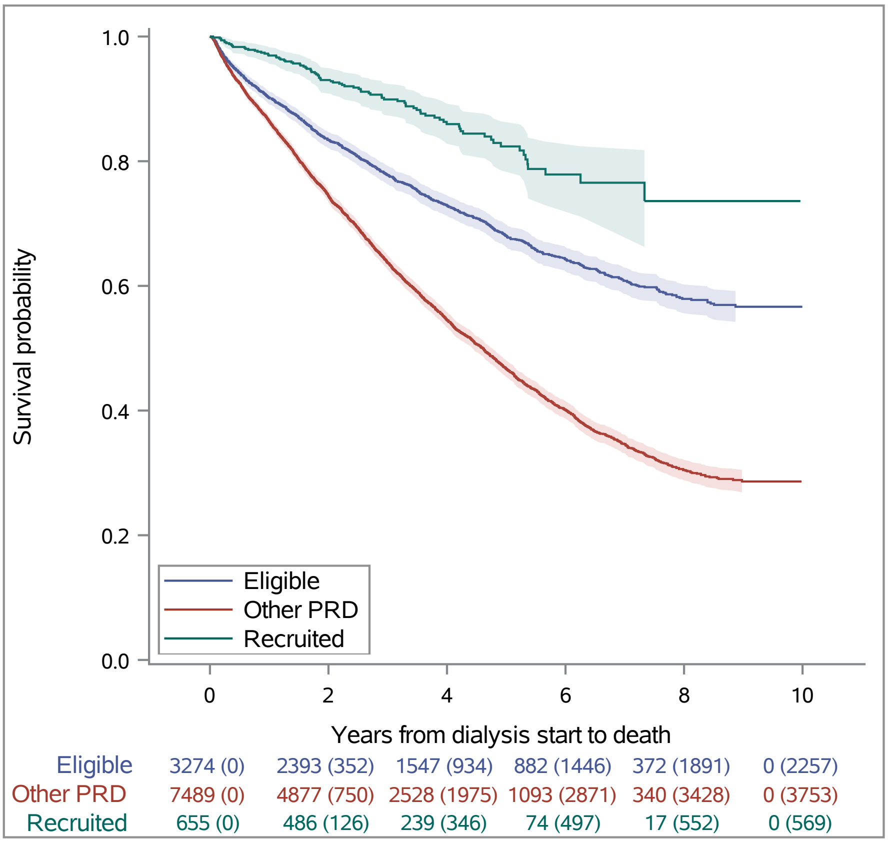

***Pairwise logrank statistic between RaDaR patients and patients with PRD of Diabetes, Hypertension or Renovascular Disease. PRD = Primary Renal Disease.**

Supplementary Table 6: Recruiting centres

Aberdeen	Hartlepool	St Helier’s
Airedale	Hereford	Stockport
Altnagelvin Hospital, Derry	Huddersfield	Stoke
Antrim	Hull	Sunderland
Ashford & St Peters	Inverness	Swansea
Basildon	Ipswich	Swindon
Bath	Kilmarnock	Tameside
Birkenhead Arrowe Park	Leeds Children’s	Taunton
Birmingham Children’s	Leeds St James'	Torbay
Birmingham Heartlands	Leicester	Truro Royal Cornwall Hospital
Birmingham Sandwell	Liverpool Aintree	Walsall Manor
Birmingham Queen Elizabeth	Liverpool Alder Hey	Warrington
Birmingham Women’s	Liverpool Royal	West Cumberland
Belfast City Hospitals	London - Barts	Wolverhampton
Belfast Ulster Hospital	London – Evelina & Guys	York
Blackburn	London - GOSH	
Bradford (St Luke’s)	London - Kings	
Brighton	London - Imperial	
Bristol Children's	London - Royal Free	
Bristol Southmead	London St George's	
Burton-on-Trent	Macclesfield	
Bury St Edmunds	Manchester Adults	
Cambridge	Manchester Children's	
Canterbury	Middlesbrough	
Cardiff	Newcastle	
Chelmsford (Broomfield)	North Tees	
Chester	Norwich	
Colchester	Nottingham Adults	
Coventry	Nottingham Children's	
Cumberland Infirmary	Oxford Churchill	
Daisy Hill, Newry	Oxford John Radcliffe	
Darlington	Peterborough	
Dartford	Plymouth	
Derby	Portsmouth	
Doncaster	Preston	
Dudley, Russells Hall	Queens Hospital, Romford	
Dumfries & Galloway	Reading	
Dundee	Salford	
Edinburgh	Sheffield	
Exeter	Shrewsbury & Telford	
Glasgow Children's	Southampton	
Glasgow Queen Elizabeth	Southend	
Gloucester	Stevenage	

RaDaR Inclusion and Exclusion Criteria

Supplementary Appendix 1: RaDaR Eligibility Criteria

Diagnosis	Cohort	Inclusion Criteria	Exclusion Criteria	Date of Diagnosis
**Adenine Phosphoribosyltransferase Deficiency (APRT-D)**	APRT Deficiency	APRT Deficiency confirmed<br><br>Abolished APRT enzyme activity or confirmed disease-causing mutation	None, if APRT Deficiency not confirmed	Date that clinical diagnosis was first made
**Alport Syndrome and Type IV collagenopathies**	Alport	Alport Syndrome definite or probable<br>Alport carrier definite or probable<br>Female heterozygote for X-linked Alport Syndrome (COL4A5)<br>Heterozygote for autosomal Alport Syndrome (COL4A3, COL4A4)<br>Thin basement membrane nephropathy	None stated	Date that clinical diagnosis was first made
**APOL1 disease, suspected or confirmed**	CKD-Africa Genes	People of African or Afro-Caribbean ancestry with CKD (KDIGO definition), >18 years including: Focal segmental glomerulosclerosis (primary or secondary) on renal biopsy; Non-diabetic and non-immunological kidney disease with no other confirmed cause	None stated	Date that clinical diagnosis was first made
**Autoimmune distal renal tubular acidosis**	Tubulopathy	Autoimmune distal renal tubular acidosis	None stated	Date that clinical diagnosis was first made
**Autosomal dominant distal renal tubular acidosis**	Tubulopathy	Autosomal dominant distal renal tubular acidosis<br>Genetically confirmed heterozygous pathogenic variant in SLC4A1	None stated	Date that clinical diagnosis was first made
**Autosomal recessive distal renal tubular acidosis**	Tubulopathy	Autosomal recessive distal renal tubular acidosis<br><br>Genetically confirmed homozygous pathogenic variant in ATP6V0A4, ATP6V1B1 or FOXI1	None stated	Date that clinical diagnosis was first made

RaDaR Inclusion and Exclusion Criteria

Diagnosis	Cohort	Inclusion Criteria	Exclusion Criteria	Date of Diagnosis
**Autosomal recessive proximal renal tubular acidosis**	Tubulopathy	Autosomal recessive proximal renal tubular acidosis with ocular abnormalities and intellectual disability<br><br>Genetically confirmed homozygous pathogenic variant in SLC4A4	None stated	Date that clinical diagnosis was first made

RaDaR Inclusion and Exclusion Criteria

Diagnosis	Cohort	Inclusion Criteria	Exclusion Criteria	Date of Diagnosis
**Bartter Syndrome types 1 and 2**	Tubulopathy	Bartter Syndrome, infantile onset<br><br>Hypokalaemic alkalosis, infantile onset without hypertension<br><br>Hypokalaemic alkalosis, infantile onset with raised renin	Acidosis<br><br>Persistent Hyperkalaemia	Date that clinical diagnosis was first made
**Bartter Syndrome type 3 Gitelman Syndrome**	Tubulopathy	Bartter Syndrome type 3<br><br>Gitelman Syndrome<br><br>Hypokalaemic alkalosis with hypomagnesaemia<br><br>Hypokalaemic alkalosis with raised renin<br><br>Hypokalaemic alkalosis without hypertension	Acidosis<br><br>Hyperkalaemia	Date that clinical diagnosis was first made
**Bartter Syndrome Type 4**	Tubulopathy	Bartter Syndrome, infantile onset with deafness<br><br>Hypokalaemic alkalosis, infantile onset without hypertension with deafness<br><br>Hypokalaemic alkalosis, infantile onset with raised renin, with deafness	Acidosis<br><br>Persistent Hyperkalaemia	Date that clinical diagnosis was first made
**BK Nephropathy**	BK Nephropathy	Significant BK viraemia, with polymerase chain reaction (PCR) greater than or equal to 10 log 4 copies per ml.<br><br>A confirmatory biopsy is **not** required.	None stated	Date that PCR first equalled or exceeded 10 log 4

RaDaR Inclusion and Exclusion Criteria

Diagnosis	Cohort	Inclusion Criteria	Exclusion Criteria	Date of Diagnosis
**Calciphylaxis**	Calciphylaxis	Any patient with a diagnosis of clinical diagnosis of Calciphylaxis; tissue diagnosis not required	None stated	Date that the diagnosis was made by a nephrologist or dermatologist
**Cystinosis (Nephropathic Cystinosis)**	Cystinosis	Cystinosis	None stated	Date that biochemical testing first showed an elevated level of white blood cell cysteine
**Cystinuria**	Cystinuria	Biochemically proven cystine kidney stone<br><br>Urinary cystine level > 3X reference range of the laboratory it was taken in<br><br>Cystine crystals in the urine (biochemically proven)	Another cause of proximal tubular dysfunction accounting for the raised cystine level e.g. Fanconi's syndrome	Date that any of the inclusion criteria first occurred
**Dent Disease**	Dent & Lowe	Dent Disease	None stated	Date that the clinical label of Dent Disease was first applied
**Dominant hypophosphatemia with nephrolithiasis or osteoporosis**	Tubulopathy	Dominant hypophosphatemia with nephrolithiasis or osteoporosis<br><br>Genetically confirmed heterozygous pathogenic variant in SLC34A1, SLC9A3R1, SLC34A3	None stated	Date that clinical diagnosis was first made
**Drug induced Fanconi syndrome**	Tubulopathy	Drug induced Fanconi syndrome	None stated	Date that clinical diagnosis was first made
**Drug induced hypomagnesemia**	Tubulopathy	Drug induced hypomagnesemia	None stated	Date that clinical diagnosis was first made

RaDaR Inclusion and Exclusion Criteria

Diagnosis	Cohort	Inclusion Criteria	Exclusion Criteria	Date of Diagnosis
**Drug induced Nephrogenic Diabetes Insipidus**	Tubulopathy	Drug induced Nephrogenic Diabetes Insipidus	None stated	Date that clinical diagnosis was first made
**EAST syndrome (Epilepsy, Ataxia, Sensorineural deafness, Tubulopathy)**	Tubulopathy	Gitelman/Bartter-type syndrome in childhood with epilepsy /ataxia	Normal CNS examination	Date that clinical diagnosis was first made
**End stage kidney disease of unknown cause**	CKD-Africa Genes	People of African or Afro-Caribbean ancestry with CKD (KDIGO definition), >18 years	Known cause of kidney disease identified (unless Sickle cell Nephropathy or APOL1 disease)	Date that clinical diagnosis was first made
**Fabry Disease**	Fabry	Confirmed diagnosis of Fabry Disease	None stated	Date that genetic diagnosis was made and/or, for males, the date that low alpha gal levels were first recorded
**Familial Hypomagnesaemia with hypercalciuria and nephrocalcinosis CLDN16/19**	Tubulopathy	Familial Hypomagnesaemia with Hypercalciuria and Nephrocalcinosis<br><br>Genetically confirmed homozygous pathogenic variant in CLDN 16/19	None stated	Date that clinical diagnosis was first made
**Familial primary hypomagnesemia with hypocalcuria FXYD2**	Tubulopathy	Familial primary hypomagnesemia with hypocalciuria<br>Genetically confirmed homozygous pathogenic variant in FXYD2	None stated	Date that clinical diagnosis was first made
**Familial primary hypomagnesemia with normocalcuria EGF**	Tubulopathy	Familial primary hypomagnesemia with normocalcuria<br><br>Genetically confirmed homozygous pathogenic variant in EGF	None stated	Date that clinical diagnosis was first made
**Familial renal glucosuria**	Tubulopathy	Familial renal glucosuria	None stated	Date that clinical diagnosis

RaDaR Inclusion and Exclusion Criteria

Diagnosis	Cohort	Inclusion Criteria	Exclusion Criteria	Date of Diagnosis
**SLC5A2**		Genetically confirmed homozygous pathogenic variant in SLC5A2		was first made

RaDaR Inclusion and Exclusion Criteria

Diagnosis	Cohort	Inclusion Criteria	Exclusion Criteria	Date of Diagnosis
**Fanconi Renotubular syndrome 1 (FRTS1)**	Tubulopathy	Fanconi Renotubular syndrome 1	None stated	Date that clinical diagnosis was first made
**Fanconi Renotubular syndrome 2 (FRTS2)**	Tubulopathy	Fanconi Renotubular syndrome 2<br><br>Genetically confirmed homozygous pathogenic variant in SLC34A1	None stated	Date that clinical diagnosis was first made
**Fanconi Renotubular syndrome 3 (FRTS3)**	Tubulopathy	Fanconi Renotubular syndrome 3<br><br>Genetically confirmed homozygous pathogenic variant in EHHADH	None stated	Date that clinical diagnosis was first made
**Fibromuscular Dysplasia**	Fibromuscular Dysplasia	Diagnosis of FMD established on radiological or histological grounds<br><br>FMD of any arterial bed	None stated	Date that FMD was diagnosed by radiological (or histological) methods
**Generalized pseudohypoaldosteronism type 1**	Tubulopathy	Generalized pseudohypoaldosteronism type 1<br><br>Genetically confirmed homozygous pathogenic variant in SCNN1A/ SCNN1B/SCNN1G	None stated	Date that clinical diagnosis was first made

RaDaR Inclusion and Exclusion Criteria

Diagnosis	Cohort	Inclusion Criteria	Exclusion Criteria	Date of Diagnosis
**Haemolytic Uraemic Syndrome - Atypical**	aHUS	Diarrhoea-negative HUS, includes congenital and familial HUS<br><br>Renal biopsy showing a TMA and/or the triad of microangiopathic haemolytic anaemia, thrombocytopenia, renal failure.	Shiga toxin associated HUS<br><br>Secondary causes:<br>• Drugs<br>• Infection (HIV, pneumonia, streptococcus)<br>• Transplantation (bone marrow, liver, lung, cardiac but not de-novo renal)<br>• Cobalamin deficiency<br>• SLE<br>• APL Ab syndrome<br>• Scleroderma<br>• ADAMTS13 antibodies or deficiency	Date of first presentation

RaDaR Inclusion and Exclusion Criteria

Diagnosis	Cohort	Inclusion Criteria	Exclusion Criteria	Date of Diagnosis
**Haemolytic Uraemic Syndrome-Shiga toxin (Verocytotoxin)-associated**	STEC-HUS	Acute kidney injury (AKI) with elevated creatinine for age and/or oligoanuria (urine output <0.5ml/kg/hr over 24hr period) with either:<br>• Microangiopathic haemolytic anaemia (MAHA) - defined as Hgb < 10mg/dl with fragmented RBCs<br>or<br>• Thrombocytopaenia - defined as platelet count less than 130, 000 x 10 9/l<br>and<br>• Occurring with Shiga-toxin producing E Coli (STEC) infection defined as:<br>• Positive STEC culture<br>• Positive PCR for Stx gene directly from a faecal specimen<br>• Positive antibodies to the lipopolysaccharide<br>• antigen of E. coli serogroups O157, O26, O103, O111 and O145	Septicaemia<br>Malignant hypertension<br>Primary vascular disease<br>Familial HUS not being part of the same	Date on which the STEC-HUS was suspected.
**Heavy metal induced Fanconi syndrome**	Tubulopathy	Heavy metal induced Fanconi syndrome	None stated	Date that clinical diagnosis was first made

RaDaR Inclusion and Exclusion Criteria

Diagnosis	Cohort	Inclusion Criteria	Exclusion Criteria	Date of Diagnosis
**Hepatocyte Nuclear Factor-1B mutation**	HNF1b	Hepatocyte nuclear factor-1B mutation<br><br>Renal cysts and diabetes (RCAD)<br><br>Inherited genetic diabetes type 2 (MODY 5).	None stated	Date of genetic diagnosis
**Hereditary renal hypouricemia**	Tubulopathy	Hereditary renal hypouricemia<br><br>Genetically confirmed homozygous pathogenic variant in SLC22A12, SLC2A9	None stated	Date of genetic diagnosis
**Hereditary hypophosphatemic rickets with hypercalciuria**	Tubulopathy	Hereditary hypophosphatemic rickets with hypercalciuria<br><br>Genetically confirmed homozygous pathogenic variant in SLC34A3	None stated	Date of genetic diagnosis
**Hyperoxaluria (Primary hyperoxaluria, Oxalosis)**	Hyperoxaluria	Primary Hyperoxaluria Type1<br><br>Primary Hyperoxaluria Type 2<br><br>Primary Hyperoxaluria Type 3<br><br>Primary Hyperoxaluria awaiting genetic confirmation (Urine oxalate excretion ≥ 0.8 mmol/1.73 m^2/24 hrs)<br><br>Primary Hyperoxaluria Unclassified<br><br>Primary Hyperoxaluria Unclassified but with systemic oxalate deposition	Secondary hyperoxaluria associated with gastrointestinal disease<br><br>Renal failure without systemic oxalate deposits	Date that definitive diagnosis by genetic confirmation with gene mutation was first made.<br><br>If in doubt use the earliest date that PH was suspected or the date when treatment was first introduced

RaDaR Inclusion and Exclusion Criteria

Diagnosis	Cohort	Inclusion Criteria	Exclusion Criteria	Date of Diagnosis
**Hypertensive kidney disease**	CKD-Africa Genes	People of African or Afro-Caribbean ancestry with CKD (KDIGO definition), >18 years	Known cause of Kidney disease	Date that clinical diagnosis was first made
**Hyperuricaemic Nephropathy (Primary/Familial Hyperuricaemic nephropathy)**<br><br>**Medullary cystic kidney disease**	ADTKD	Autosomal Dominant Tubulointerstitial Kidney Disease (ADTKD; previously known as FUAN)<br><br>Familial juvenile hyperuricaemic nephropathy<br><br>Familial gouty nephropathy<br><br>Familial urate nephropathy<br><br>Familial interstitial nephropathy<br><br>Uromodulin-associated nephropathy<br><br>Medullary cystic kidney disease (type I or II)	None stated	Date that genetic confirmation was received
**IgA Nephropathy**	IgA Nephropathy	Biopsy proven IgA Nephropathy plus proteinuria >0.5g/ day or eGFR<60ml/min	All forms of secondary IgA nephropathy, including Henoch Schonlein purpura	Date of renal biopsy
**Isolated autosomal dominant hypomagnesemia, Glaudemans type**	Tubulopathy	Isolated autosomal dominant hypomagnesemia<br><br>Genetically confirmed homozygous pathogenic variant in KCNA1	None stated	Date that clinical diagnosis was first made

RaDaR Inclusion and Exclusion Criteria

Diagnosis	Cohort	Inclusion Criteria	Exclusion Criteria	Date of Diagnosis
**Liddle syndrome**	Tubulopathy	Liddle syndrome<br><br>Hypertension with hypokalaemia, suppressed aldosterone<br><br>Hypertension with suppressed aldosterone<br><br>Autosomal dominant hypertension, suppressed aldosterone	Hyperaldosteronism	Date that clinical diagnosis was first made
**Lowe Syndrome**	Dent & Lowe	Lowe Syndrome	None Stated	Date that the clinical label of Lowe Syndrome was first applied

RaDaR Inclusion and Exclusion Criteria

Diagnosis	Cohort	Inclusion Criteria	Exclusion Criteria	Date of Diagnosis
**Membranoproliferative glomerulonephritis**<br><br>**Mesangiocapillary glomerulonephritis**<br><br>**Dense Deposit Disease**<br><br>**C3 Glomerulonephritis**<br><br>**C3 Glomerulopathy**	MPGN	Child or adult with histological finding of:<br><br>MPGN Type I<br><br>Dense Deposit Disease (morphological pattern may or may not be MPGN)<br><br>Other pattern of MPGN<br><br>C3 Glomerulonephritis (Characterised by C3 deposits in the absence of immunoglobulin with electron dense deposits (morphological pattern may or may not be MPGN)<br><br>Unclassified GN with capillary wall immune deposits	MPGN known to be secondary to:<br><br>Chronic bacterial infection<br><br>Hepatitis B or C infection<br><br>Malignancy<br><br>Systemic lupus erythematosus (by ACR criteria)	Date of biopsy
**Membranous Nephropathy**	Membranous Nephropathy	Membranous nephropathy confirmed by kidney histology	Lupus nephritis	Date of biopsy
**Mitochondrial Renal Disease**	Mitochondrial	Mitochondrial Disease **or** Mitochondrial Cytopathy	None Stated	Date that clinical diagnosis was first made

RaDaR Inclusion and Exclusion Criteria

Diagnosis	Cohort	Inclusion Criteria	Exclusion Criteria	Date of Diagnosis
**Monoclonal Gammopathy of Renal Significance**	MGRS	Renal biopsy proven confirmation of:<br><br>• AH amyloidosis*<br>• AHL amyloidosis*<br>• AL amyloidosis*<br>• C3 glomerulonephritis with monoclonal gammopathy<br>• Crystalglobulinaemia<br>• Crystal-storing histiocytosis<br>• Fibrillary Glomerulonephritis<br>• Immunotactoid/Glomerulonephritis with Organised Microtubular Monoclonal Immunoglobulin Deposits (GOMMID)<br>• Intracapillary monoclonal IgM without cryoglobulin<br>• Intraglomerular/capillary lymphoma/leukaemia<br>• Light chain cast nephropathy<br>• Light chain proximal tubulopathy, crystalline<br>• Light chain proximal tubulopathy, non crystalline<br>• Monoclonal Immunoglobulin Deposition Disease (MIDD; includes Light Chain Deposition Disease - LCDD; Heavy Chair Deposition Disease - HCDD; and Light and Heavy Chain Deposition Disease - LHCDD)<br>• Proliferative glomerulonephritis with monoclonal immunoglobulin deposits – PGNMID<br>• Thrombotic Microangiopathy with monoclonal gammopathy<br>• Type 1 cryoglobulinaemic Glomerulonephritis<br>• Unclassified MGRS<br><br>*Patients with systemic amyloidosis may have a renal biopsy confirming AL amyloidosis or a biopsy of other tissue with confirmation of renal involvement by the UK National Amyloidosis Centre.	None Stated	Date of biopsy

RaDaR Inclusion and Exclusion Criteria

Diagnosis	Cohort	Inclusion Criteria	Exclusion Criteria	Date of Diagnosis
**Nephrogenic diabetes insipidus**	Tubulopathy	Nephrogenic diabetes insipidus<br><br>Genetically confirmed homozygous pathogenic variant in AVPR2, AQP2	None stated	Date that clinical diagnosis was first made
**Nephrogenic syndrome of inappropriate antidiuresis**	Tubulopathy	Nephrogenic syndrome of inappropriate antidiuresis<br><br>Genetically confirmed homozygous pathogenic variant in AVPR2	None stated	Date that clinical diagnosis was first made
**Nephronophthisis**	ARPKD/NPHP	Histological or radiological features of Nephronophthisis<br><br>Genetic diagnosis of Nephronophthisis or Nephronophthisis-related ciliopathy	None stated	Date that histological /radiological or genetic diagnosis was first made

RaDaR Inclusion and Exclusion Criteria

Diagnosis	Cohort	Inclusion Criteria	Exclusion Criteria	Date of Diagnosis
**Nephrotic Syndrome - Steroid Sensitive or Steroid Resistant**<br><br>**(Congenital nephrotic syndrome, nephrotic syndr ome with focal segmental glomerulosclerosis)**	INS	Children and adults with idiopathic Nephrotic Syndrome (nephrotic range proteinuria and hypoalbuminaemia)<br><br>Congenital NS (presumed Steroid Resistance)<br><br>Childhood or adult onset with primary Steroid Resistance<br><br>Childhood or adult onset with late onset Steroid Resistance<br><br>Steroid Sensitive Nephrotic Syndrome (full or partial remission in response to steroids)<br><br>As part of a syndrome e.g. Nail Patella Syndrome and Denys-Drash Syndrome<br><br>Those with a biopsy diagnosis of FSGS or minimal change disease can be included if they fall in the above categories but biopsy is not a prerequisite for inclusion	Secondary causes of Nephrotic Syndrome<br><br>• Primary diagnosis of Glomerulonephritis (IgA Nephropathy, Membranoproliferative Glomerulonephritis, Membranous Nephropathy)<br>• Vasculitis<br>• Systemic Lupus Erythematosus<br>• Diabetes<br>• Obesity<br>• Hypertension	Date of presentation to secondary or tertiary centre
**Oncogenic osteomalacia**	Tubulopathy	Oncogenic osteomalacia	None stated	Date that clinical diagnosis was first made
**Osteopetrosis with renal tubular acidosis**	Tubulopathy	Osteopetrosis with renal tubular acidosis<br><br>Genetically confirmed homozygous pathogenic variant in CA2	None stated	Date that clinical diagnosis was first made

Diagnosis	Cohort	Inclusion Criteria	Exclusion Criteria	Date of Diagnosis
**Polycystic Kidney Disease - Autosomal Dominant**	ADPKD	Clinical features of Autosomal Dominant Polycystic Kidney Disease meeting current image based diagnostic criteria<br><br>Clinical features compatible with ADPKD in the absence of a family history<br><br>Pathogenic or likely pathogenic PKD1 or PKD2 mutation with or without clinical features	Autosomal dominant polycystic liver disease with no evidence of renal cysts	Date that the clinical diagnosis was first made.<br><br>This may be reported by the clinician as the date of the diagnostic scan or by the patient if scans were performed at another centre
**Polycystic Kidney Disease - Autosomal Recessive**	ARPKD	Autosomal Recessive Polycystic Kidney Disease<br><br>Congenital Hepatic Fibrosis<br><br>Caroli Syndrome with kidney malformation or cyst	None stated	Date that clinical diagnosis was first made.

Diagnosis	Cohort	Inclusion Criteria	Exclusion Criteria	Date of Diagnosis
**Pregnancy and Chronic Kidney Disease**	Pregnancy	Pregnancy in all women known to have CKD 1-5 prior to pregnancy or those with a serum creatinine >85umol/l on two occasions during pregnancy<br><br>Pregnancy in all women with renal transplants regardless of function<br><br>Pregnancy in all women with previous or current lupus nephritis regardless of function	None stated	Date of last menstrual period
**Primary hypomagnesemia with secondary hypocalcemia**	Tubulopathy	Primary hypomagnesemia with secondary hypocalcemia<br><br>Genetically confirmed homozygous pathogenic variant in TRPM6	None stated	Date that clinical diagnosis was first made
**Pseudohypoaldosteronism type 2A**	Tubulopathy	Pseudohypoaldosteronism type 2A	None stated	Date that clinical diagnosis was first made
**Pseudohypoaldosteronism type 2B**	Tubulopathy	Pseudohypoaldosteronism type 2B<br><br>Genetically confirmed homozygous pathogenic variant in WNK1	None stated	Date that clinical diagnosis was first made
**Pseudohypoaldosteronism type 2C**	Tubulopathy	Pseudohypoaldosteronism type 2C<br><br>Genetically confirmed homozygous pathogenic variant in WNK4	None stated	Date that clinical diagnosis was first made
**Pseudohypoaldosteronism type 2D**	Tubulopathy	Pseudohypoaldosteronism type 2D<br><br>Genetically confirmed homozygous pathogenic variant in KLHL3	None stated	Date that clinical diagnosis was first made

Diagnosis	Cohort	Inclusion Criteria	Exclusion Criteria	Date of Diagnosis
**Pseudohypoaldosteronism type 2E**	Tubulopathy	Pseudohypoaldosteronism type 2E<br><br>Genetically confirmed homozygous pathogenic variant in CUL3	None stated	Date that clinical diagnosis was first made
**Pure Red Cell Aplasia**	PRCA	Treatment with any injectable form of erythropoiesis stimulating agent for at least four weeks.<br><br>Haemoglobin <70 g/l without transfusion or transfusion dependence.<br><br>Normal leucocyte and platelet count<br><br>Reticulocyte count < 20.000 / mm3<br><br>Bone marrow aspirate showing well preserved myeloid and megakaryocyte development, and <5% erythroblasts.<br><br>Presence of anti-erythropoietin antibodies.	Pre-established PRCA due to myeloproliferative disorder	Date of positive antibody test
**Renal pseudohypoaldosteronism type 1**	Tubulopathy	Renal pseudohypoaldosteronism type 1<br><br>Genetically confirmed homozygous pathogenic variant in NR3C2		Date that clinical diagnosis was first made

Diagnosis	Cohort	Inclusion Criteria	Exclusion Criteria	Date of Diagnosis
**Retroperitoneal Fibrosis**	Retroperitoneal Fibrosis	Any radiologically confirmed retroperitoneal fibrosis (RPF), presumed to be 'idiopathic' or associated with primary conditions including (but not exclusively):<br>• Aortitis<br>• Periaortitis<br>• IgG4-related Vasculitis<br>• Perivascular fibrosis<br>• Atherosclerotic or aneurysmal disease<br><br>**Note:** There is no specific ICD code for retroperitoneal fibrosis although the diagnosis term links to two ICD codes:<br>• ICD10:N13.5 - Crossing vessel and stricture of ureter without hydronephrosis<br>• ICD-9-CM 593.4 - Other ureteric obstruction	Neoplastic disease within retroperitoneal fibrosis mass defined histologically	Date of diagnostic imaging study report
**Sickle Cell Nephropathy**	**CKD Africa Genes**	People of African or Afro-Caribbean ancestry with CKD (KDIGO definition), >18 years<br><br>Known Sickle Cell disease with reduced kidney function, and/or blood or protein in urine with no other cause for kidney disease identified	None stated	Date that clinical diagnosis was first made

**Tuberous Sclerosis**	**Tuberous Sclerosis**	Clinical or molecular diagnosis of Tuberous Sclerosis Complex (TSC)<br><br>Multiple renal angiomyolipomas<br><br>Multiple renal angiomyolipomas (> 3) +/- pulmonary lymphangioleiomyomatosis (LAM) without other signs of TSC	None stated	Date that clinical diagnosis was first made

**Vasculitis (Primary systemic Vasculitis)**	**Vasculitis**	**Small vessel Vasculitis (ANCA associated)**<br><br>Microscopic polyangiitis (including renal limited Vasculitis)<br><br>Granulomatosis with polyangiitis (Wegener)<br><br>Eosinophilic granulomatosis with polyangiitis (Churg Strauss)<br><br>ANCA Vasculitis unclassified<br><br>**Small vessel Vasculitis (Immune complex)**<br><br>anti-GBM disease<br><br>Cryoglobulinemic Vasculitis<br><br>IgA Vasculitis (Henoch-Schönlein)<br><br>**Medium vessel Vasculitis**<br><br>Classical PAN<br><br>Kawasaki disease<br><br>**Large vessel Vasculitis**<br><br>Giant cell arteritis<br><br>Takayasu's arteritis	None stated	Date of biopsy.<br><br>In the absence of a biopsy, the date of a positive antibody test should be used

**Vasculitis (Primary systemic Vasculitis)**	Vasculitis	**Variable vessel Vasculitis**<br><br>Behçet’s disease<br><br>Cogan’s syndrome<br><br>**Single organ Vasculitis**<br><br>Isolated aortitis<br><br>Primary cerebral angiitis	None stated	Date of biopsy.<br><br>In the absence of a biopsy, the date of a positive antibody test should be used

**Inherited Renal Cancer Syndrome**	Renal Cancer Inherited	1. A molecular or clinical diagnosis according to standard criteria of any of the following conditions:<br><br>Von Hippel Lindau disease (VHL) OMIM 193300<br><br>PTEN hamartoma tumour syndrome (Cowden syndrome) OMIM 158350<br><br>Birt Hogg Dube syndrome (BHD) OMIM 135150<br><br>Hereditary leiomyomatosis and renal cell cancer syndrome(HLRCC) OMIM 150800<br><br>Succinate dehydrogenase-related tumour predisposition syndrome<br><br>BAP1-related tumour predisposition syndrome OMIM 614327<br><br>Hereditary Type 1 papillary renal cell carcinoma syndrome (MET oncogene) OMIM 605074<br><br>2. Two or more cases in first degree relatives of any type of renal cancer without an established molecular or clinical diagnosis<br>**3.** Bilateral, multiple primary renal cancers of any histopathological type with or with a family history	None stated	Date of molecular or clinical diagnosis according to standard criteria

Supplementary Appendix 2: National Registry of Rare Kidney Diseases (RaDaR) Consortium

Sharirose Abat[1], Shazia Adalat[2], Joy Agbonmwandolor[3], Zubaidah Ahmad[4], Abdulfattah Alejmi[5], Rashid Almasarwah[6], Nicholas Annear[1], Ellie Asgari[4], Amanda Ayers[7], Jyoti Baharani[8], Gowrie Balasubramaniam[9], Felix Jo-Bamba Kpodo[10] , Tarun Bansal[11], Alison Barratt[12], Jonathan Barratt[79], Megan Bates[13], Natalie Bayne[14], Janet Bendle[15], Sarah Benyon[16], Carsten Bergmann[17, 18], Sunil Bhandari[19], Coralie Bingham[20], Preetham Boddana[21], Sally Bond[22], Fiona Braddon[23], Kate Bramham[23], Angela Branson[15], Stephen Brearey[24], Vicky Brocklebank[25], Sharanjit Budwal[26], Conor Byrne[27], Hugh Cairns[28], Brian Camilleri[29], Gary Campbell[30], Alys Capell[31], Margaret Carmody[8], Marion Carson[32], Tracy Cathcart[19], Christine Catley[9], Karine Cesar[33], Melanie Chan[6], Houda Chea[15], James Chess[34], Chee Kay Cheung[26], Katy-Jane Chick[35], Nihil Chitalia[36], Martin Christian[37], Tina Chrysochou[38, 39], Katherine Clark[40], Christopher Clayton[41], Rhian Clissold[20], Helen Cockerill[33], Joshua Coelho[42], Elizabeth Colby[43], Viv Colclough[44], Eileen Conway[45], H. Terence Cook[46], Wendy Cook[47], Theresa Cooper[48], Richard J Coward[43], Sarah Crosbie[22], Gabor Cserep[49], Anjali Date[50], Katherine Davidson[48], Amanda Davies[51], Neeraj Dhaun[52], Ajay Dhaygude[53], Lynn Diskin[12], Abhijit Dixit[41, 54], Eunice Ann Doctolero[35], Suzannah Dorey[55], Lewis Downard[23], Mark Drayson[56], Gavin Dreyer[27], Tina Dutt[57], Kufreabasi Etuk[28], Dawn Evans[58], Jenny Finch[29], Frances Flinter[59], James Fotheringham[60], Lucy Francis[82], Daniel P. Gale[61], Hugh Gallagher[62], David Game[4], Eva Lozano Garcia[42], Madita Gavrila[22], Susie Gear[63], Colin Geddes[64], Mark Gilchrist[65], Matt Gittus[66], Paraskevi Goggolidou[67], Christopher Goldsmith[57], Patricia Gooden[68], Andrea Goodlife[26], Priyanka Goodwin[53], Tassos Grammatikopoulos[28, 69], Barry Gray[70], Megan Griffith[46], Steph Gumus[9], Sanjana Gupta[71], Patrick Hamilton[72], Lorraine Harper[56], Tess Harris[73], Louise Haskell[74], Samantha Hayward[43], Shivaram Hegde[75], Bruce Hendry[76], Sue Hewins[77], Nicola Hewitson[78], Kate Hillman[15], Mrityunjay Hiremath[57], Alexandra Howson[79], Zay Htet[28], Sharon Huish[16], Richard Hull[1], Alister Humphries[68], David P. J. Hunt[119], Karl Hunter[80], Samantha Hunter[19], Marilyn Ijeomah-Orji[6], Nick Inston[81], David Jayne[82], Gbemisola Jenfa[31], Alison Jenkins[83], Sally Johnson[118], Caroline A Jones[84], Colin Jones[85], Amanda Jones[5], Rachel Jones[82], Lavanya Kamesh[81], Durga Kanigicherla[39], Fiona Karet Frankl[82], Mahzuz Karim[86], Amrit Kaur[87], David Kavanagh[25], Kelly Kearley[88], Larissa Kerecuk[14], Arif Khwaja[70], Garry King[23], Grant King[89], Ewa Kislowska[4], Edyta Klata[29], Maria Kokocinska[14], Mark Lambie[90], Laura Lawless[41], Thomas Ledson[80], Rachel Lennon[91], Adam P Levine[92], Ling Wai Maggie Lai[16], Graham Lipkin[81], Graham Lovitt[93], Paul Lyons[94], Holly Mabillard[95], Katherine Mackintosh[7], Khalid Mahdi[96], Eamonn Maher[97], Kevin J. Marchbank[25], Patrick B Mark[64], Sherry Masoud[23], Bridgett Masunda[9], Zainab Mavani[31], Jake Mayfair[4], Stephen McAdoo[6], Joanna Mckinnell[98], Nabil Melhem[2], Simon Meyrick[51], Shabbir Moochhala[61], Putnam Morgan[99], Ann Morgan[100, 101] , Fawad Muhammad[5], Shona Murray[30], Kristina Novobritskaya[22], Albert CM Ong[66, 70], Louise Oni[102], Kate Osmaston[23], Neal Padmanabhan[64], Sharon Parkes[14], Jean Patrick[7], James Pattison[4], Riny Paul[1], Rachel Percival[103], Stephen J. Perkins[104], Alexandre Persu[105, 106], William G Petchey[107], Matthew C. Pickering[46], Jennifer Pinney[81], David Pitcher[23], Lucy Plumb[43], Zoe Plummer[23], Joyce Popoola[1], Frank Post[28], Albert Power[83], Guy Pratt[56], Charles Pusey[46], Ria Rabara[22], May Rabuya[4],Tina Raju[42], Chadd Javier[108], Ian SD Roberts[22], Candice Roufosse[109], Adam Rumjon[28], Alan Salama[61], Moin Saleem[43], RN Sandford[97], Kanwaljit S. Sandu[110], Nadia Sarween[81], John A. Sayer[95], Neil Sebire[111, 112], Haresh Selvaskandan[26], Sapna Shah[28], Asheesh Sharma[57], Edward J Sharples[22], Neil Sheerin[25], Harish Shetty[53], Rukshana Shroff[112], Roslyn Simms[70], Manish Sinha[2], Smeeta Sinha[113], Kerry Smith[29], Lara Smith[15], Shalabh Srivastava[114], Retha Steenkamp[23], Ian Stott[115], Katerina Stroud[97], Pauline Swift[42], Justyna Szklarzewicz[26], Fred Tam[46], Kay Tan[116], Robert Taylor[117], Marc Tischkowitz[97], Kay Thomas[4], Yincent Tse[118], Alison Turnbull[85], A Neil Turner[119], Kay Tyerman[55], Miranda Usher[120], Gopalakrishnan Venkat-Raman[121], Alycon Walker[122], Stephen B. Walsh[61], Aoife Waters[123], Angela Watt[68], Phil Webster[6], Ashutosh Wechalekar[124], Gavin Iain Welsh[43], Nicol West[125], David Wheeler[61], Kate Wiles[27], Lisa Willcocks[107,] Angharad Williams[33], Emma Williams[29], Karen Williams[4], Deborah H Wilson[126], Patricia D Wilson[127], Paul Winyard[9],

Edwin Wong[25], Katie Wong[23], Grahame Wood[58], Emma Woodward[15], Len Woodward[128], Adrian Woolf[129], David Wright[71]

[1]St George's University Hospitals NHS Foundation Trust, UK

[2]Evelina London Children's Hospital, UK

[3]David Evans Medical Research Centre, Nottingham University Hospital NHS Trust, UK

[4]Guy's and St Thomas NHS foundation Trust, UK

[5]Ysbyty Gwynedd, Betsi Cadwaladr University Health Board, UK

[6]Imperial College Healthcare NHS Trust, UK

[7]James Paget University Hospital NHS Foundation Trust, UK

[8]Heart of England NHS Foundation Trust, Birmingham, UK

[9]Mid and South Essex NHS Foundation Trust, UK

[10]Royal Berkshire NHS Foundation Trust, UK

[11]Bradford Teaching Hospitals NHS Foundation Trust, UK

[12]Royal United Hospital Bath NHS Trust, UK

[13]Freeman Hospital, Newcastle Upon Tyne, UK

[14]Birmingham Women's and Children's NHS Foundation Trust, UK

[15]Manchester University NHS Foundation Trust, UK

[16]Royal Devon University Healthcare NHS Foundation Trust, UK

[17]Medizinische Genetik Mainz, Mainz, Germany

[18]Department of Medicine, Faculty of Medicine, Medical Center-University of Freiburg, Freiburg, Germany

[19]Hull University Teaching Hospitals NHS Trust, UK

[20]Exeter Kidney Unit, Royal Devon University Healthcare NHS Foundation Trust, UK

[21]Gloucestershire Hospitals NHS Foundation Trust, UK

[22]Oxford University Hospitals NHS Foundation Trust, UK

[23]UK Kidney Association, UK

[24]Countess of Chester NHS Foundation Trust, UK

[25]National Renal Complement Therapeutics Centre, Newcastle upon Tyne Hospitals NHS Foundation Trust, Newcastle upon Tyne, UK

[26]University Hospitals of Leicester NHS Trust, UK

[27]Barts Health NHS Trust, London, UK

[28]King's College Hospital NHS Foundation Trust, UK

[29]East Suffolk and North Essex NHS Foundation Trust, UK

[30]Ninewells Hospital and Medical School, Dundee, UK

[31]North West Anglia NHS Foundation Trust, UK

[32]Northern Health and Social Care Trust and Northern Ireland Clinical Research Network

[33]West Suffolk NHS Foundation Trust, UK

[34]Morriston Hospital, Swansea Bay Health Board, UK

[35]Lister Hospital, East and North Hertfordshire NHS Trust, UK

[36]Dartford and Gravesham NHS Trust, UK

[37]Nottingham Children's Hospital, UK

[38]Salford Royal Hospital, Northern Care Alliance NHS Foundation Trust, Salford, UK

[39]University of Manchester, UK

[40]King's College London, UK

[41]Nottingham University Hospitals NHS trust, UK

[42]Epsom and St Helier University Hospitals NHS Trust, UK

[43]University of Bristol Medical School, Bristol, UK

[44]Royal Stoke University Hospital, UK

[45]Manchester Royal Infirmary, UK

[46]Centre for Inflammatory Disease, Imperial College London, UK

[47]Nephrotic Syndrome Trust' (NeST) , UK

[48]North Cumbria Integrated Care NHS Foundation Trust, UK

[49]Colchester General Hospital, UK

[50]Tameside and Glossop Integrated Care NHS Foundation Trust, UK

[51]Wye Valley NHS Trust, UK

[52]BHF Centre for Cardiovascular Science, The Queen's Medical Research Institute, University of Edinburgh, UK

[53]Lancashire Teaching Hospital, UK

[54]School of Medicine, University of Nottingham, UK

[55]Leeds Teaching Hospitals NHS Trust, UK

[56]University of Birmingham, UK

[57]Liverpool University Hospitals Foundation NHS Trust, UK

[58]Salford Royal NHS Foundation Trust, UK

[59]Department of Clinical Genetics, Guy's and St Thomas' NHS Foundation Trust, UK

[60]Centre for Health and Related Research, School of Population Health, University of Sheffield, UK

[61]University College London Department of Renal Medicine, Royal Free Hospital, UK

[62]SW Thames Renal Unit, Epsom and St Helier University Hospitals NHS Trust, UK

[63]Alport UK, UK
[64]Queen Elizabeth University Hospital, Glasgow, UK
[65]College of Medicine and Health, University of Exeter, UK
[66]Divison of Population Health, University of Sheffield, UK
[67]University of Wolverhampton, UK
[68]Patient Representative, UK
[69]Institute of Liver Studies, King's College London, UK
[70]Sheffield Kidney Institute, Sheffield Teaching Hospitals NHS Foundation Trust, UK
[71]Royal Free Hospital, UK
[72]Manchester Institute of Nephrology and Transplantation, Manchester Royal Infirmary, UK
[73]PKD Charity, UK
[74]University Hospital Southampton NHS Foundation Trust, UK
[75]Children's Kidney centre, University Hospital of Wales, UK
[76]Travere Therapeutics, UK
[77]University Hospitals Coventry and Warwickshire NHS Trust, UK
[78]County Durham & Darlington NHS Foundation Trust, UK
[79]University of Leicester, UK
[80]Wirral University Teaching Hospital NHS Foundation Trust, UK
[81]University Hospitals Birmingham NHS Foundation Trust, UK
[82]Department of Medicine, University of Cambridge, UK
[83]North Bristol NHS Trust, UK
[84]Alder Hey Childrens NHS Foundation Trust, UK
[85]York & Scarborough Teaching Hospitals NHS Foundation Trust, UK
[86]Norfolk and Norwich University Hospitals NHS Trust, UK
[87]Royal Manchester Children's Hospital, Manchester, UK
[88]PTEN UK and Ireland Patient Group
[89]HNF1B Support Group, UK
[90]School of Medicine, Keele University, UK
[91]Wellcome Centre for Cell-Matrix Research, University of Manchester, UK
[92]Research Department of Pathology, University College London, UK
[93]HLRCC Foundation, UK
[94]Cambridge Institute of Therapeutic Immunology and Infectious Disease, Cambridge, UK
[95]Newcastle University, UK

[96]United Lincolnshire Hospitals NHS Trust, UK

[97]Department of Medical Genetics, University of Cambridge, UK

[98]University Hospitals of Derby and Burton NHS Foundation Trust, UK

[99]Retroperitoneal Fibrosis (RF) Group, UK

[100]National Institute of Health and Care Research Leeds Biomedical Research Centre, Leeds Teaching Hospitals NHS Trust, UK

[101]School of Medicine, University of Leeds, UK

[102]University of Liverpool, UK

[103]Newcastle Upon Tyne Hospitals NHS Foundation Trust, UK

[104]Research Department of Structural and Molecular Biology, University College London, UK

[105]Division of Cardiology, Cliniques Universitaires Saint-Luc, Belgium

[106]Pole of Cardiovascular Research, Institut de Recherche Expérimentale et Clinique, Université Catholique de Louvain, Brussels, Belgium

[107]Cambridge University Hospitals NHS Foundation Trust, UK

[108]East and North Hertfordshire NHS Trust, UK

[109]Department of Immunology and Inflammation, Faculty of Medicine, Imperial College London, UK

[110]Shrewsbury and Telford Hospital NHS Trust, UK

[111]National Institute of Health and Care Research Great Ormond Street Hospital Biomedical Research Centre, UK

[112]UCL Great Ormond Street Institute of Child Health, UK

[113]Northern Care Alliance NHS Foundation Trust, UK

[114]South Tyneside and Sunderland NHS Foundation Trust, UK

[115]Doncaster and Bassetlaw Teaching Hospitals, UK

[116]New Cross Hospital, Wolverhampton, UK

[117]Wellcome Centre for Mitochondrial Research, Translational & Clinical Research Institute, Faculty of Medical Sciences, Newcastle University, UK

[118]Great North Children's Hospital, Newcastle Upon Tyne, UK

[119]University of Edinburgh, UK

[120]Calderdale & Huddersfield Foundation Trust, UK

[121]Royal Surrey County Hospital, Guildford, UK

[122]South Tees Hospitals NHS Foundation Trust, UK

[123]University College Cork, Ireland

[124]National Amyloidosis Centre, University College London, UK

[125]Great Western Hospital, Swindon, UK

[126]North Tees and Hartlepool NHS Foundation Trust, UK

[127]University College London, UK

[128]aHUS Alliance, UK

[129]School of Biological Sciences, University of Manchester, UK
